# Histone lactylation‐mediated up‐regulation of IGF2BP2 enhances ferroptosis resistance via Nrf2 in colorectal cancer

**DOI:** 10.1002/ctm2.70551

**Published:** 2025-12-15

**Authors:** Jin‐Feng Zhu, Da‐Peng Guo, Hai‐Na Lv, Zong‐Yu Liang, Jing Song, Wei Zeng

**Affiliations:** ^1^ Department of Gastrointestinal Surgery, the Sixth Affiliated Hospital, School of Medicine South China University of Technology Foshan Guangdong China; ^2^ Department of Radiology, the Sixth Affiliated Hospital, School of Medicine South China University of Technology Foshan Guangdong China; ^3^ Department of Respiratory medicine, the Sixth Affiliated Hospital, School of Medicine South China University of Technology Foshan Guangdong China; ^4^ Cancer Center of The Sixth Affiliated Hospital School of Medicine, South China University of Technology Foshan Guangdong China

**Keywords:** ferroptosis resistance, IGF2BP2, lactate, macrophage polarisation, Nrf2

## Abstract

**Background:**

Emerging evidence suggests that ferroptosis resistance may drives colorectal cancer (CRC) pathogenesis and limits therapeutic efficacy. A previous study has reported that lactate can enhance ferroptosis in CRC cells. The objective of this study was to elucidate how lactate regulates ferroptosis in CRC and to identify potential therapeutic targets.

**Methods:**

Cellular viability and proliferative capacities were determined via cell counting kit‐8 (CCK‐8) and colony formation. Ferroptosis‐related and inflammatory markers, including malondialdehyde (MDA), Fe^2+^, glutathione (GSH), IL‐1β, IL‐12 and IL‐10, were quantified by commercial kits. Protein and RNA interactions were investigated using co‐immunoprecipitation (Co‐IP), RNA pull‐down, dual‐luciferases reporter and RNA immunoprecipitation （RIP) assays. Flow cytometry analysed M1 and M2 macrophage populations. Chromatin immunoprecipitation followed by quantitative polymerase chain reaction (ChIP–qPCR) examined histone H3 lysine 18 lactylation (H3K18la) and EP300 binding at the insulin‐like growth factor 2 mRNA‐binding protein 2 (IGF2BP2)promoter. Methylated RNA Immunoprecipitation‐qPCR (MeRIP–qPCR) measured the m^6^A modification of nuclear factor erythroid 2‐related factor 2 (Nrf2) mRNA. Transmission electron microscopy determined mitochondrial morphology. C11‐BODIPY immunofluorescence staining analysed lipid peroxidation.

**Results:**

Our findings revealed that lactate up‐regulates IGF2BP2 through H3K18la‐mediated transcriptional activation in both CRC cells and tumour‐associated macrophages. Elevated IGF2BP2 directly bound to and stabilised Nrf2 mRNA, resulting in increased Nrf2 levels and enhanced resistance to ferroptosis in CRC cells. Notably, this lactate–IGF2BP2–Nrf2 axis also promoted M2 macrophage polarisation, fostering an immunosuppressive tumour microenvironment (TME). In xenograft models, lactate‐driven Nrf2 up‐regulation accelerated CRC tumour growth and metastasis. Conversely, pharmacological inhibition of ferroptosis resistance with dichloroacetate (DCA) or depletion of IGF2BP2 significantly reduced tumour burden.

**Conclusion:**

Our study identified a novel lactate–IGF2BP2–Nrf2 signalling pathway that drives ferroptosis resistance and immune evasion in CRC.

**Key points:**

- Lactate drives H3K18la‐mediated transcriptional activation of IGF2BP2 in CRC.
- IGF2BP2 protein stabilises Nrf2 mRNA by binding to its m^6^A modification site.
- The lactate–IGF2BP2–Nrf2 axis confers ferroptosis resistance in CRC cells.
- This pathway simultaneously promotes M2 macrophage polarisation in the tumour microenvironment.

## Graphical abstract

In the lactate‐rich tumor microenvironment, p300‐mediated H3K18la epigenetically activates IGF2BP2 transcription. The IGF2BP2 protein then stabilizes Nrf2 mRNA in the cytoplasm, leading to increased Nrf2 levels. This axis simultaneously confers ferroptosis resistance to cancer cells and promotes M2 macrophage polarization, fostering a malignant, treatment‐resistant tumor microenvironment.

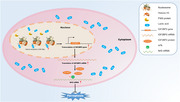

## 1 INTRODUCTION

Colorectal cancer (CRC) continues to pose a significant global health burden, with steadily increasing disease prevalence and death toll. Therapeutic progress in CRC management—ranging from surgical interventions to systemic drug regimens—has improved survival outcomes.^1^ However, these treatment options have limited impact on long‐term survival.^2^ Ferroptosis, an iron‐dependent form of regulated cell death, has attracted intense research interest for its potential in cancer treatment.^3^ The design and development of ferroptosis inducers as novel anti‐cancer agents is actively being pursued across various solid tumour types, such as lung adenocarcinoma^4^ and triple‐negative breast cancer.^5^ In CRC, rapid progress has been made in elucidating the mechanisms of ferroptosis and in identifying valuable therapeutic targets. For example, aspirin has been shown to sensitise CRC cells with oncogenic PIK3CA mutations to ferroptosis by inhibiting AKT/mTOR signalling, demonstrating promising therapeutic potential.^6^ Nevertheless, cancer cells exhibit differential ferroptotic susceptibilities depending on their distinct metabolic states. A better understanding of molecular determinants that modulate ferroptosis susceptibility in CRC is urgently required to improve treatment outcomes.

A critical consequence of the unique metabolic mode of cancer cells is the creation of a nutrient‐deprived, hypoxic and acidic tumour microenvironment (TME), which poses a significant barrier to ferroptosis induction.^7^ Lactate is the main metabolic product of aerobic glycolysis, which facilitates cancer cell survival and growth.^8^ However, increasing publications have indicated that lactate is not merely a metabolic byproduct but a nutrient with numerous regulatory functions in the TME,^9^ especially in relation to oxidative stress resistance and lipid biosynthesis.^10^, ^11^ A previous study has suggested that lactate‐rich liver cancer cells exhibit increased resistance to ferroptosis by promoting ATP production and deactivating AMPK.^12^ Interestingly, lactate dehydrogenase A (LDHA) enhances ferroptosis in CRC cells and strengthens CD8+ T cell anti‐tumour activity.^13^ Therefore, it is worthwhile to elucidate how lactate shapes ferroptosis in CRC microenvironment.

Lactylation, a post‐translational modification driven by lactate, has emerged as a crucial epigenetic regulator in cancer, modulating critical genes that govern tumour growth, metastasis and therapeutic sensitivity.^14^, ^15^ Interestingly, lactate influences macrophage polarisation,^16^ a process exerting dual effects on tumour progression. While M1 macrophages exhibit pro‐inflammatory and tumour‐suppressive functions, their M2 counterparts promote tumour growth, angiogenesis and immune evasion.^17^ Previous study has indicated that an elevated M1:M2 ratio within CRC tumour stroma correlates with more favourable survival outcomes,^18^ highlighting the utility of assessment of macrophage polarisation. Notably, tumour‐associated macrophages (TAMs), which predominantly exhibit an M2‐like phenotype, are not only a major source of lactate in the TME but also undergo functional reprogramming in response to lactate.^19^ Critically, lactate has been shown to directly promote M2 polarisation, enhancing their pro‐tumourigenic functions,^20^ a process potentially regulated by key transcriptional programs.

Nuclear factor erythroid 2‐related factor 2 (Nrf2) is a transcription factor composed of seven structurally distinct Nrf2–ECH (Neh) domains that regulate the transcription of a battery of cytoprotective gene. In CRC, Nrf2 is frequently overexpressed and associated with tumour malignant progression.^21^, ^22^ Moreover, Nrf2 is a key mediator of ferroptosis resistance. For instance, Nrf2 protects hepatocellular carcinoma cells against ferroptosis through the p62/Keap1 pathway, thereby inhibiting the anti‐cancer activity of erastin and sorafenib.^23^ Yang et al. indicated that cetuximab enhances RSL3‐induced ferroptosis and alleviates CRC development by inhibiting the Nrf2/HO‐1 axis.^24^ More interestingly, Nrf2 has a unique role in macrophages, where it can suppress inflammatory responses by inhibiting the transcription of pro‐inflammatory cytokines, thereby limiting the antigen presentation and innate immune capabilities of M1 macrophages in the TME.^25^ Consequently, targeting Nrf2‐mediated resistance to ferroptosis and macrophage polarisation has been proposed as a promising therapeutic strategy for CRC.

RNA modification‐mediated post‐transcriptional regulation has emerged as a critical mechanism governing gene expression and cellular processes in both cancer and immune cells within the TME.^26^ In eukaryotic cells, N6‐methyladenosine (m^6^A) serves as the predominant mRNA modification that orchestrates transcript stability, translation and splicing.^27^ The m^6^A reader protein insulin‐like growth factor 2 mRNA‐binding protein 2 (IGF2BP2) recognises and binds to m^6^A‐modified transcripts, thereby regulating their fate and functionality.^28^ In addition, IGF2BP2 has been implicated in promoting cancer progression, including CRC. For example, circEZH2 enhanced the stability of m^6^A‐modified CREB1 mRNA by interacting with IGF2BP2, thus aggravating CRC progression.^29^ Beyond its oncogenic roles in CRC, IGF2BP2 is increasingly recognised as a key regulator in immune cells. For instance, IGF2BP2 participates in regulating macrophage‐mediated pancreatic cancer progression by stabilising oncogenic transcripts in an m^6^A‐dependent manner.^30^ Notably, IGF2BP2 confers resistance to ferroptosis in malignancies through modulating core protective factors like glutathione peroxidase 4 (GPX4).^31^ Intriguingly, emerging evidence has solidified the role of the m^6^A–IGF2BP2 axis in regulating Nrf2. A recent seminal study demonstrated a direct mechanistic link: IGF2BP2 interacts with and stabilises Nrf2 mRNA, thereby enhancing GPX4 expression and conferring ferroptosis resistance.^32^ This finding is strongly supported by earlier work showing that knockdown of ALKBH5 leads to up‐regulation of Nrf2 and increases the resistance of hypopharyngeal squamous cell carcinoma cells to ferroptosis in an m^6^A–IGF2BP2‐dependent manner.^33^ Critically, Nrf2 activation is not only a mechanism of ferroptosis resistance but also a driver of M2‐like macrophage polarisation and immunosuppression.^34^ This convergence of pathways led us to investigate a novel regulatory axis. Our preliminary data demonstrated a marked elevation in IGF2BP2 lactylation in both CRC cells and macrophages following lactate induction. Strikingly, treatment with oxamate, a widely used inhibitor of histone H3 lysine 18 lactylation (H3K18la), markedly down‐regulates IGF2BP2. Given (1) the role of IGF2BP2 in regulating Nrf2 stability, (2) the ability of Nrf2 to drive both ferroptosis resistance and M2 polarisation and (3) the lactate‐rich nature of the CRC TME, we hypothesised that lactate‐induced IGF2BP2 lactylation stabilises Nrf2 to simultaneously enforce a ferroptosis‐resistant and immunosuppressive phenotype specifically in M2 macrophages. Elucidating this interplay is crucial for developing novel therapeutic strategies that simultaneously target tumour intrinsic resistance and immune evasion in CRC.

This investigation elucidates how lactate‐mediated histone lactylation regulates IGF2BP2 expression to stabilise Nrf2, thus promoting both ferroptosis protection and M2 polarisation in CRC. The results establish new paradigms for epigenetic therapy in chemoresistant CRC.

## 2 MATERIALS AND METHODS

### 2.1 Bioinformatics analysis and public data

*TCGA and GEO Data Analysis*: Publicly available RNA‐sequencing data (Level 3 TPM, log2‐transformed) and clinical information for the colorectal adenocarcinoma (COAD) cohort were downloaded from The Cancer Genome Atlas (TCGA) database via the GDC Data Portal (https://portal.gdc.cancer.gov/). This dataset was used for differential expression analysis of IGF2BP2 (*n* = 275 tumour tissues vs. *n* = 349 adjacent normal tissues; Figure S1C) and for correlation analyses between Nrf2 and IGF2BP family members (*n* = 275; Figure S1D–F). Additional gene expression data were obtained from the Gene Expression Omnibus (GEO) database (https://www.ncbi.nlm.nih.gov/geo/), specifically dataset GSE24550, to analyse IGF2BP2 and Nrf2 expression in matched COAD and normal tissues (Figure S1A).

*Web‐Based Analysis Tools*: The Gene Expression Profiling Interactive Analysis 2 (GEPIA2) web server (http://gepia2.cancer‐pku.cn/) was utilised to perform disease‐free survival (DFS) analysis based on IGF2BP2 and Nrf2 expression (Figure S1B) and to analyse the expression profiles of candidate RBPs (HNRNPL, IGF2BP1, IGF2BP2, IGF2BP3, PABPC4, SRSF1 and SRSF9) in the COAD cohort (Figure S7B).

*In Silico Prediction of RBPs and Binding Sites*: Potential Nrf2 mRNA‐binding RNA‐binding proteins (RBPs) were predicted by intersecting the results from the starBase (https://starbase.sysu.edu.cn/) and cisbp‐RNA (http://cisbp‐rna.ccbr.utoronto.ca/) databases (Figure S7A). Putative IGF2BP2 binding motifs were identified using the RM2Target database (http://rm2target.canceromics.org/) (Figure S14A). The potential m^6^A methylation site on Nrf2 mRNA was predicted using the deepSRAMP database (http://www.cuilab.cn/sramp/) (Figure S14B).

### 2.2 Clinical samples

Clinical specimens comprising matched tumour and adjacent normal tissues were acquired from CRC patients undergoing surgical resection at the Sixth Affiliated Hospital, School of Medicine, South China University of Technology. This study was conducted with institutional review board approval, and written informed consent was obtained from all subjects. Clinicopathological characteristics of all CRC patients was presented in the Table S1.

### 2.3 Cell culture and treatment

HEK293T cells, human CRC cells (SW480, Caco‐2, LoVo, LS513, HCT116, SW620, SW1116 and HT‐29) along with NCM460 normal colonic epithelial controls from ATCC (Manassas, VA, USA) were maintained in standard conditions (DMEM with 10% FBS, 1% antibiotics, 37°C, 5% CO_2_). For treatments, CRC cells were exposed to lactate (L7022, 0, 1, 5, 10 or 20 mM; Sigma–Aldrich), RSL3 (0, .5, 1, 5 or 10 µM, S8155; Selleckchem), CWI1‐2 (5 mM, E1327; Selleckchem), 3‐hydroxy‐butyrate (3‐OBA, 5 mM; Sigma), 3‐OBA (10 µM), erastin (10 µM, 329600–5MG; Sigma–Aldrich), ferrostatin‐1 (Fer‐1, 1 µM, S7243; Selleckchem), Z‐VAD‐FMK (50 µM, S7023; Selleckchem), necrostatin‐1 (Nec‐1, 30 µM, S8037; Selleckchem), VX‐765 (20 µM, S2228; Selleckchem), dichloroacetic acid (DCA; 20 µM, D54702; Sigma–Aldrich), AZ‐33 (.5 µM, HY‐112229; MCE) or ML385 (5 µM, S8693; Selleckchem) for indicated durations.

### 2.4 Cell transfection

IGF2BP2‐ or Nrf2‐targeted shRNA (shIGF2BP2‐1, 5′‐CACCATCGAGACCCTCTCGGGTAAAC

GAATTTACCCGAGAGGGTCTCGA‐3′; shIGF2BP2‐2, 5′‐CACCGCTGTTAACCAACAAGCC

AATCGAAATTGGCTTGTTGGTTAACAGC‐3′; shNrf2‐1, 5′‐CACCGCCGGCATTTCACTAA

ACACAACGAATTGTGTTTAGTGAAATGCCGG‐3′; shNrf2‐2, 5′‐CACCGCACCTTATATCT

CGAAGTTTCGAAAAACTTCGAGATATAAGGTGC‐3′) purchased from Fenghbio (Changsha, China) was inserted into pGLVH1 vector, Nrf2/IGF2BP2 cDNA was subcloned into pcDNA3.1 (Invitrogen, Carlsbad, CA, USA) to generate the overexpression construct, while EP300‐specific siRNA (si‐EP300, targeting sequence: 5′‐CAATTCCGAGACATCTTGAGA‐3′) and GPR81‐specific siRNA (si‐GPR81, targeting sequence: 5′‐AAAUUGAAAAGGUAAACAGUG‐3′) were obtained from Gene (Shanghai, China). Plasmid delivery into cells was carried out using Lipofectamine 3000. For stable knockdown studies, lentiviral particles expressing shIGF2BP2 were generated in HEK293T cells co‐transfected with shRNA constructs and packaging plasmids (psPAX2 and pMD2.G) using Lipofectamine 3000. Virus supernatants collected at 48–72 h post‐transfection were passed through .45‐µm membranes and applied to target cell transduction with 8 µg/mL polybrene. Puromycin selection was performed to enrich for stably transduced cell populations.

To generate stable IGF2BP2 knockout (KO) cell lines, sgRNA sequences targeting the IGF2BP2 gene were designed by GenScript (Nanjing, China). The following sequences were selected: sgIGF2BP2‐1 (5′‐AGTGGTCCACCCCCACCACG‐3′) and sgIGF2BP2‐2 (5′‐TTACCTTTTCAATTTGGCCG‐3′). These sgRNA sequences were cloned into the lentiviral vector pLentiCRISPRv2. Lentivirus production and transduction were performed as described above. Following transduction, SW480 and Caco‐2 cells were selected using puromycin (e.g., 2 µg/mL for 72 h). Single‐cell clones were isolated by limiting dilution to ensure clonality, or stable polyclonal populations were established. The KO efficiency was validated by Western blot analysis.

### 2.5 Quantitative real‐time polymerase chain reaction (RT‐qPCR)

RNA was extracted using TRIzol reagent (Invitrogen) followed by cDNA synthesis using PrimeScript RT‐PCR master mix (Takara). Gene expression was quantified via SYBR green‐based qPCR with GAPDH normalisation. Relative expression was calculated using the 2^–ΔΔCt^ method, with primers listed in the Table S2.

### 2.6 Western blot analysis

Protein lysates were prepared in radioImmunoprecipitation assay (RIPA) buffer containing a protease/phosphatase inhibitor cocktail and separated by SDS‐PAGE. Following transfer to PVDF membranes, targeted proteins were determined by antibodies against: IGF2BP2 (ab124930; Abcam), Nrf2 (PA5‐27882; Thermo Fisher), GPX4 (ab125066; Abcam), H3K18la (Cat#22H2L3; PTM BIO), EP300 (33‐7600; ThermoFisher), GPR81 (ab106942; Abcam) and β‐actin (ab8226; Abcam), with visualisation by HRP‐conjugated secondary antibodies.

### 2.7 Macrophage and CRC cells co‐culture

Macrophage differentiation was induced in the human monocytic THP‐1 cell line (ATCC) by 24‐h treatment with phorbol 12‐myristate 13‐acetate (PMA; 200 nM, P8139; Sigma). To investigate macrophage–CRC cell crosstalk, both direct and indirect co‐culture systems were established. For direct co‐culture, differentiated THP‐1‐derived macrophages were detached and seeded directly onto confluent monolayers of SW480 or Caco‐2 cells at a ratio of 1:2 (macrophage:CRC cells) in complete DMEM medium. For indirect co‐culture, Transwell inserts (3413; Corning) were used, with macrophages seeded in upper chamber while CRC cells in lower chamber, or CRC cells seeded in upper chamber while macrophages in lower chamber. All co‐culture and macrophages from lower chamber were conducted for 48 h prior to subsequent analysis of macrophage polarisation markers by flow cytometry, qRT‐PCR and ELISA, and CRC cells from lower chamber were collected to evaluate CRC cell proliferation, colony formation, migration and invasion capabilities.

### 2.8 Cell‐Counting‐Kit‐8 (CCK‐8) assay

Cells plated in 96‐well plates were allowed to adhere overnight and incubated with 10 µL CCK‐8 reagent (Beyotime, China) for 2 h at 37°C. Absorbance was measured at 450 nm using a spectrophotometer (Bio‐Rad, Hercules, CA, USA).

### 2.9 Colony formation assay

Cells (500–1000 cells/well) were sparsely seeded in six‐well plates and allowed to form colonies for 10–14 days with medium refreshed every 3 days. Following incubation, colonies were fixed (4% paraformaldehyde), stained (.5% crystal violet solution), washed, air‐dried and counted.

### 2.10 Analysis of oxidative stress markers and lactate

The respective commercial kits were used to determine levels of malondialdehyde (MDA; MAK085; Sigma–Aldrich), Fe^2^⁺ (ab8336; Abcam), lipid reactive oxygen species (ROS) (C10446; Invitrogen), glutathione (GSH; ab138881; Abcam) and lactate (ab65331; Abcam). All procedures were strictly performed in accordance with the manufacturers' protocols.

### 2.11 Cytokine quantification by ELISA

Levels of IL‐1β (DY201), IL‐12 (DY219) and IL‐10 (DY217B) in culture supernatants were measured using ELISA kits (all purchased from R&D Systems).

### 2.12 Transmission electron microscopy (TEM)

We performed TEM sample preparation by fixing cells in 2.5% glutaraldehyde and then post‐fixing them in 1% osmium tetroxide. Following dehydration through graded ethanol series, samples were infiltrated with epoxy resin and polymerised at 60°C for 24 h. Ultrathin sections (60–80 nm) were prepared with an ultramicrotome, contrasted with uranyl acetate and lead citrate and imaged on a JEOL JEM‐1400Plus instrument operating at 80 kV. Mitochondrial morphology was assessed by examining at least 100 mitochondria from ≥10 cells per group.

### 2.13 C11‐BODIPY lipid peroxidation analysis

Following a 30‐min incubation with 2 µM C11‐BODIPY (Invitrogen; D3861) in serum‐free medium at 37°C in the dark, cells were washed with PBS and fixed with 4% paraformaldehyde. After permeabilisation (.1% Triton X‐100) and blocking (5% BSA), nuclei were counterstained with DAPI (1 µg/mL). Subsequently, coverslips were mounted with anti‐fade medium and imaged on an Olympus IX73 fluorescence microscope. The oxidised and non‐oxidised forms of the probe were detected by their green (488/510 nm) and red (581/591 nm) fluorescence, respectively.

### 2.14 Flow cytometry

Macrophage subtypes were evaluated using fluorochrome‐labelled antibodies specific for CD11b, CD86 (M1) and CD206 (M2), with subsequent analysis on a FACSCalibur flow cytometer (BD Biosciences).

### 2.15 Chromatin immunoprecipitation (ChIP)

Cells underwent formaldehyde (1%) cross‐linking followed by glycine quenching and lysis. Chromatin was isolated, fragmented using a micrococcal nuclease and immunoprecipitated overnight (4°C) with ChIP‐grade antibodies against H3K18la (22H2L3, PTM BIO), EP300 (33‐7600; Thermo Fisher) or control rabbit IgG (Cat#2729; Cell Signaling Technology). Protein G agarose beads were used to capture the antibody‐chromatin complexes, followed by cross‐link reversal and DNA purification. IGF2BP2 promoter occupancy was analysed by RT‐qPCR using primers targeting seven different segments a–g detailed in Table S3.

### 2.16 m^6^A RNA immunoprecipitation(MeRIP)

MeRIP assays were performed using the EpiMark N6‐Methyladenosine Enrichment Kit (E1610S; New England Biolabs). Briefly, total RNA extracted with TRIzol was fragmented into 100–300 nucleotide segments by incubation with fragmentation buffer at 94°C for 3 min, then immunoprecipitated with either anti‐m^6^A antibody (ab208577; Abcam) or control IgG (2729; Cell Signaling Technology). The antibody–RNA complexes were captured using protein A/G magnetic beads, followed by washing to remove unbound RNA. The immunoprecipitated RNA was eluted from the beads and purified using the RNA Clean & Concentrator Kit (R1015; Zymo Research). The enrichment of m^6^A modification on Nrf2 mRNA was analysed by RT‐qPCR.

### 2.17 RNA pull‐down

To map the IGF2BP2 binding sites on Nrf2 mRNA, biotin‐labelled RNA probes spanning the full‐length Nrf2 transcript were synthesised using the BioTin 3′ End DNA Labeling Kit (20160; Thermo Fisher). The biotin‐labelled RNA was incubated with whole‐cell lysates prepared in RIP buffer. RNA–protein complexes were captured using Dynabeads MyOne Streptavidin C1 beads (65002; Invitrogen) and washed with RIP buffer. After eluting, IGF2BP2 protein were assessed by western blot. To determine the Nrf2 mRNA regions required for IGF2BP2 binding, DNA templates for in vitro transcription were generated by PCR amplification of the 3′ untranslated region (UTR), 5′ UTR and coding sequence (CDS) of Nrf2 mRNA, with either wild‐type (WT) or mutant (Mut) sequences harbouring point mutations. Biotinylated RNA probes (3′UTR–WT, 5′UTR–WT, CDS–WT, 3′UTR–Mut, 5′UTR–Mut, CDS–Mut) were synthesised using the MEGAscript T7 Transcription Kit (AM1334; Invitrogen) and used in RNA pulldown assays as described above.

### 2.18 RNA immunoprecipitation (RIP)

RNA–protein interactions were assessed using Magna RIP Kit (17‐700; Millipore). Briefly, cell extracts in RIP lysis buffer underwent immunoprecipitation with anti‐IGF2BP2 antibody (ab124930; Abcam) or control IgG using protein A/G magnetic beads. Following washing and elution steps, RNA was purified and analysed for Nrf2 mRNA enrichment by RT‐qPCR. To determine the Nrf2 mRNA‐binding domains of IGF2BP2, truncation mutants of IGF2BP2 were generated, encompassing four K‐homology (KH) RNA‐binding domains, either individually or in combination (KH1, KH1‐2, KH1‐3, KH2‐3, KH3‐4 and KH4). The truncated proteins were expressed and purified from the bacteria for use in RIP assays as described above.

### 2.19 Dual‐luciferase reporter assay

To elucidate the IGF2BP2–Nrf2 mRNA interaction, reporter vectors were constructed by cloning the Nrf2 CDS into the pGL3‐Basic vector. These included a WT version, which contained the intact IGF2BP2‐binding ‘AAACT’ motif at position 2109, and a Mut variant with an A‐to‐T substitution to abrogate binding. All constructs were validated by Sanger sequencing. Next, cells were co‐transfected with either Nrf2–WT or Nrf2–Mut reporter constructs and the pRL‐TK Renilla luciferase vector for normalisation. Measurements were taken 48 h post‐transfection with a dual‐luciferase assay kit. Results are presented as the ratio of firefly to Renilla luciferase activity.

To assess the role of histone H3K18 modification in regulating IGF2BP2 transcription, a separate reporter assay was performed. The putative IGF2BP2 promoter region was cloned into the pGL3‐Basic vector. HEK293T cells were co‐transfected with this firefly luciferase reporter, the pRL‐TK control vector and either a H3–WT or a H3K18R–Mut expression plasmid (pcDNA3.1 background). Luciferase activity was quantified after 48 h; firefly luminescence values were standardised to the corresponding Renilla signals.

### 2.20 mRNA stability assessment

Transcriptional shutdown was induced with actinomycin D (5 µg/mL; Sigma‐Aldrich), followed by RNA extraction at 0–4 h timepoints. Nrf2 transcript decay was monitored using RT‐qPCR and normalised to that of GAPDH.

### 2.21 Polysome profiling

Cells were treated with 100 µg/mL cycloheximide for 10 min at 37°C before collection. Cell lysates were prepared in polysome lysis buffer (10 mM Tris–HCl pH 7.4, 150 mM NaCl, 5 mM MgCl_2_, 1% Triton X‐100, 40 U/mL RNase inhibitor and 1 mM DTT) and layered onto 10–50% sucrose gradients. For fractionation, cytoplasmic lysates were loaded onto 5–50% sucrose gradients and separated by ultracentrifugation using a SW41Ti rotor (Beckman Coulter) at 32 000 rpm for 3 h at 4°C. Fractions were collected and RNA was extracted from each fraction and Nrf2 mRNA levels were quantified using RT‐qPCR.

### 2.22 In vivo studies

Animal procedures received approval from the Institutional Animal Care and Use Committee of the Sixth Affiliated Hospital, School of Medicine, South China University of Technology. Male nude mice (BALB/c, 6–8 weeks old) were purchased from human SJA Laboratory Animal Co., LTD (Hunan, China). For the xenograft tumour model, SW480 or Caco‐2 cells stably overexpressing LDHA or empty vector control were established using lentiviral transduction and selected with puromycin. Cells (1 × 10⁶) were subcutaneously injected into the right flank of each mouse. Once palpable tumours formed (∼100 mm^3^), mice received intraperitoneal injections of RSL3 (7.5 mg/kg body weight, every 3 days) or vehicle controls. Experimental groups included OE‐NC, OE‐LDHA, OE‐NC + RSL3 and OE‐LDHA + RSL3. For IGF2BP2 knockdown models, groups included shNC, shIGF2BP2, shNC + RSL3 and shIGF2BP2 + RSL3. For co‐injection models, SW480 or Caco‐2 cells were injected alone or together with THP‐1‐derived macrophages (1 × 10⁶) into nude mice pretreated with clodronate liposomes (100 µL, tail vein, 48 h prior) to deplete endogenous macrophages. Groups included CRC (control), CRC + DCA, CRC + AZ‐33, CRC + DCA + lactate, CRC + MΦ, CRC + MΦ + DCA, CRC + MΦ + AZ‐33 and CRC + MΦ + DCA + lactate. Tumour dimensions were measured using calipers, and volumes were calculated as (length × width^2^)/2.

Metastasis models were established via tail vein injection of CRC cells (5 × 10^5^ in 100 µL PBS). Lung metastases were monitored weekly by bioluminescence imaging on an IVIS Spectrum system (PerkinElmer). Mice were intraperitoneally injected with d‐luciferin (150 mg/kg body weight; GoldBio) and imaged 10–15 min later under isoflurane anaesthesia. Bioluminescence signals were quantified as total flux (photons/s) using Living Image software (PerkinElmer). At the experimental endpoints, mice were euthanised by CO_2_ inhalation to collect tumour and lung tissues.

### 2.23 Histological analyses

Tumour and lung tissues underwent formalin fixation, paraffin embedding and sectioning (5 µm thickness). For histological evaluation, haematoxylin and eosin (H&E) staining was conducted following deparaffinisation and rehydration.

For immunohistochemistry (IHC), after antigen retrieval (citrate buffer, pH 6.0), sections were peroxidase‐quenched and serum‐blocked before overnight incubation with the following primary antibodies: anti‐Ki67 (ab15580; Abcam), anti‐LDHA (ab300638; Abcam), anti‐CD206 (ab64693; Abcam), anti‐CD86 (Cat#14‐0862‐82; Thermo Fisher), anti‐Arg‐1 (ab96183; Abcam), anti‐iNOS (Ab3523; Abcam), anti‐IGF2BP2 (ab124930; Abcam), anti‐Nrf2 (Cat#PA5‐27882; Thermo Fisher) or anti‐GPX4 (ab125066; Abcam). Detection employed biotinylated secondaries with avidin‐biotin complex amplification (PK‐6100; Vector Laboratories) and DAB visualisation (SK‐4105; Vector Laboratories) with haematoxylin counterstaining.

For TSA‐amplified multiplexed immunofluorescence, tumour tissue sections of CRC patients were processed using the Opal 7‐Color Manual IHC Kit (NEL811001KT; Akoya Biosciences) with sequential staining for H3K18la (22H2L3, PTM BIO), IGF2BP2 (ab124930; Abcam), Nrf2 (PA5‐27882; Thermo Fisher) and GPX4 (ab125066; Abcam) antibodies. TSA Plus‐amplified fluorophore labelling was followed by DAPI nuclear counterstaining and mounting in the ProLong Diamond Antifade reagent. Multispectral imaging was performed using a Vectra Polaris system (Akoya Biosciences).

### 2.24 Statistical analysis

Results are presented as mean ± standard deviation. Statistical analyses were performed using GraphPad Prism 8.0, including survival analysis using the Kaplan–Meier method with log‐rank testing, between‐group comparisons using two‐tailed Student's *t*‐test, and multi‐group comparisons employing one‐way ANOVA. Correlation analyses were conducted using Pearson's correlation coefficient unless otherwise specified. Statistical significance was defined as *p* < .05 for all analyses.

## 3 RESULTS

### 3.1 Elevated lactylation, H3K18la and IGF2BP2 levels were associated with Nrf2‐mediated ferroptosis resistance in CRC

**FIGURE 1:**
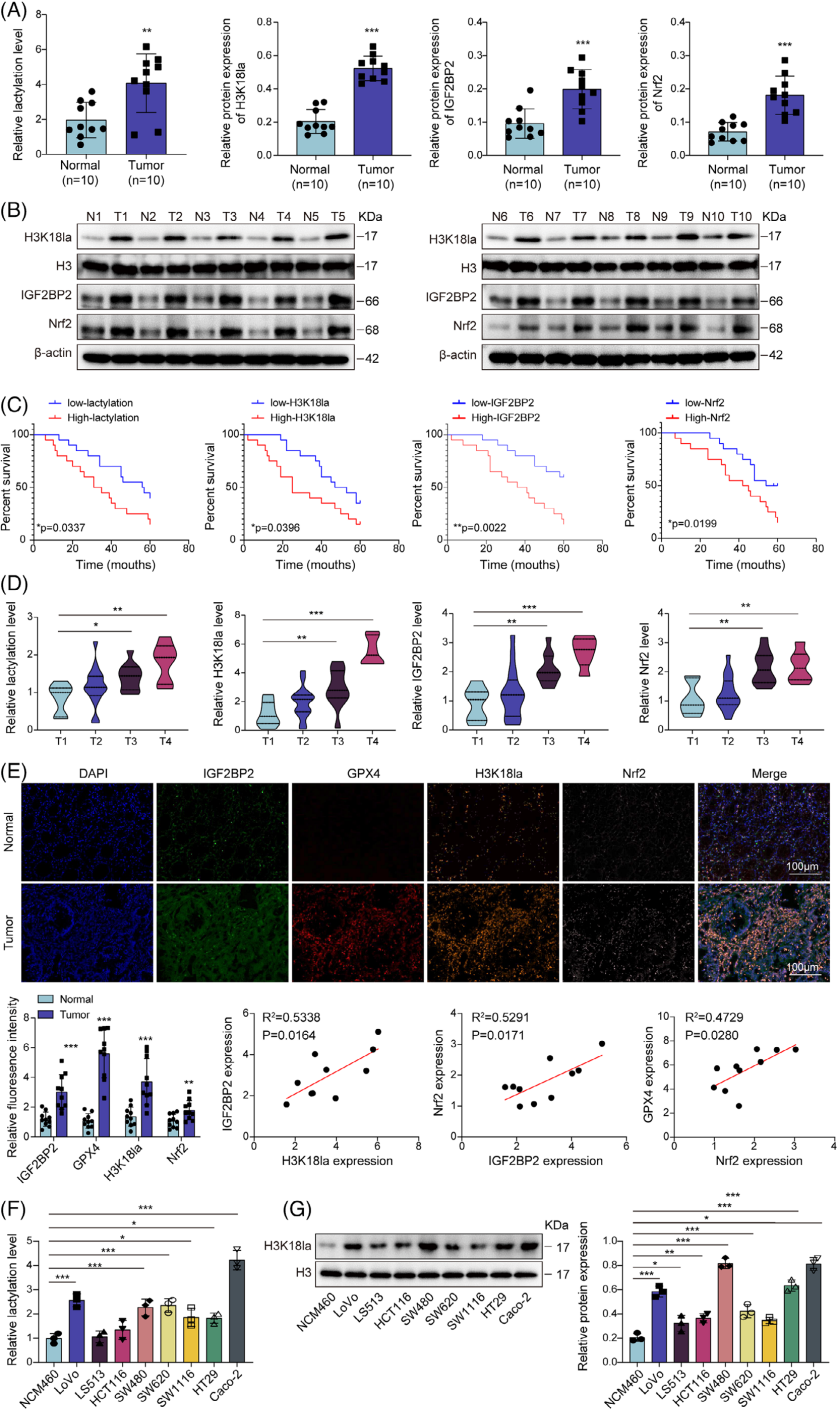
Elevated lactylation, H3K18la and IGF2BP2 levels were associated with Nrf2‐mediated ferroptosis resistance in CRC. (A) Lactylation levels in normal adjacent tissues and patients with CRC (*n* = 10) were analysed. (B) H3K18la, IGF2BP2 and Nrf2 levels in normal adjacent and CRC tissues were examined by western blotting (*n* = 10). (C) Kaplan–Meier survival analysis of normal adjacent tissues and patients with CRC stratified by high/low lactylation, H3K18la, IGF2BP2 and Nrf2 levels (*n* = 40). (D) Lactylation, H3K18la, IGF2BP2 and Nrf2 levels in CRC tissues with different tumour stages (T stages) (*n* = 40). (E) Upper panels: TSA‐amplified multiplexed immunofluorescence of normal adjacent and CRC tissues for H3K18la, IGF2BP2, Nrf2 and GPX4 expression (scale bar = 100 µm). Below panels: quantitative analysis of the fluorescence signals using ImageJ software and correlation analyses between: (left) H3K18la versus IGF2BP2 expression, (middle) IGF2BP2 versus Nrf2 expression and (right) Nrf2 versus GPX4 expression in CRC tissue samples (*n* = 10). (F) Intracellular lactylation levels in the normal colon epithelial cell line NCM460, and eight CRC cell lines (LoVo, LS513, HCT116, SW480, SW620, SW1116, HT29 and Caco‐2) were measured by colorimetric assay. (G) H3K18la levels in these cells were examined using western blot. Measurement data are presented as mean ± SD. *n* = 3 for F and G. *p < .05, **p < .01, ***p < .001.

A previous prior investigation of 330 CRC patients within the SYSUCC cohort indicated a correlation between elevated lactylation and H3K18la levels and unfavourable clinical outcomes.^35^ Here, we also revealed a marked increase in lactylation levels in the CRC tissues (Figure 1A). Analysis of GEO and GEPIA2 datasets revealed that both IGF2BP2 and Nrf2 are significantly up‐regulated in COAD, and elevated expression levels of these genes are associated with reduced DFS (Figures S1A,B). These findings were substantiated by increased levels of H3K18la, IGF2BP2 and Nrf2 expression in clinical CRC tissues (Figure 1B). We also analysed data from TCGA database and found that IGF2BP2 mRNA expression was significantly higher in COAD tumour tissues (Figure S1C). More importantly, the data revealed a significant positive correlation between Nrf2 and IGF2BP2 mRNA expression, while no such correlation was observed with IGF2BP1 or IGF2BP3 (Figure S1D–F), providing further evidence for their interconnected roles in CRC progression. Kaplan–Meier analysis demonstrated that showed a significantly shorter overall survival in patients with CRC exhibiting elevated levels of lactylation, H3K18la, IGF2BP2 and Nrf2 levels correlate with poor overall survival in CRC patients (Figure 1C). Interestingly, stratifying by tumour stage revealed a stepwise increase in the levels of lactylation, H3K18la, IGF2BP2 and Nrf2 with increasing tumour burden (Figure 1D), suggesting a strong association between the lactate–IGF2BP2–Nrf2 axis and disease progression. TSA‐amplified multiplexed immunofluorescence further confirmed the elevated expression of H3K18la, IGF2BP2, Nrf2 and the ferroptosis suppressor GPX4 in CRC tissues (Figure 1E). To establish the biological relationships between these molecules, we performed correlation analyses within our CRC patient cohort. The results confirmed significant positive relationships between: H3K18la and IGF2BP2; IGF2BP2 and Nrf2; and Nrf2 and GPX4 (Figure 1E). These data suggested a potential link between lactylation and the ferroptosis‐resistant phenotype in CRC.

To investigate this relationship mechanistically, we measured the intracellular lactylation and H3K18la levels across a panel of CRC cell lines and observed their up‐regulation (Figure 1F,G), prompting us to examine the effects of lactate supplementation. Notably, their up‐regulation was most pronounced in SW480 and Caco‐2 cells, which were consequently chosen for further experiments. We observed a reduction in CRC cell viability and proliferation after treatment with the ferroptosis inducer RSL3, whereas lactate treatment reversed this effect (Figure S2A,B). Moreover, the reversal was progressively enhanced with increasing lactate concentrations (Figure S2A,B). RSL3 treatment depleted GSH reserves while elevating lipid peroxidation markers (lipid ROS and MDA), and Fe^2^⁺, effects that were dose‐dependently attenuated by lactate co‐treatment (Figure S2C). These findings indicated that lactate confers a concentration‐dependent resistance to RSL3‐induced ferroptosis in CRC cells. To confirm that RSL3 specifically induces ferroptosis, we performed rescue experiments using pathway‐specific cell death inhibitors. Only the ferroptosis inhibitor Fer‐1 rescued RSL3‐induced cell death, while inhibitors of apoptosis (Z‐VAD‐FMK), necrosis (Nec‐1) and pyroptosis (VX‐765) were ineffective (Figure S3A). Consistently, Fer‐1 attenuated RSL3‐induced mitochondrial damage and lipid peroxidation (Figure S3B,C). Furthermore, Fer‐1 restored the levels of GSH, MDA and Fe^2^⁺ (Figure S3D–F). These data unequivocally demonstrated that RSL3‐triggered cell death is ferroptosis‐specific. Importantly, H3K18la, IGF2BP2 and Nrf2 levels remained unchanged in cells treated with RSL3. However, lactate treatment induced a dose‐dependent up‐regulation of all three factors (Figure S2D), suggesting a mechanistic link between H3K18la and the activation of a Nrf2‐mediated ferroptosis defence pathway.

### 3.2 Lactate promoted Nrf2‐mediated ferroptosis resistance in CRC cells and M2 macrophage polarisation

**FIGURE 2:**
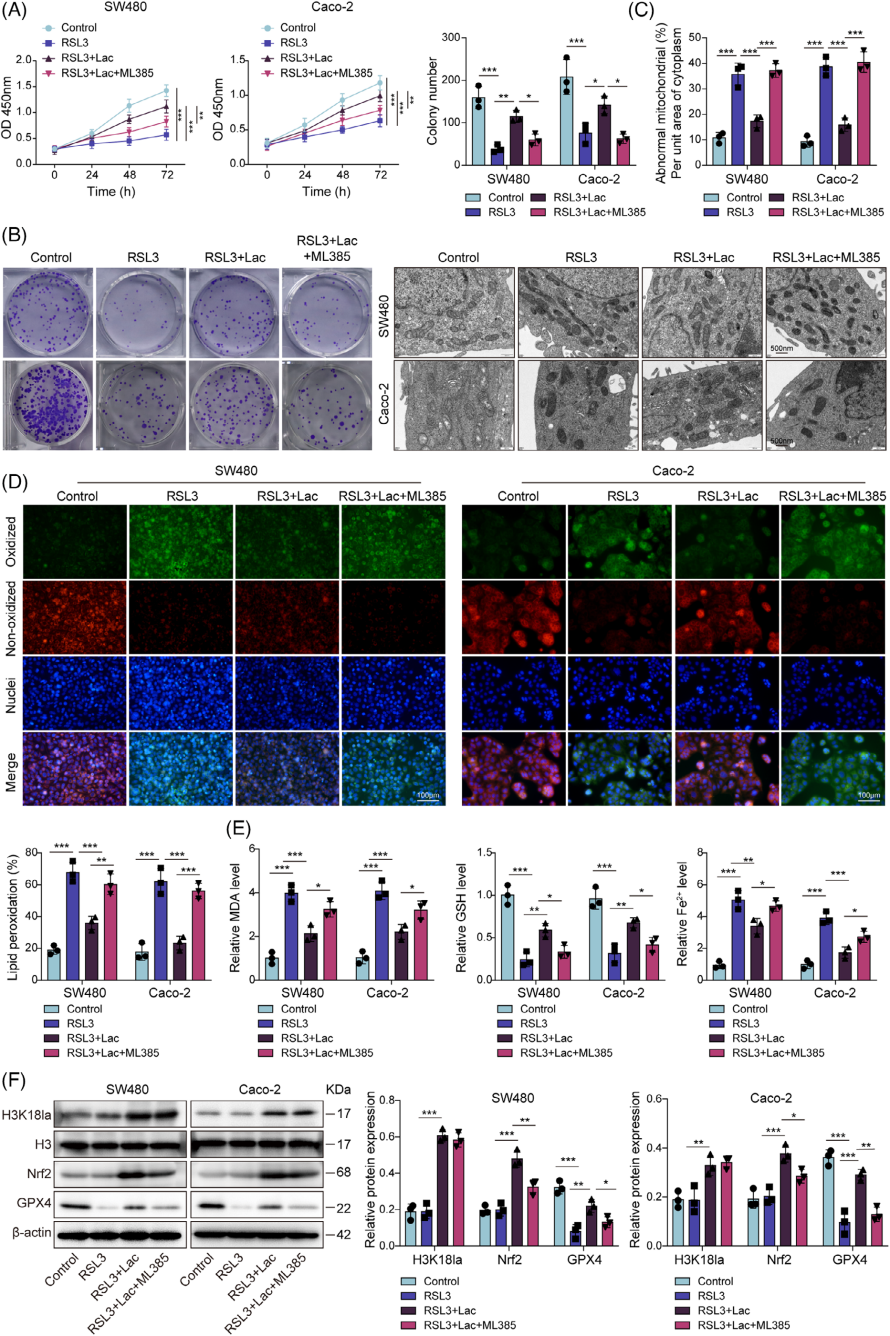
Lactate promoted Nrf2‐mediated ferroptosis resistance in CRC cells. SW480 and Caco‐2 cells were treated with lactate (10 mM) and the Nrf2 inhibitor ML385 (5 µM) for 24 h, followed by treatment with RSL3. Experimental groups: control, RSL3, Lac+RSL3, Lac+ RSL3+ML385. (A) Cell viability was measured by CCK‐8 assay. (B) Colony formation assay was performed to evaluate cell proliferation. (C) Representative transmission electron microscopy (TEM) images of mitochondrial ultrastructure in different groups (scale bar = 500 nm). (D) Representative immunofluorescence images of cells stained with C11‐BODIPY (oxidised form, green) and DAPI (nuclei, blue) following treatments as indicated. Scale bar = 100 µm. (E) MDA, Fe^2+^ and GSH levels were measured using commercial kits. (F) H3K18la, Nrf2 and GPX4 levels were examined using western blot. Measurement data are presented as mean ± SD. *n* = 3. *p < .05, **p < .01, ***p < .001.

Given the lactate‐induced up‐regulation of Nrf2, we next investigated whether the ferroptosis‐resistant phenotype was dependent on Nrf2 activity. We observed that Nrf2 inhibitor ML385 abrogates the protective effects of lactate supplementation against RSL3‐induced ferroptosis in CRC cells, evidenced by restoring decreases in cell viability (Figure 2A) and clonogenic survival (Figure 2B). TEM revealed that RSL3 treatment induces characteristic ferroptotic mitochondrial alterations, including shrinkage, increased membrane density and cristae reduction. These changes were markedly attenuated by lactate supplementation, whereas co‐treatment with ML385 restored the ferroptotic phenotype (Figure 2C). Consistent with these morphological findings, C11‐BODIPY staining showed elevated lipid peroxidation in RSL3‐treated cells, which was suppressed by lactate and re‐induced by ML385 (Figure 2D). Additionally, the up‐regulation of GSH (Figure 2E), H3K18la, Nrf2 and GPX4 (Figure 2F) and the reduction of MDA and Fe^2^⁺ (Figure 2E) induced by lactate treatment in RSL3‐induced cells were blocked by treating with ML385 (except for H3K18la) (Figure 2E,F), indicating that lactate confreres ferroptosis resistance through a Nrf2‐dependent mechanism. To further validate Nrf2 dependency, we performed genetic knockdown of Nrf2 using two independent shRNAs, which produced results consistent with those of ML385 treatment (Figure S4A–E), further confirming that lactate confers ferroptosis resistance through an Nrf2‐dependent mechanism. To confirm that our findings are not specific to RSL3, we performed parallel experiments using erastin, a well‐established ferroptosis inducer. As shown in Figure S5A–D, the similar effects on cell viability, colony formation, lipid ROS, MDA, GSH, Fe^2+^ levels and the expression of Nrf2, and GPX4 exerted by erastin, which was reversed by lactate, confirming that lactate‐induced ferroptosis resistance is a general mechanism rather than an RSL3‐specific phenomenon.

Subsequently, the results suggested that lactate treatment dose‐dependently decreases mRNA levels of M1 (pro‐inflammatory) markers IL‐1β, IL‐12 and iNOS, while up‐regulating the M2 (anti‐inflammatory) markers IL‐10, Arg‐1 and Mrc‐1 (Figure S6A). Flow cytometry confirmed a lactate‐induced shift towards the M2 phenotype, with reduced M1 (CD11b+CD86+) and increased M2 (CD11b+CD206+) macrophages (Figure S6B). This was further substantiated by ELISA analysis showing decreased secretion of IL‐1β and IL‐12, coupled with increased IL‐10 upon lactate treatment (Figure S6C). Importantly, co‐treatment with ML385 attenuated lactate‐mediated skewing towards the M2 phenotype (Figure S6A–C), suggesting that this process is Nrf2 dependent. Collectively, in addition to cell ferroptosis resistance, lactate can promote CRC cell proliferation through the Nrf2‐mediated polarisation of macrophages towards the M2 phenotype.

### 3.3 IGF2BP2 promoted CRC cell ferroptosis resistance and M2 macrophage polarisation

**FIGURE 3:**
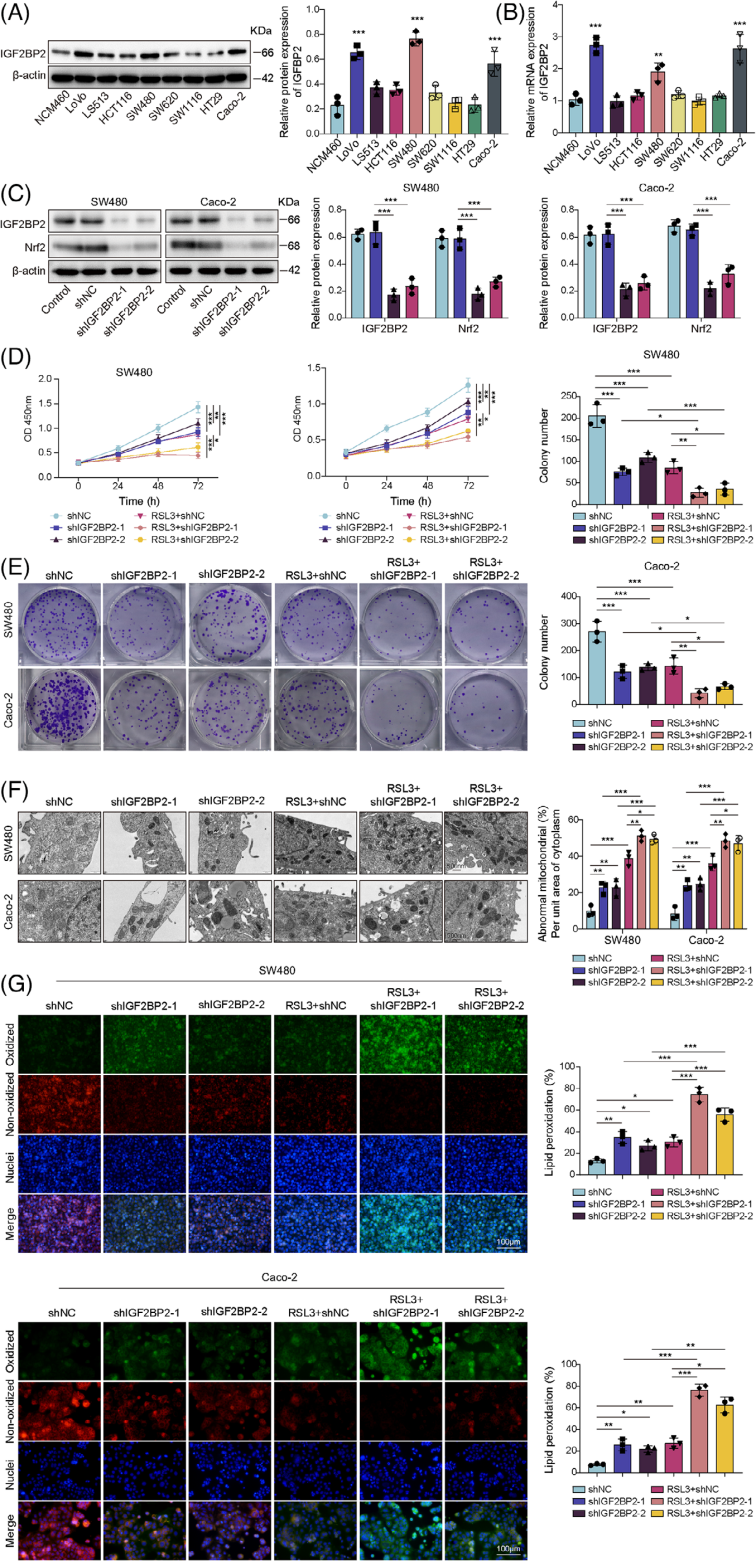
IGF2BP2 promoted CRC cell ferroptosis resistance. (A and B) Western blotting and RT‐qPCR analysis of IGF2BP2 expression in the normal colon epithelial cell line NCM460 and CRC cell lines (LoVo, LS513, HCT116, SW480, SW620, SW1116, HT29 and Caco‐2). (C) Western blot analysis of IGF2BP2 and Nrf2 levels in SW480 and Caco‐2 cells transfected with shIGF2BP2 (IGF2BP2‐KD). IGF2BP2‐KD cells were treated with RSL3 and group as: shNC, shIGF2BP2‐1, shIGF2BP2‐2, RSL3 + shNC, RSL3 + shIGF2BP2‐1 and RSL3 + shIGF2BP2‐2. (D) Cell viability was assessed by CCK‐8 assay. (E) Colony formation measured cell proliferative capacity. (F) Representative transmission electron microscopy (TEM) images of mitochondrial ultrastructure in different groups (scale bar = 500 nm). (G) Representative immunofluorescence images of cells stained with C11‐BODIPY and DAPI. Scale bar = 100 µm. Measurement data were presented as mean ± SD. *n* = 3. *p < .05, **p < .01, ***p < .001.

To identify potential RBPs that regulate Nrf2 mRNA stability, we performed bioinformatics analysis using the starBase and cisbp‐RNA databases. This analysis revealed seven overlapping factors that could bind to Nrf2 mRNA: HNRNPL, IGF2BP1, IGF2BP2, IGF2BP3, PABPC4, SRSF1 and SRSF9 (Figure S7A). Further expression analysis of COAD samples using the GEPIA2 database demonstrated that among these candidates, only IGF2BP2 was significantly up‐regulated in tumour tissues (Figure S7B). Based on these bioinformatic findings, we examined IGF2BP2 expression across CRC cell lines and observed an elevation in IGF2BP2 expression, peaking in SW480 and Caco‐2 cells (Figure 3A,B). IGF2BP2 knockdown in these two cell lines decreased Nrf2 levels (Figure 3C), suggesting a regulatory relationship between them. Moreover, IGF2BP2 knockdown alone or RSL3 treatment alone reduced cell viability (Figure 3D) and clonogenic survival (Figure 3E). Concurrently, TEM analysis revealed characteristic ferroptotic morphological changes, such as shrunken mitochondria with increased membrane density (Figure 3F). These treatments also increased lipid peroxidation (Figure 3G), up‐regulated MDA and Fe^2^⁺ levels (Figure S8A,B), depleted GSH (Figure S8C) and down‐regulated GPX4 expression (Figure S8D). Importantly, combined IGF2BP2 knockdown and RSL3 treatment further exacerbated these effects (Figures 3D–G and S8A–D), indicating that IGF2BP2 depletion sensitises CRC cells to RSL3‐induced ferroptosis by impairing the Nrf2–GPX4 antioxidant pathway. Notably, IGF2BP2 knockdown prevented their ability to skew macrophage polarisation towards an anti‐inflammatory M2 phenotype, evidenced by increased expression of M1 markers and decreased M2 markers (Figure S9A). Flow cytometry confirmed higher proportions of M1 (CD11b+CD86+) and reduced M2 (CD11b+CD206+) macrophages when co‐cultured with IGF2BP2‐depleted cells (Figure S9B). Consistent with these observations, ELISA revealed increased secretion of IL‐1β and IL‐12 and decreased IL‐10 after knocking down IGF2BP2 (Figure S9C). These results demonstrated that IGF2BP2 promotes both cell‐intrinsic ferroptosis resistance and M2 macrophage polarisation in CRC.

### 3.4 IGF2BP2 KO sensitised CRC cells to ferroptosis

**FIGURE 4:**
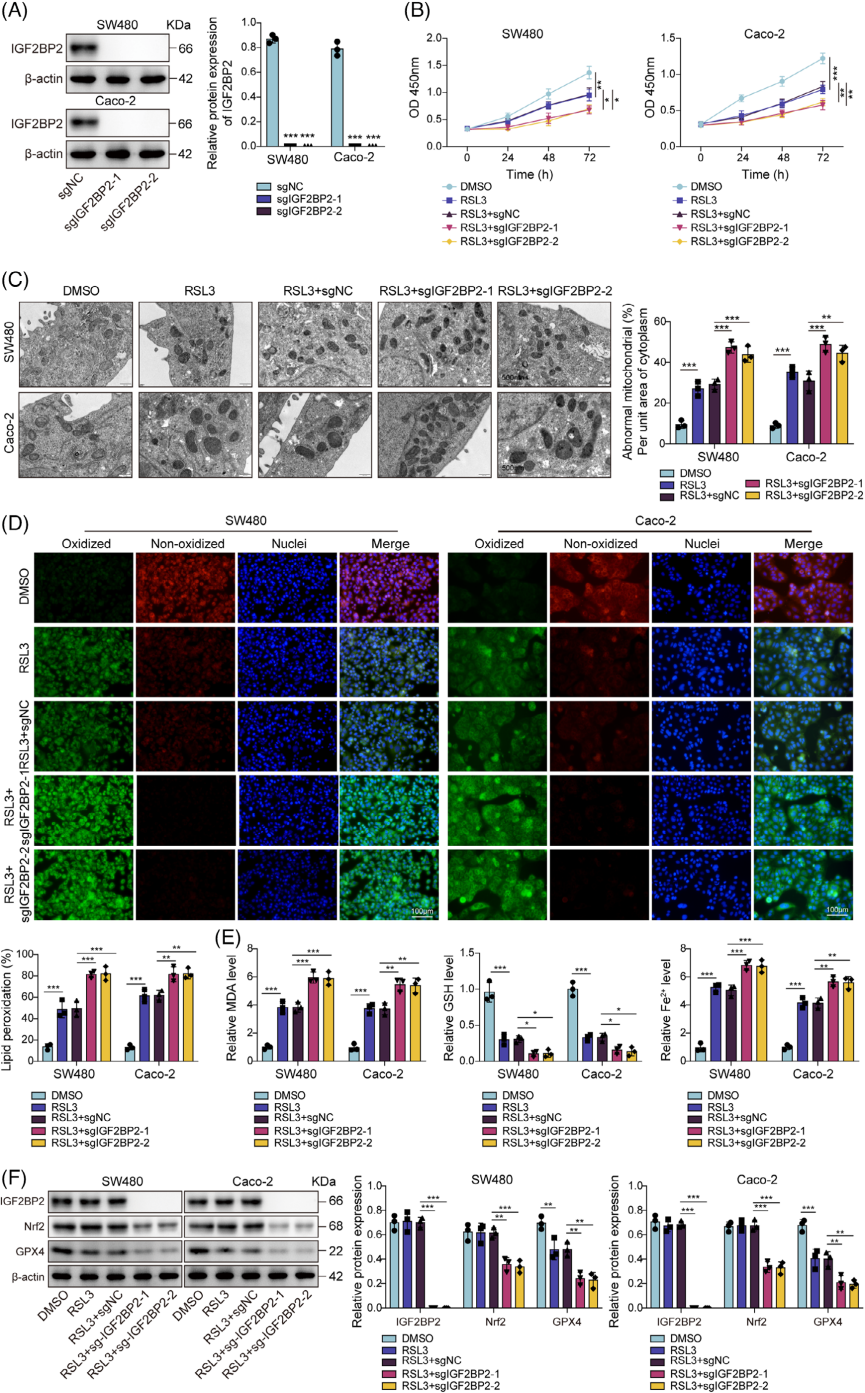
IGF2BP2 knockout sensitised CRC cells to ferroptosis. (A) Western blot validation of IGF2BP2 knockout (KO) in SW480 and Caco‐2 cells generated by CRISPR–Cas9 using two independent sgRNAs (sgIGF2BP2‐1, sgIGF2BP2‐2). IGF2BP2‐KO SW480/Caco‐2 cells were treated with RSL3 (or DMSO control) and group as: DMSO, RSL3, RSL3 + sgNC, RSL3 + sgIGF2BP2‐1 and RSL3 + sgIGF2BP2‐2. (B) Cell viability was measured by CCK‐8 assays. (C) Representative transmission electron microscopy (TEM) images showing mitochondrial morphology (scale bar = 500 nm). (D) Lipid peroxidation was assessed by C11‐BODIPY immunofluorescence. Scale bar = 100 µm. (E) Commercial kit quantification of intracellular MDA, Fe^2+^ and GSH levels. (F) Western blot analysis of IGF2BP2, Nrf2 and GPX4 expression. Measurement data were presented as mean ± SD. *n* = 3. *p < .05, **p < .01, ***p < .001.

To provide definitive evidence for the role of IGF2BP2, we established stable IGF2BP2 KO cell lines using CRISPR–Cas9. The results showed the complete absence of IGF2BP2 protein expression in the sgIGF2BP2‐1 and sgIGF2BP2‐2 clones (Figure 4A). Subsequently, we observed that IGF2BP2 KO exacerbates the reduction of cell viability induced by RSL3 (Figure 4B). This potent sensitisation to RSL3 was confirmed across all key ferroptosis markers. Specifically, the RSL3+sgIGF2BP2 groups displayed far more severe mitochondrial shrinkage and increased membrane density (Figure 4C). Similarly, IGF2BP2‐KO cells exhibited a dramatic increase in lipid peroxidation (Figure 4D), MDA and Fe^2+^ levels (Figure 4E), alongside more profound GSH depletion (Figure 4E). Moreover, while RSL3 treatment alone slightly reduced GPX4, the IGF2BP2‐KO cells displayed a reduction of Nrf2 and a further decrease in GPX4 expression after RSL3 treatment (Figure 4F). These findings suggested that IGF2BP2 KO sensitises CRC cells to ferroptosis.

### 3.5 IGF2BP2 was essential for lactate‐mediated ferroptosis resistance

**FIGURE 5:**
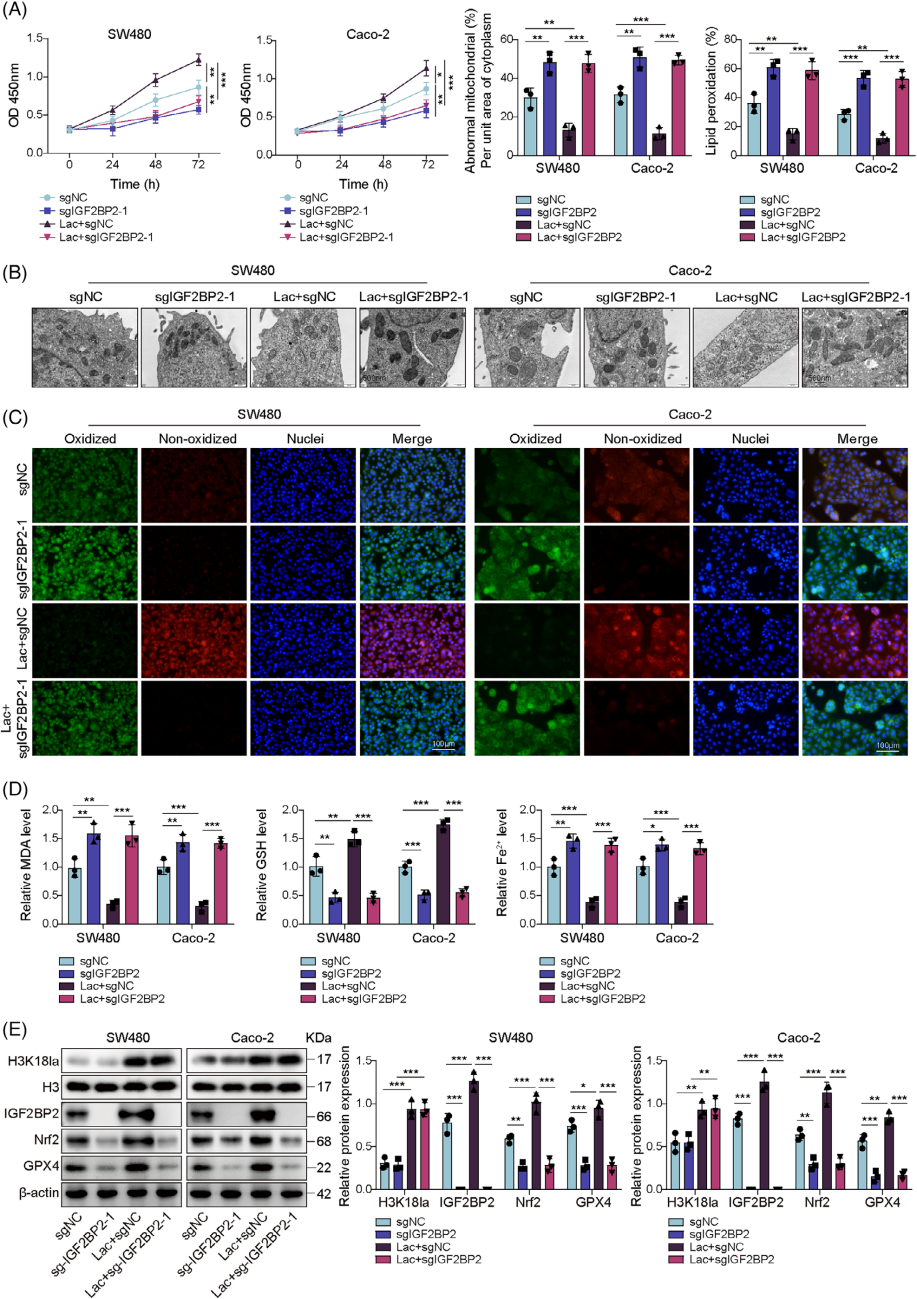
IGF2BP2 was essential for lactate‐mediated ferroptosis resistance. IGF2BP2‐KO SW480/Caco‐2 cells were treated with lactate in the presence of RSL3 to induce ferroptotic stress and group as: sgNC, sgIGF2BP2‐1, Lac + sgNC and Lac + sgIGF2BP2‐1. (A) Cell viability was measured by CCK‐8 assays. (B) Representative transmission electron microscopy (TEM) images showing mitochondrial morphology (scale bar = 500 nm). (C) Lipid peroxidation was assessed by C11‐BODIPY immunofluorescence. Scale bar = 100 µm. (D) Commercial kit quantification of intracellular MDA, Fe^2+^ and GSH levels. (E) Western blot analysis of H3K18la, IGF2BP2, Nrf2 and GPX4. Measurement data were presented as mean ± SD. *n* = 3. *p < .05, **p < .01, ***p < .001.

Next, we performed a rescue experiment to determine if IGF2BP2 is the required mediator for lactate's protective effects. The results indicated that in the control cells, the addition of lactate effectively rescues the cells from RSL3‐induced death, restoring cell viability (Figure 5A) and preventing the ferroptotic phenotype, as seen by normalised mitochondrial morphology (Figure 5B), reduced lipid peroxidation (Figure 5C) and normalised MDA, Fe^2+^ and GSH levels (Figure 5D). Crucially, this lactate‐mediated rescue was completely abrogated in the IGF2BP2‐KO cells. The Lac+sgIGF2BP2 group remained as sensitive to RSL3 as the KO cells without lactate, showing low viability (Figure 5A) and high levels of all ferroptosis markers (Figure 5B–D). Moreover, lactate still induced its upstream signal, H3K18la (which was unchanged) in IGF2BP2‐KO cells, but the downstream induction of Nrf2 and GPX4 was completely blocked (Figure 5E). To further confirm that the ferroptosis resistance phenotype is specifically dependent on IGF2BP2, we also performed a pharmacological rescue experiment. First, we confirmed that stable overexpression of IGF2BP2 in SW480 and Caco‐2 cells led to a corresponding increase in Nrf2 protein levels (Figure S10A). We then subjected these cells to RSL3‐induced ferroptotic stress, with or without the specific IGF2BP2 inhibitor, CWI1‐2 (which targets the KH3‐4 domains). As expected, the IGF2BP2‐overexpressing cells showed significantly enhanced cell viability (Figure S10B), reduced mitochondrial damage (Figure S10C), lower lipid peroxidation (Figure S10D) and normalised ferroptosis markers (decreased MDA/Fe^2+^, increased GSH) (Figure S10E) compared with the control group. Notably, the addition of the inhibitor CWI1‐2 reversed all these protective effects (Figure S10B–E). Furthermore, CWI1‐2 treatment effectively blocked the ability of IGF2BP2 overexpression to maintain high Nrf2 and GPX4 levels (Figure S10F). Taken together, IGF2BP2 was the specific mediator of lactate‐induced ferroptosis resistance.

### 3.6 Lactate mediated the lactylation modification of IGF2BP2 protein

**FIGURE 6:**
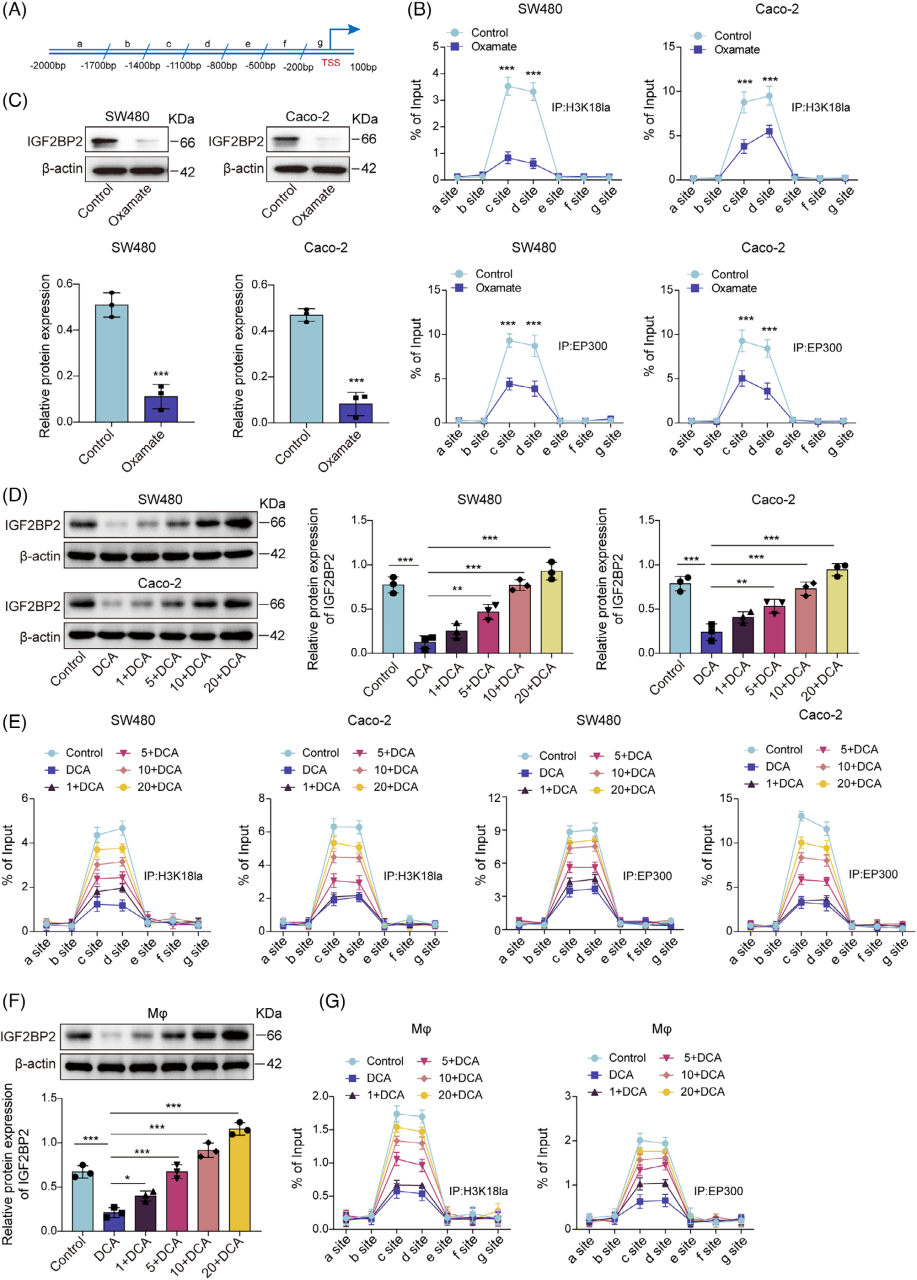
Lactate mediated the lactylation modification of IGF2BP2 protein. (A) Schematic representation of the igf2bp2 promoter region divided into segments a–g relative to the transcription start site (TSS), for analysis of H3K18la distribution. SW480 and Caco‐2 cells were treated with the H3K18la inhibitor oxamate. (B) ChIP–qPCR assays examined H3K18la and EP300 binding at the IGF2BP2 promoter. (C) Western blot analysis of IGF2BP2 expression. SW480 and Caco‐2 cells were pretreated with the glycolysis inhibitor DCA (20 mM) for 12 h, then stimulated with lactate (0, 1, 5, 10 and 20 mM) for 24 h, group as: control, DCA, Lac (1 mM) + DCA, Lac (5 mM) + DCA, Lac (10 mM) + DCA or Lac (20 mM) + DCA. (D) Western blot analysis of IGF2BP2 expression. (E) ChIP–qPCR of H3K18la and EP300 at the IGF2BP2 promoter. THP‐1 cells were differentiated into macrophages with PMA (200 nM) for 24 h, pretreated with DCA and stimulated with lactate (0, 1, 5, 10 and 20 mM) for 24 h. (F) Western blot analysis of IGF2BP2 expression in macrophages. (G) ChIP–qPCR of H3K18la and EP300 at the IGF2BP2 promoter in the macrophages. Measurement data are presented as mean ± SD. *n* = 3. **p < .01, ***p < .001.

Analysis of the IGF2BP2 promoter region revealed enrichment of H3K18la and acetyltransferase EP300 at segments c and d relative to the transcription start site (TSS) in SW480 and Caco‐2 cells (Figure 6A,B). We then performed a series of experiments to determine the functional necessity of EP300 and H3K18 lactylation in IGF2BP2 transcriptional regulation. The results indicated that treatment with the H3K18la inhibitor oxamate reduces this enrichment, concomitant with decreased IGF2BP2 expression (Figure 6B,C). Moreover, EP300 knockdown revealed a significant reduction in both EP300 and H3K18la enrichment at the IGF2BP2 promoter (Figure S11A,B). We also found that co‐transfection of HEK293T cells with H3–WT enhance IGF2BP2 promoter activity; however, when cells were transfected with the H3K18R–Mut, which cannot undergo lactylation at lysine 18, luciferase activity was significantly reduced compared with the H3–WT condition (Figure S11C). Consistent with these findings, silencing of EP300 resulted in a marked decrease in the mRNA and protein expression levels of IGF2BP2. A similar down‐regulation was also observed for Nrf2 (Figure S11D, E). These data suggested that EP300‐mediated H3K18 lactylation is necessary for IGF2BP2 transcriptional activation. To investigate whether lactate could directly mediate this modification, cells were pretreated with the glycolysis inhibitor DCA to deplete intracellular lactate, followed by exogenous lactate supplementation. DCA treatment reduced global protein lactylation (Pan Kla) and H3K18la levels in a dose‐dependent manner (Figure S12A, B). Moreover, DCA treatment decreased the IGF2BP2 levels, which were rescued by lactate treatment in a dose‐dependent manner (Figure 6D). The ChIP assay revealed that DCA diminished H3K18la and EP300 enrichment at IGF2BP2 promoter segments c and d, while lactate treatment increased their enrichment (Figure 6E). These lactate add‐back experiments confirmed that the effects of DCA are specifically due to lactate depletion. Similar results were observed in THP‐1 derived macrophages, where DCA decreased IGF2BP2 expression and H3K18la/EP300 promoter binding, which was reversed upon lactate treatment in a dose‐dependent manner (Figure 6F,G). Parallel experiments using AZ‐33, another glycolytic inhibitor, produced comparable results (Figure S13A–D), confirming the specificity of this mechanism. Furthermore, we investigated the potential involvement of the lactate receptor GPR81, as histone modification and receptor signalling are two major pathways for lactate function.^36^ Treatment with 3‐OBA, a specific inhibitor of GPR81 did not affect the basal levels of H3K18la, IGF2BP2 or Nrf2. However, it significantly attenuated the lactate‐induced up‐regulation of all three markers (Figure S13E). Consistent with this pharmacological inhibition, GPR81 knockdown also substantially blunted the ability of lactate to increase H3K18la, IGF2BP2 and Nrf2 expression (Figure S13F). These findings suggested that lactate promotes H3K18la modification in a manner dependent on GPR81 signalling. Taken together, lactate directly modulated IGF2BP2 transcription by recruiting EP300 to IGF2BP2 promoter region and inducing the H3K18la modification.

### 3.7 Lactate‐mediated IGF2BP2 enhanced the stabilisation of Nrf2 mRNA

**FIGURE 7:**
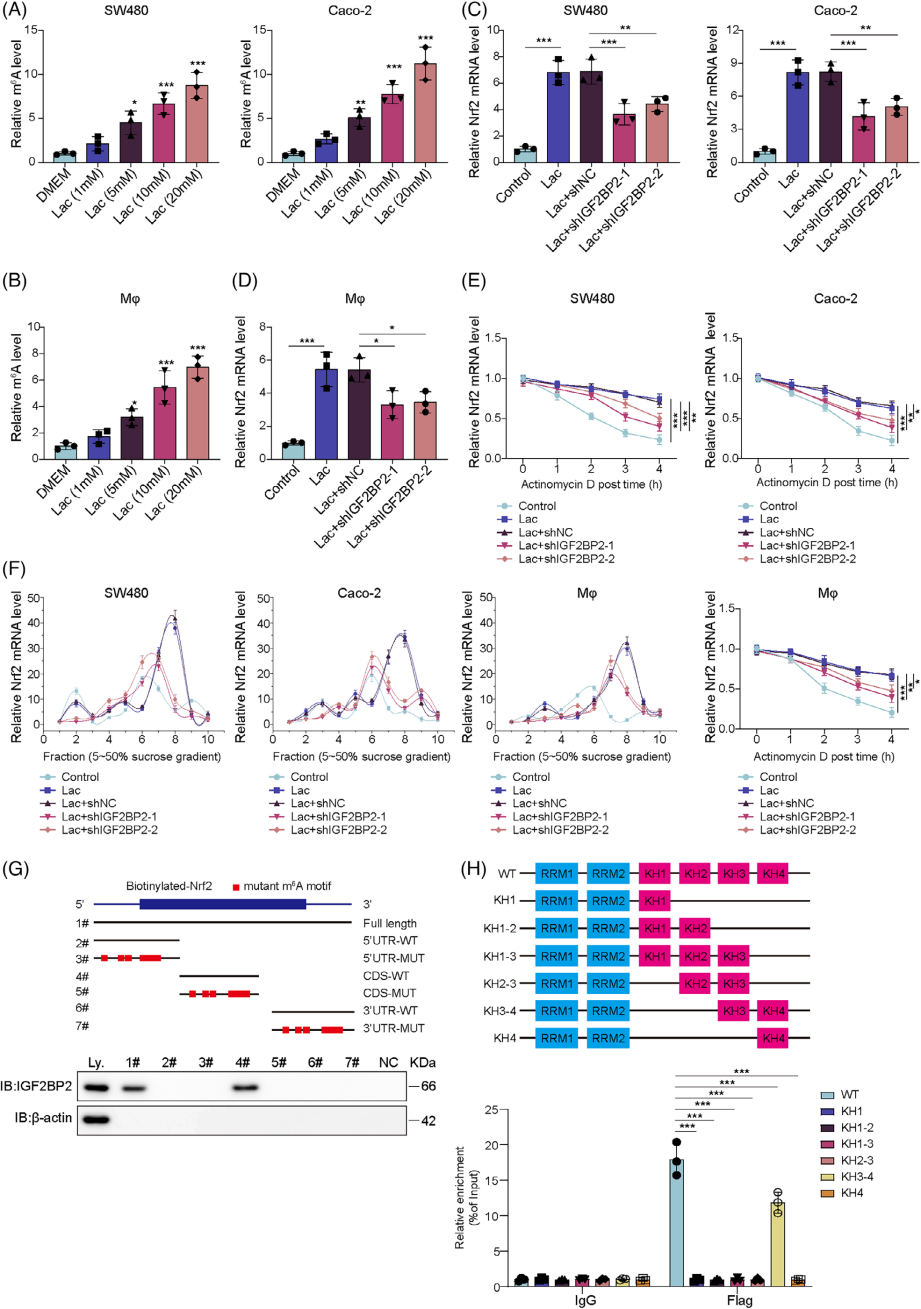
Lactate‐mediated IGF2BP2 enhanced the stabilisation of Nrf2 mRNA. (A) SW480 and Caco‐2 cells were treated with lactate (0, 1, 5, 10 or 20 mM) for 24 h. MeRIP–qPCR analysis of m^6^A modification in Nrf2 mRNA. (B) THP‐1 cells were differentiated into macrophages and treated with lactate (0, 1, 5, 10 and 20 mM). MeRIP–qPCR analysis of m^6^A modification on Nrf2 mRNA. (C) Cells stimulated with 10 mM lactate were then transfected with shIGF2BP2‐1 or shIGF2BP2‐2. RT‐qPCR analysis of the Nrf2 mRNA levels. (D) Macrophages stimulated with 10 mM lactate were transfected with shIGF2BP2‐1 or shIGF2BP2‐2. RT‐qPCR analysis of the Nrf2 mRNA levels. (E) Nrf2 mRNA stability was assessed using an Actinomycin D chase assay. (F) Polysome profiling analysis of Nrf2 mRNA translation efficiency. Polysomes in cytoplasmic extracts from SW480, Caco‐2 cells and THP‐1 derived macrophages with 10 mM lactate and transfected with either shIGF2BP2‐1 or shIGF2BP2‐2 were fractionated by sucrose gradients, and the relative Nrf2 mRNA expression level in the gradient fractions was analysed using RT‒qPCR. (G) RNA pulldown assays using biotinylated Nrf2 mRNA fragments (3′UTR–WT, 5′UTR–WT, CDS–WT) or mutants (3′UTR–Mut, 5′UTR–Mut, CDS–Mut) to map IGF2BP2 binding sites. (H) RIP assays with Flag‐tagged truncated IGF2BP2 proteins (WT, KH1, KH1‐2, KH1‐3, KH2‐3, KH3‐4 and KH4) to identify Nrf2 mRNA‐binding domains. Measurement data are presented as mean ± SD. *n* = 3. *p < .05, **p < .01, ***p < .001.

The m^6^A modification is a reversible epitranscriptomic mark that modulates mRNA stability and translation. Given that Nrf2 expression was increased by both lactate and IGF2BP2 (Figures S2D and 3C), we next investigated whether this regulation occurred at the post‐transcriptional level via m^6^A modification. MeRIP–qPCR analysis revealed that lactate dose‐dependently increases m^6^A levels on Nrf2 mRNA in SW480 and Caco‐2 cells (Figure 7A) and THP‐1 derived macrophages (Figure 7B). Notably, the up‐regulation of Nrf2 mRNA was abrogated upon IGF2BP2 knockdown (Figure 7C and D), suggesting IGF2BP2 was required for the lactate‐induced m^6^A modification of Nrf2 mRNA. To determine whether lactate treatment affects Nrf2 mRNA stability, we performed actinomycin D chase assays and found that lactate supplementation prolonged the half‐life of Nrf2 mRNA, whereas IGF2BP2 knockdown reversed this lactate‐induced stabilisation (Figure 7E). Furthermore, lactate treatment increased the association of Nrf2 mRNA with polysome fractions, indicating enhanced translation efficiency. This effect was relieved upon IGF2BP2 depletion, with decreased polysome peaks (Figure 7F), demonstrating that IGF2BP2 is required for the lactate‐induced enhancement of Nrf2 translation. To map the IGF2BP2 binding sites, RNA pull‐down assays using biotinylated Nrf2 mRNA fragments revealed specific interactions with the CDS region (Figure 7G). Further analysis using truncated IGF2BP2 identified the KH3‐4 domains as critical for Nrf2 mRNA binding (Figure 7H). Moreover, to identify the specific binding sites of IGF2BP2 on Nrf2 mRNA, we performed computational and experimental analyses. Using the RM2Target (http://rm2target.canceromics.org/#/home) and deepSRAMP (http://www.cuilab.cn/sramp/) databases, we identified an overlapping IGF2BP2 binding motif and a predicted m^6^A site at position 2109 (AAACT) in the Nrf2 CDS (Figure S14A, B). Dual‐luciferase reporter assays using WT or Mut (A to T) Nrf2 constructs revealed that IGF2BP2 knockdown significantly reduce luciferase activity only in WT‐transfected HEK293T cells, confirming that IGF2BP2 binding stabilises Nrf2 mRNA via this specific site (Figure S14C). Moreover, we constructed a Mut IGF2BP2 plasmid lacking the critical KH3‐4 RNA‐binding domains (IGF2BP2–Mut) and transfected it into SW480 and Caco‐2 cells. Our results demonstrate that while the WT protein successfully maintained Nrf2 mRNA levels, expression of the KH3‐4 deletion Mut resulted in a significant decrease in Nrf2 mRNA (Figure S14D). These findings suggested that IGF2BP2 stabilises Nrf2 mRNA by binding to the m^6^A‐modified site at position 2109 via its KH3‐4 domains. Taken together, lactate up‐regulated IGF2BP2, which in turn bound to and enhanced m^6^A modification of Nrf2 mRNA within its coding region, potentially increasing its stability and translation in CRC cells.

### 3.8 Lactate promoted CRC cell ferroptosis resistance by regulating the IGF2BP2–Nrf2 axis

To directly evaluate the function of the IGF2BP2–Nrf2 axis in lactate‐mediated ferroptosis resistance, SW480 and Caco‐2 cells were pretreated with lactate, followed by RSL3 treatment in combination with IGF2BP2 knockdown or/and Nrf2 overexpression. Consistent with the results shown in Figures 3 and S8, IGF2BP2 knockdown further promoted ferroptosis sensitivity in RSL3‐induced CRC cells. Conversely, ectopic Nrf2 overexpression restored cell viability and proliferation (Figure S15A, B), reduced MDA, Fe^2^⁺ and lipid ROS levels, increased GSH (Figure S15C), Nrf2 and GPX4 expression (Figure S15D). Notably, Nrf2 overexpression had no effect on H3K18la or IGF2BP2 levels (Figure S15D). Importantly, these changes caused by Nrf2 overexpression were negated when combined with IGF2BP2 depletion, indicating that Nrf2 acts as downstream of IGF2BP2 in this context. In conclusion, lactate conferred ferroptosis resistance in CRC cells through IGF2BP2‐dependent up‐regulation of the Nrf2 antioxidant pathway.

### 3.9 Lactate promoted CRC cell M2 macrophage polarisation by regulating the IGF2BP2–Nrf2 axis

Co‐culture with lactate‐pre‐treated macrophages promoted CRC cell aggressiveness in a dose‐dependent manner. Specifically, lactate‐polarised macrophages significantly enhanced the proliferation, colony formation, migration and invasion of SW480 and Caco‐2 cells through paracrine mechanisms, with the strongest effects observed at 20 mM lactate. These findings provide functional evidence that lactate‐induced M2 macrophage polarisation drives CRC progression (Figure S16A–D). To delineate the role of the IGF2BP2–Nrf2 axis in regulating macrophage polarisation, THP‐1 monocytes were differentiated into macrophages and subjected to IGF2BP2 knockdown or/and Nrf2 overexpression. Western blot confirmed the efficient modulation of IGF2BP2 and Nrf2 levels (Figure S17A). In contrast to the effects of IGF2BP2 knockdown, Nrf2 overexpression resulted in decreased expression of M1 markers and elevated M2 markers (Figure S17B). Consistently, we observed reduced proportions of M1 and increased M2 macrophages (Figure S17C). ELISA further substantiated these findings, with decreased secretion of IL‐1β and IL‐12 and elevated IL‐10 levels (Figure S17D). Crucially, these effects were reversed by concomitant IGF2BP2 knockdown (Figure S17A–D), indicating that Nrf2 functions as downstream of IGF2BP2 tin driving M2 macrophage polarisation. Collectively, lactate–IGF2BP2 axis regulated macrophage plasticity through modulation of Nrf2 levels.

### 3.10 Lactate promoted CRC progression and M2 macrophage polarisation via the H3K18la–IGF2BP2–Nrf2 axis to confer ferroptosis resistance

**FIGURE 8:**
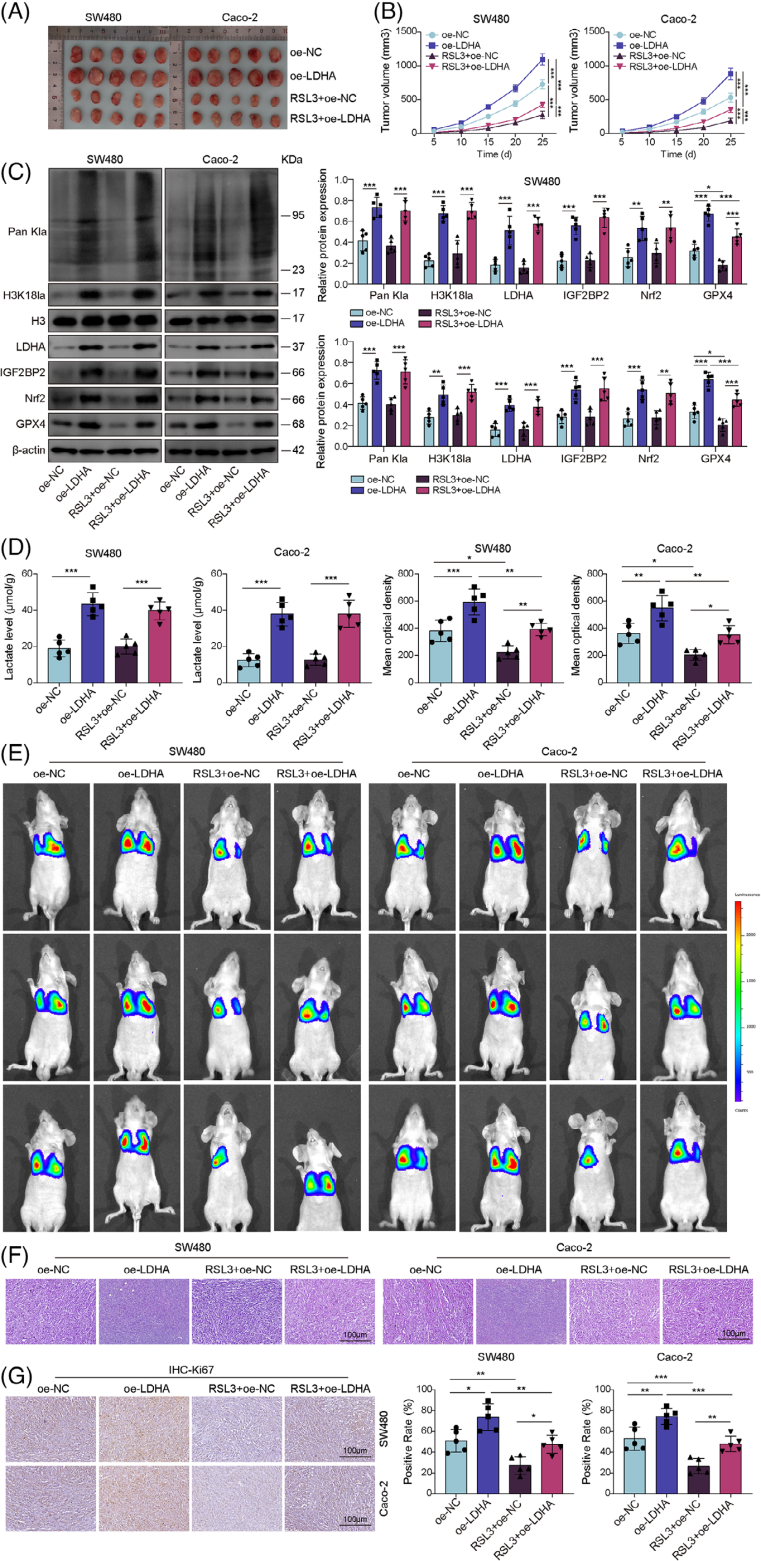
Lactate accelerated CRC cell proliferation and metastasis in mice by promoting Nrf2‐mediated ferroptosis resistance. Xenograft tumour growth in nude mice injected subcutaneously with SW480 and Caco‐2 cells stably overexpressing LDHA. Mice were then treated with or without RSL3 (intraperitoneal injection) and divided into four groups: OE‐NC, OE‐LDHA, RSL3 + OE‐NC and RSL3 + OE‐LDHA. (A and B) Tumour size and volume were monitored every 5 days. (C) Western blot analysis of pan‐Kla, H3K18la, LDHA, IGF2BP2, Nrf2 and GPX4 expression in tumour tissues from each group. (D) Lactate levels in tumour tissues were measured using a lactate assay kit. For the metastasis model, LDHA‐overexpressing CRC cells were injected intravenously into nude mice. Mice were then treated with or without RSL3 (intraperitoneal injection) and divided into four groups: OE‐NC, OE‐LDHA, RSL3 + OE‐NC and RSL3 + OE‐LDHA. (E) Representative bioluminescent images showed lung metastasis in nude mice. (F) H&E staining of lung tissue sections (scale bar = 100 µm). (G) IHC analysis of the proliferation marker Ki67 in metastatic nodules (scale bar = 100 µm). Measurement data are presented as mean ± SD. *n* = 5. *p < .05, **p < .01, ***p < .001.

To genetically model the sustained intratumoural lactate‐rich microenvironment in vivo, we established CRC cells stably overexpressing LDHA (Figure S18A). Subcutaneous injection of these LDHA‐overexpressing SW480 and Caco‐2 cells into nude mice significantly promoted tumour growth, as evidenced by increased tumour size and volume (Figures 8A,B and S18B,C), and elevated intratumoural lactate levels (Figures 8D and S18D). Concordantly, tumours from the LDHA group exhibited up‐regulated protein levels of Pan‐Kla, H3K18la, IGF2BP2, Nrf2 and GPX4 (Figure 8C). Treatment with RSL3 effectively suppressed tumour growth in control tumours; however, its efficacy was significantly attenuated in LDHA‐overexpressing tumours (Figure 8A,B). Notably, RSL3 treatment reduced GPX4 expression but did not affect the levels of lactate, Pan‐Kla, H3K18la, LDHA, IGF2BP2 or Nrf2 (Figure 8C,D), indicating that GPX4 acts downstream of this lactate‐driven signalling axis. We next investigated whether lactate production reshapes the tumour immune microenvironment. Flow cytometry analysis revealed that LDHA overexpression alters macrophage polarisation, reducing M1 macrophages while increasing M2 macrophages (Figure S18E). IHC analysis further confirmed an M2‐biased tumour microenvironment, showing increased expression of CD206 and Arg‐1 alongside decreased CD86 and iNOS expression in LDHA‐overexpressing tumours (Figure S18F). These tumours also exhibited enhanced proliferative activity, as indicated by elevated Ki67 staining (Figure S18F). In the experimental metastasis model, LDHA overexpression enhanced lung metastatic burden, as shown by increased bioluminescent signal (Figure 8E), more metastatic nodules (Figure 8F) and higher proliferative activity (Figure 8G). RSL3 treatment markedly suppressed metastasis in control mice and, importantly, reversed the pro‐metastatic effect conferred by LDHA overexpression (Figure 8E–G). Collectively, these in vivo data from a genetic lactate‐overproduction model demonstrate that sustained lactate generation drives CRC proliferation and metastasis by activating the H3K18la–IGF2BP2–Nrf2 axis to confer resistance to ferroptosis.

### 3.11 DCA inhibited CRC cell proliferation and metastasis in mice by repressing ferroptosis resistance

**FIGURE 9:**
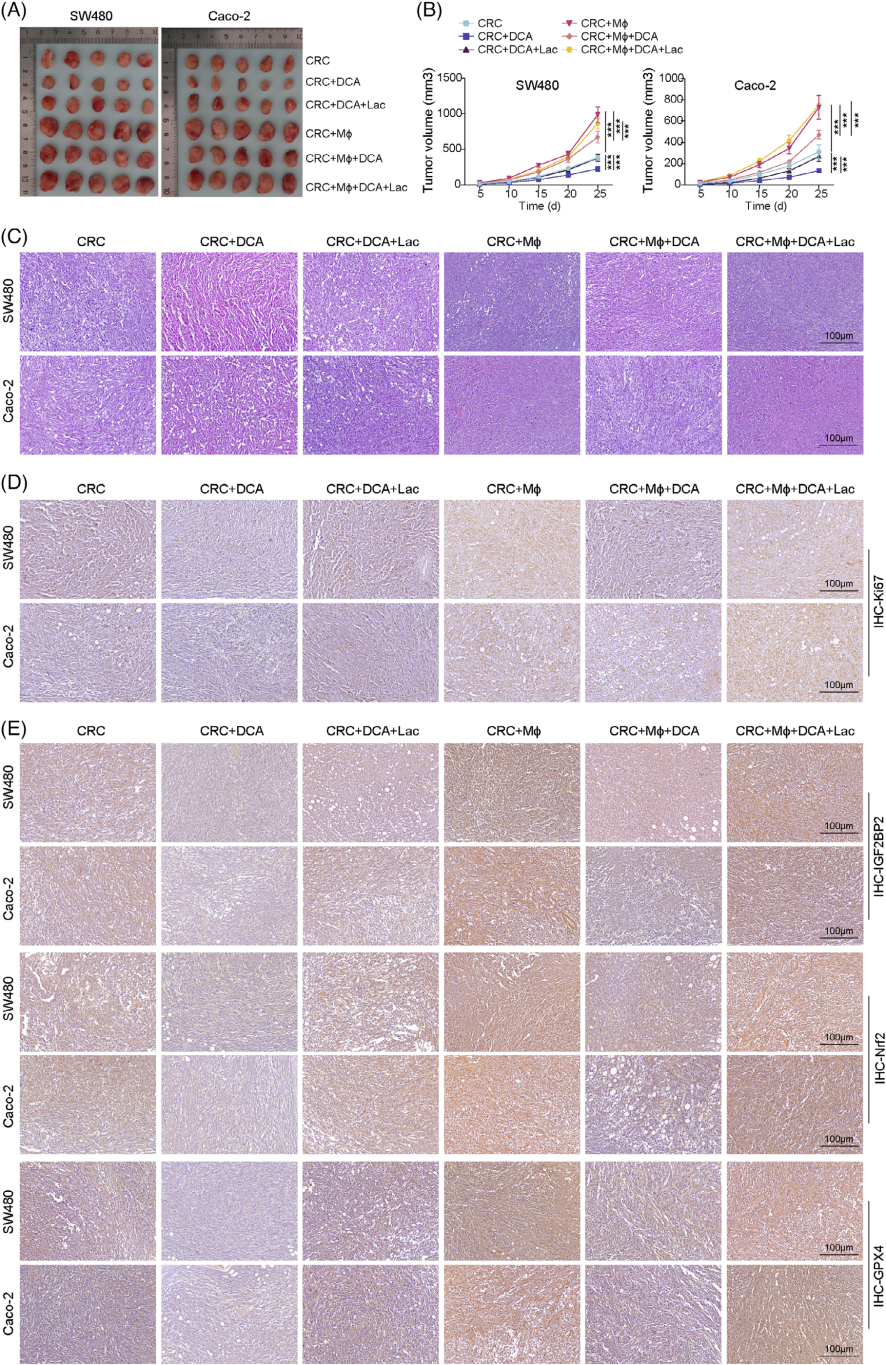
DCA inhibited CRC cell proliferation and metastasis in mice by repressing ferroptosis resistance. SW480 and Caco‐2 cells were co‐injected with or without THP‐1‐derived macrophages (MΦ) subcutaneously into nude mice pre‐treated with clodronate liposomes to deplete endogenous macrophages. Treatment groups: CRC (control), CRC + DCA, CRC + DCA + lactate, CRC + MΦ, CRC + MΦ + DCA and CRC + MΦ + DCA + lactate. DCA (dichloroacetate; 50 mg/kg) was administered intraperitoneally every other day. (A and B) Tumour size and volume were monitored every 5 days. (C and D) Histological analysis of H&E staining and Ki67 immunohistochemistry of tumour sections (scale bar = 100 µm). (E) IHC analysis of IGF2BP2, Nrf2 and GPX4 expression in tumour tissues. Measurement data are presented as mean ± SD. *n* = 5. ***p < .001.

To evaluate the effects of inhibiting lactate‐induced ferroptosis resistance in vivo, CRC cells were pretreated with DCA to deplete intracellular lactate levels. THP‐1 derived macrophages were similarly treated with DCA before subcutaneous co‐injection with CRC cells into nude mice pre‐cleared with endogenous macrophages using clodronate liposomes. Once the tumours were established, the mice received intraperitoneal RSL3 injections. The results indicated that DCA pretreatment significantly reduces tumour growth and volume (Figure 9A,B). Importantly, lactate supplementation partially reversed the tumour‐suppressive effects of DCA, confirming that the therapeutic efficacy is specifically mediated through lactate depletion. Co‐injection of macrophages enhanced tumour growth compared with CRC cells alone, but this promoting effect was effectively counteracted by DCA treatment (Figure 9A,B). Histological analysis revealed that DCA treatment results in smaller, less cellular tumour lesions with reduced proliferative capacity. Macrophage co‐injection increased both tumour cellularity and proliferation, effects that were reversed by DCA treatment. Lactate supplementation partially restored the proliferative phenotype even in the presence of DCA (Figure 9C,D). Moreover, DCA treatment down‐regulated IGF2BP2, Nrf2 and GPX4 expression levels. Co‐injection with macrophages up‐regulated these ferroptosis resistance markers, but DCA treatment effectively reduced their expression regardless of macrophage presence, while lactate supplementation reversed the change (Figures 9E and S19A,B). Notably, DCA treatment decreased H3K18la levels with or without macrophage presence, while lactate supplementation maintained elevated H3K18la (Figure S19B). DCA treatment also reduced lactate concentrations in tumour tissues across all treatment groups. Macrophage co‐injection was associated with elevated tissue lactate levels, which were effectively reduced by DCA treatment. Exogenous lactate supplementation restored tissue lactate concentrations, validating our experimental approach (Figure S19C). Similar anti‐tumour effects were observed after AZ‐33 treatment in parallel experiments (Figure S20A–E). Taken together, pharmacological depletion of lactate using DCA could effectively inhibit CRC tumour growth by disrupting the lactate–H3K18la–IGF2BP2–Nrf2 signalling axis.

### 3.12 IGF2BP2 knockdown inhibited Nrf2‐mediated ferroptosis resistance, as well as CRC cell proliferation and metastasis in mice

**FIGURE 10:**
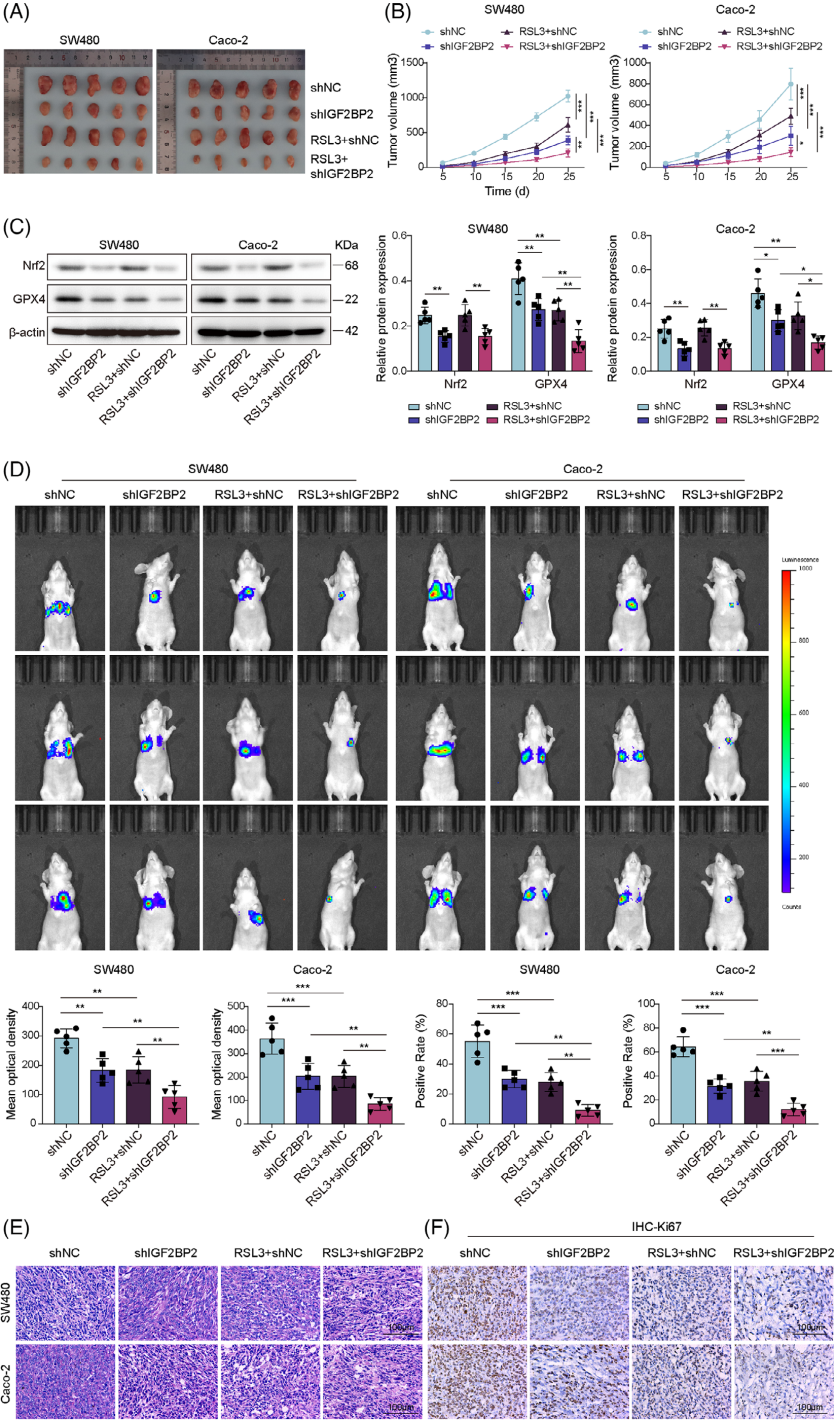
IGF2BP2 knockdown inhibited Nrf2‐mediated ferroptosis resistance, as well as CRC cell proliferation and metastasis in mice. Mice were subcutaneously injected IGF2BP2 knockdown (shIGF2BP2) CRC cells to generate xenograft tumours, followed by receiving intraperitoneal injections of RSL3 and group as: shNC, shIGF2BP2, RSL3 + shNC and RSL3 + shIGF2BP2. (A and B) The tumour size and volume were monitored every 5 days. (C) Western blot of tumour lysates to analyse Nrf2 and GPX4 levels. For the metastasis model, IGF2BP2 knockdown CRC cells were injected intravenously. (D) In vivo bioluminescence images of lung metastases. (E) H&E staining of lung tissue sections (scale bar = 100 µm). (F) IHC analysis of Ki67 expression in metastatic nodules (scale bar = 100 µm). Measurement data are presented as mean ± SD. *n* = 5. *p < .05, **p < .01, ***p < .001.

To directly evaluate the role of IGF2BP2 in ferroptosis resistance and tumour progression in vivo, IGF2BP2‐depleted SW480 and Caco‐2 cells were subcutaneously implanted into nude mice to generate xenograft tumours, followed by intraperitoneal injections of RSL3. We observed that IGF2BP2 knockdown alone attenuates tumour growth and reduced tumour volumes (Figure 10A,B). RSL3 treatment further suppressed tumour progression in shIGF2BP2 groups (Figure 10A,B). Subsequently, IGF2BP2 knockdown down‐regulated the Nrf2 and GPX4 levels (Figure 10C). As expected, a further reduction in GPX4 levels was observed in the RSL3+shIGF2BP2 group (Figure 10C). In addition, IGF2BP2 knockdown decreased the lung metastatic burden, as evidenced by the reduced bioluminescence signal and smaller metastatic nodules (Figure 10D,E). RSL3 treatment further diminished metastasis (Figure 10D,E). This was further confirmed by gross examination of the lung specimens, which revealed significantly fewer visible metastatic nodules in the shIGF2BP2 group. IGF2BP2 knockdown also reduced metastatic lesions to levels comparable to those of RSL3 treatment alone, whereas the combination of RSL3 treatment with IGF2BP2 depletion produced the most pronounced reduction in the metastatic burden (Figure S21). Immunohistochemical analysis of the metastatic lesions revealed decreased proliferation, as indicated by lower Ki67 staining, upon IGF2BP2 depletion or RSL3 treatment, with the combination exhibiting the greatest effect (Figure 10F). These findings suggested that targeting IGF2BP2 sensitises CRC cells to ferroptosis induced by RSL3, attenuating tumour growth, proliferation and metastatic dissemination.

## 4 DISCUSSION

CRC poses a significant global health burden with alarming incidence and mortality rates, highlighting the urgent need for innovative therapeutic strategies. Ferroptosis induction has gained increasing recognition as a viable strategy for anti‐cancer therapy. However, cancer cells can evolve various defence mechanisms against ferroptosis‐inducing stress, depending on their type, location and TME.^37^, ^38^ CRC displays inherent resistance to ferroptosis relative to other malignancies.^39^ Accumulating evidence has implicated the TME as a critical determinant of ferroptosis sensitivity,^40^ with TAMs playing a pivotal role in shaping the immunosuppressive and pro‐tumourigenic milieu.^41^ Notably, the polarisation of TAMs towards the M2 phenotype has been implicated in promoting ferroptosis resistance in cancer cells, thereby facilitating tumour growth.^42^ In this study, we unravelled a novel regulatory axis involving lactate, IGF2BP2 and Nrf2, which orchestrates ferroptosis resistance in CRC and promotes a pro‐tumourigenic TME through M2 macrophage polarisation.

The ability of lactylation modulates diverse biological processes, such as tumourigenesis, cancer progression, immune evasion and metabolic reprogramming.^43^, ^44^ Our study positioned lactate‐driven H3K18la as a key upstream regulator of the Nrf2‐mediated ferroptosis resistance pathway. This finding extends previous reports, which noted a general inhibition of CRC development under hypoxia when lactylation was blocked,^35^ by providing a specific mechanism. The role of Nrf2 as a master regulator of ferroptosis resistance is well‐established, primarily through its control over antioxidant gene expression and metabolic metabolism. For instance, deletion of Nrf2 results in apoferritin accumulation in the autophagosome, an elevated labile iron pool, and enhanced sensitivity to ferroptosis.^45^ In CRC, Nrf2–HO‐1 axis was indicated to counteract cetuximab's enhancement of RSL3‐induced ferroptosis, thereby promoting CRC progression.^24^ Consistently, our findings proved that lactate accelerates CRC cell growth both in vitro and in vivo by promoting Nrf2‐mediated ferroptosis resistance. Beyond this cell‐intrinsic mechanism, our findings highlighted that lactate‐driven H3K18la also potently shapes the extrinsic TME by fostering M2 macrophage polarisation. This aligns with previous findings that H3K18la promotes VCAM1 transcription and recruits M2 macrophages in gastric cancer.^46^ While our data emphasise lactylation as a key mechanism, we cannot exclude potential non‐lactylation pathways. Lactate may also modulate immunity through metabolic reprogramming of immune cells^47^ or via receptor‐mediated signalling such as through GPR81.^48^ These alternative pathways could complement lactylation to enhance immunosuppression. Collectively, our results highlighted lactate's multifaceted roles in regulating both ferroptosis sensitivity and macrophage polarisation, underscoring its value as a therapeutic target in CRC.

Subsequently, we found that IGF2BP2 promotes Nrf2‐mediated ferroptosis resistance in CRC and facilitates M2 macrophage polarisation. While IGF2BP2 is well‐established as an m^6^A reader protein with oncogenic roles in CRC,^49^ emerging evidence suggests its function extends beyond canonical m^6^A‐dependent mechanisms. For example, ALKBH5 knockdown increased Nrf2 expression in hypopharyngeal squamous cell carcinoma through an m^6^A–IGF2BP2‐dependent mechanism, thereby enhancing cancer cell ferroptosis resistance,^33^ consistent with our findings that IGF2BP2 depletion sensitises CRC cells to ferroptosis and impedes tumour progression. Notably, recent studies reveal that IGF2BP2 can regulate ferroptosis sensitivity through m^6^A‐independent mechanisms by forming RNA–protein complexes that stabilise key transcripts.^50^ In hepatocellular carcinoma, exosomal circUPF2 was found to enhance sorafenib resistance by forming a ternary complex with IGF2BP2 and SLC7A11 mRNA, stabilising SLC7A11 and suppressing ferroptosis independent of m^6^A modification,^51^ suggesting IGF2BP2 may employ both m^6^A‐dependent and ‐independent mechanisms to regulate ferroptosis, depending on cellular context and binding partners. In parallel to its cell‐intrinsic role, our study shows IGF2BP2 is also pivotal in shaping the immunosuppressive TME. This is consistent with reports in other cancers. In ovarian cancer, circITGB6 directly interacted with IGF2BP2 and FGF9 mRNA, thereby stabilising FGF9 mRNA and inducing polarisation of TAMs towards M2 phenotype.^52^ Zhang et al. suggested that circASPH enhances exosomal STING by interacting with IGF2BP2, facilitating M2 macrophage polarisation and accelerating CRC progression.^53^ Our results confirming IGF2BP2's role in M2 polarisation suggested it may serve as a central node integrating various regulatory RNAs to remodel the TME. Collectively, these findings unveiled a previously unrecognised role for IGF2BP2 in regulating ferroptosis sensitivity and immune microenvironment remodelling in CRC, expanding our understanding of its multifaceted functions in cancer malignancy through both m^6^A‐dependent and independent mechanisms.

Our mechanistic investigations reveal that lactate drives the lactylation of IGFF2BP2, a novel post‐translational modification that expands its functional repertoire in cancer progression. Numerous studies have demonstrated the involvement of lactylated proteins in various aspects of cancer initiation and progression. For instance, lactylation of METTL16 enhances its m^6^A modification activity on FDX1 mRNA, promoting cuproptosis in gastric cancer,^54^ while in hepatic stellate cells, IGF2BP2 knockdown has demonstrated to reduce both global lactylation and H3K18la levels,^55^ suggesting IGF2BP2 may both undergo and potentially influence lactylation dynamics. Specifically, we observed that lactate induces IGF2BP2 lactylation in both CRC cells and macrophages, enabling lactylated IGF2BP2 to bind and stabilise Nrf2 mRNA, thereby enhancing Nrf2 protein expression and ferroptosis resistance. This mechanism operates alongside other lactylation‐mediated immunosuppressive pathways, such as lactylation‐mediated M1‐to‐M2 macrophage polarisation,^56^ and lactylation‐driven METTL3 up‐regulation that enhances the immunosuppressive capacity of tumour‐infiltrating myeloid cells.^57^ Together, these mechanisms create a coordinated immunosuppressive and ferroptosis‐resistant microenvironment. Therapeutically, targeting the lactate–IGF2BP2–Nrf2 axis presents a promising strategy for CRC treatment. Existing LDHA inhibitors (FX‐11, GSK2837808A) could suppress lactate production at its source, while Nrf2 inhibitors (ML385; Brusatol) could directly counteract downstream signalling. Patients with hyperglycolytic CRC subtypes (CMS3), KRAS or PIK3CA mutations, or tumours featuring M2 macrophage infiltration would likely benefit most from such strategies. Combining these agents with standard ferroptosis inducers (sulfasalazine, sorafenib) or immunotherapies could synergistically overcome therapy resistance by simultaneously targeting cancer cell‐intrinsic defence mechanisms and the immunosuppressive TME. Taken together, our work unveiled a previously unrecognised regulatory axis where lactate‐induced IGF2BP2 lactylation stabilises Nrf2 mRNA to confer ferroptosis resistance and promote immunosuppression. This axis simultaneously promotes two critical pro‐tumourigenic phenotypes in CRC. Interestingly, these two phenotypes are not merely independent endpoints but are functionally intertwined within the complex TME, engaging in a reciprocal, positive feedback loop. This concept is supported by emerging evidence; for instance, M2‐polarised macrophages can deliver ferritin heavy chain 1 to colon cancer cells via exosomes, which sequesters intracellular free iron and thereby confers ferroptosis resistance,^58^ and conversely, ferroptosis‐stressed cancer cells can release factors such as oncogenic KRAS protein, which are then taken up by macrophages to drive M2 polarisation via STAT3 signalling.^59^ Therefore, we propose that our axis acts as a primary driver that initiates both programs.

While our findings provide compelling evidence for the lactate–IGF2BP2–Nrf2 axis in driving CRC progression, further investigations are warranted to explore its prognostic and therapeutic implications. Specifically, the prognostic value of this axis across different molecular subtypes of CRC and its predictive significance for treatment response to various therapeutic modalities, including immunotherapy, require further elucidation. Additionally, examining the interplay between this axis and other ferroptosis regulators such as the lipid repair enzyme GPX4 may yield valuable insights into the complex regulatory networks governing ferroptosis sensitivity in CRC. Finally, it must be acknowledged that this study has certain limitations. Although we analysed 40 clinical CRC samples, revealing positive correlations among H3K18la, IGF2BP2 and Nrf2 and were further supported by bioinformatic data from larger cohorts, the sample size remains relatively modest. Future studies involving larger, well‐annotated clinical cohorts are essential to further validate the clinical relevance and prognostic value of this axis.

In summary, our study unravelled a novel mechanism by which the lactate‐rich TME confreres ferroptosis resistance in CRC cells and promotes an immunosuppressive milieu through M2 macrophage polarisation. We identified IGF2BP2 as a critical mediator of this process, whereby lactate‐induced lactylation of IGF2BP2 increases Nrf2 mRNA stability, leading to elevated Nrf2 levels and enhanced ferroptosis resistance. This regulatory axis not only desensitised CRC cells to ferroptosis induction but also shaped the TME to favour tumour progression, highlighting the therapeutic potential of targeting this pathway.

## AUTHOR CONTRIBUTIONS

*Conceptualisation, methodology, writing—original draft and funding acquisition*: Jin‐Feng Zhu. *Validation and investigation*: Da‐Peng Guo. *Resources and visualisation*: Hai‐Na Lv. *Formal analysis and data curation*: Zong‐Yu Liang. *Supervision and project administration*: Jing Song. *Supervision, writing—review and editing*: Wei Zeng.

## CONFLICT OF INTEREST STATEMENT

The authors declare no conflict of interest.

## DATE AVAILABILITY STATEMENT

The datasets generated during and/or analysed during the current study are available from the corresponding author on reasonable request.

## ETHICS STATEMENT

Informed consent was obtained from all subjects, and the study was approved by Committee of the Sixth Affiliated Hospital, School of Medicine, South China University of Technology. All animal experiments were approved by the Institutional Animal Care and Use Committee of the Sixth Affiliated Hospital, School of Medicine, South China University of Technology [No. NYKY‐2024‐15‐01].

## Supporting information



Supporting information
