# Supplementary material for: Histone lactylation‐mediated up‐regulation of IGF2BP2 enhances ferroptosis resistance via Nrf2 in colorectal cancer
Source: Clin Transl Med. 2025 Dec 15;15(12):e70551. doi: 10.1002/ctm2.70551 (PMC12706171; doi:10.1002/ctm2.70551)
Supplement: Supplementary file 1 — Supporting information [file CTM2-15-e70551-s001.pdf]

Supplementary Materials

1. Supplementary Figure and Figure legends

Supplementary Figure 1. IGF2BP2 and Nrf2 were upregulated in CRC.

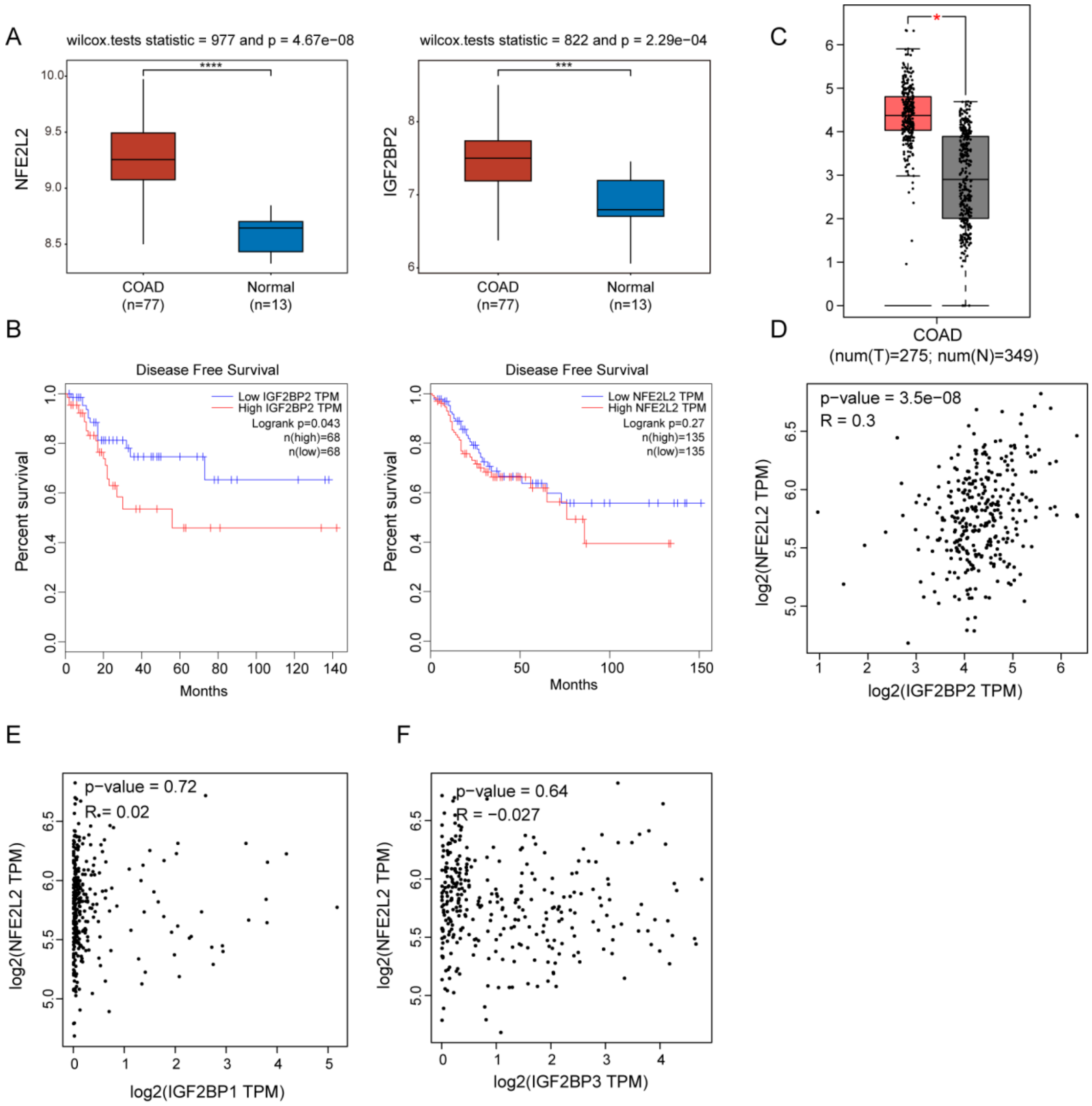

A. Transcript levels of IGF2BP2 and Nrf2 were assessed in colorectal adenocarcinoma (COAD) and matched normal tissues from publicly available GEO datasets (GSE24550, *n* =77). Differential expression was evaluated using RNA-seq quantification with thresholds of |log2FC| > 1 and adjusted ***$p < 0.001$. **B.** Disease-free survival analysis based on IGF2BP2 and Nrf2 expression levels from the

GEPIA database. Patients were stratified into high- and low-expression groups (IGF2BP2 High and low expression, *n* = 68; Nrf2 High and Low expression, *n* = 135). **C.** Box plots showing the differential expression of IGF2BP2 mRNA in COAD tissues (*n* = 275) versus adjacent normal tissues (*n* = 349) from The Cancer Genome Atlas (TCGA) database. Statistical significance was determined using Wilcoxon rank-sum test. $p < 0.05$. **D-F.** Correlation analysis between Nrf2 and IGF2BP2/IGF2BP1/IGF2BP3 mRNA expression (TPM, Transcripts Per Million) in COAD tissues from the TCGA database (*n* = 275). Pearson's correlation coefficient (R) and p-value are shown.

Supplementary Figure 2. Elevated lactylation, H3K18la and IGF2BP2 levels were associated with Nrf2-mediated ferroptosis resistance in CRC.

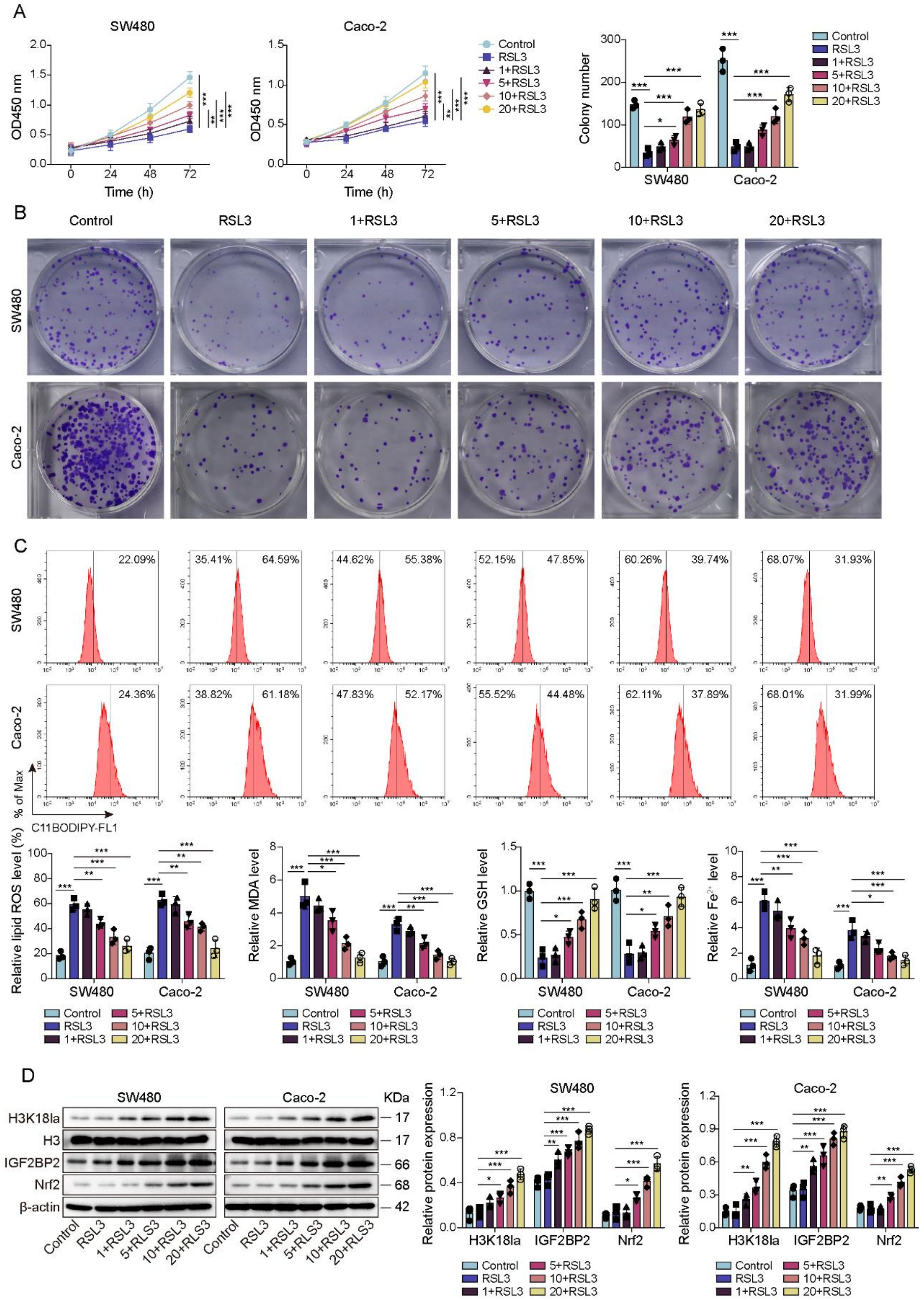

SW480 and Caco-2 cells were treated with different concentrations of lactate (0, 1, 5, 10, and 20 mM) for 12 h, followed by the ferroptosis inducer RSL3 for 24 h. Experimental groups: control, RSL3, 1+RSL3, 5+RSL3, 10+RSL3, and 20+RSL3. **A.**

Cell viability was measured by CCK-8. **B.** Colony formation assay was performed to evaluate cell proliferation. **C.** Lipid ROS, MDA, Fe^{2+}, and glutathione (GSH) levels were measured using commercial kits. **D.** H3K18la, Nrf2, and IGF2BP2 levels were examined by western blotting. The measurement data were presented as mean ± SD. $n = 3$ for A-D. *$p < 0.05$, **$p < 0.01$, ***$p < 0.001$.

Supplementary Figure 3. Ferrostatin-1 specifically rescued RSL3-induced ferroptosis in CRC cells.

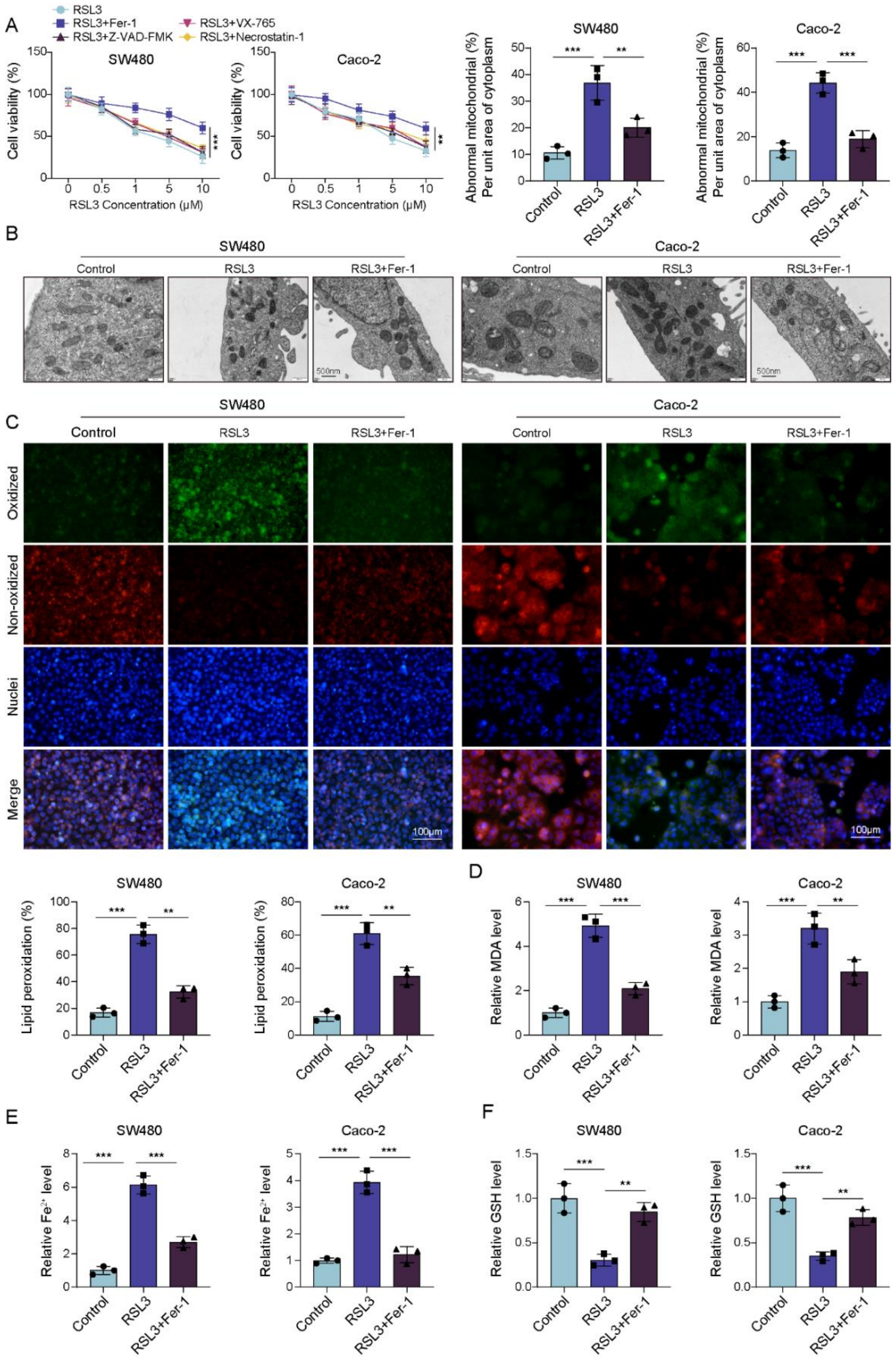

A. Cell viability of SW480 and Caco-2 cells treated with increasing concentrations of RSL3 (0, 0.5, 1, 5, 10 μM) for 24 h in the presence of ferroptosis inhibitor Ferrostatin-1 (Fer-1, 1 μM), apoptosis inhibitor Z-VAD-FMK (50 μM), necrosis

inhibitor Necrostatin-1 (Nec-1, 30 μM), or pyroptosis inhibitor VX-765 (20 μM) was assessed by CCK-8 assay. SW480 and Caco-2 cells treated with Control, RSL3 (1 μM), or RSL3+Ferrostatin-1 (1 μM) for 24 h, then **B.** Representative transmission electron microscopy (TEM) images of mitochondrial morphology in different groups. Scale bar = 500 nm. **C.** Representative immunofluorescence images of C11-BODIPY staining. Scale bar = 100 μm. **D-F.** Levels of MDA, Fe^{2+} and glutathione (GSH) measured by commercial kits. The measurement data were presented as mean ± SD. $n = 3$. $^{**}p < 0.01$, $^{***}p < 0.001$.

Supplementary Figure 4. Genetic knockdown of Nrf2 reverses lactate-mediated ferroptosis resistance in CRC cells.

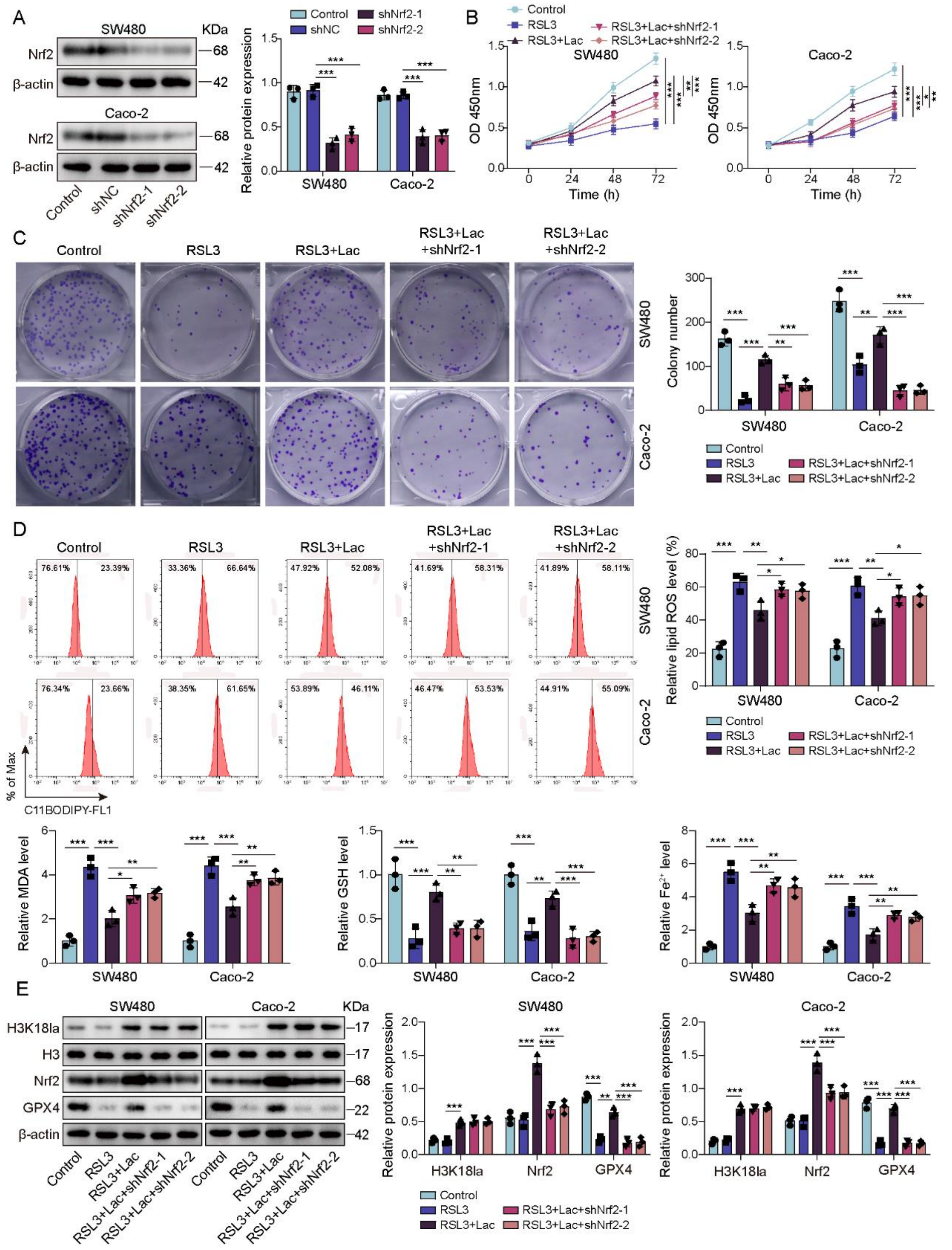

A. Western blot analysis of Nrf2 expression after transfection with shNrf2-1 or shNrf2-2. SW480 and Caco-2 cells with silenced Nrf2 were then treated with lactate or RSL3. Experimental groups: Control, RSL3, RSL3+Lac, RSL3+Lac+shNrf2-1, and RSL3+Lac+shNrf2-2. **B.** Cell viability was measured using the CCK-8 assay. **C.**

Colony formation assay was performed to evaluate cell proliferation. **D.** Lipid reactive oxygen species (ROS), MDA, Fe^{2+}, and glutathione (GSH) levels were measured using commercial kits. **E.** H3K18la, Nrf2, and GPX4 levels were examined by western blotting. Measurement data are presented as mean ± SD. *n* = 3. **p* < 0.05, ***p* < 0.01, ****p* < 0.001.

Supplementary Figure 5. Lactate promoted Nrf2-mediated ferroptosis resistance in CRC cells.

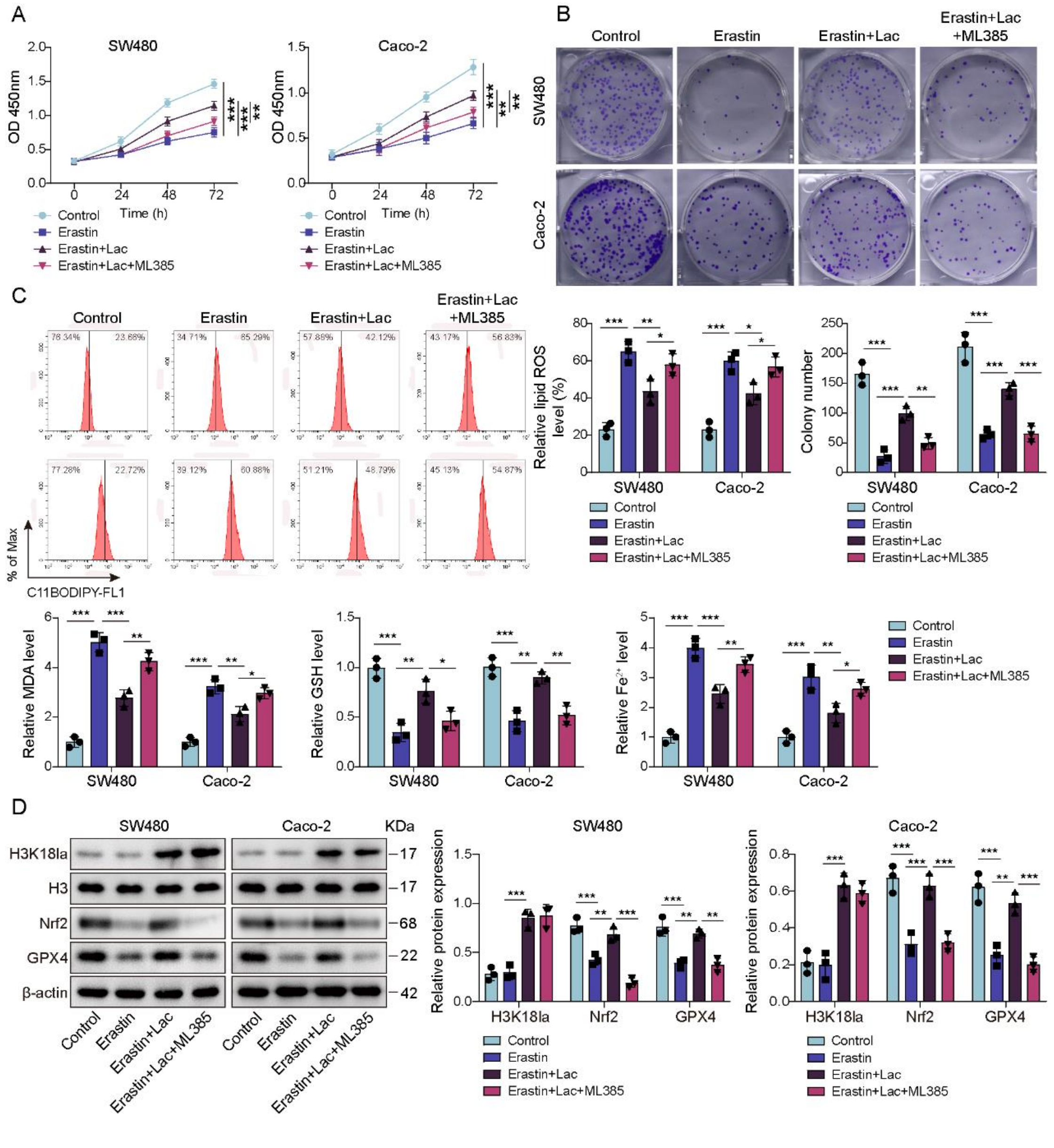

SW480 and Caco-2 cells were treated with lactate (10 mM) and the Nrf2 inhibitor ML385 (5 μM) for 24 h, followed by treatment with Erastin. Experimental groups: Control, Erastin, Lac+ Erastin, Lac+ Erastin +ML385. **A.** Cell viability was measured using the CCK-8 assay. **B.** Colony formation assay was performed to evaluate cell proliferation. **C.** Lipid ROS, MDA, Fe^{2+}, and glutathione (GSH) levels were measured using commercial kits. **D.** H3K18la, Nrf2, and GPX4 levels were examined using western blotting. Measurement data are presented as mean ± SD. $n = 3$. *$p < 0.05$, **$p < 0.01$, ***$p < 0.001$.

Supplementary Figure 6. Lactate promoted M2 macrophage polarization in CRC cells.

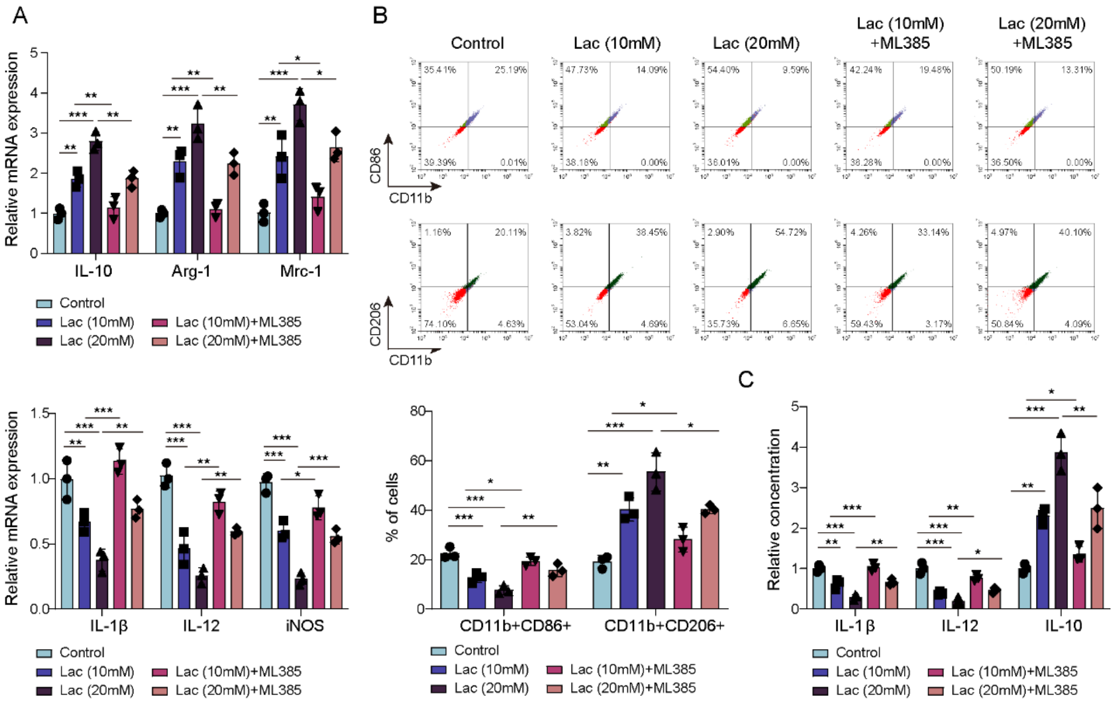

THP-1 cells were differentiated into macrophages (MΦ) by treating with PMA (200 nM) for 24 h, followed by treatment with different concentrations of lactate (0, 10, 20 mM) and the Nrf2 inhibitor ML385 (5 μM). Experimental groups: control, Lac (10

mM), Lac (20 mM), Lac (10 mM) + ML385, Lac (20 mM) + ML385. **A**. The mRNA levels of M1 markers (IL-1β, IL-12, iNOS) and M2 markers (IL-10, Arg-1, Mrc-1) were measured by RT-qPCR. **B**. Proportions of M1 (CD11b+CD86+) and M2 (CD11b+CD206+) macrophages were analyzed by flow cytometry. **C**. ELISA analysis of IL-1β, IL-12, and IL-10 secretion. The measurement data were presented as mean ± SD. $n = 3$. *$p < 0.05$, **$p < 0.01$, ***$p < 0.001$.

Supplementary Figure 7. Bioinformatics screening for RNA-binding proteins (RBPs) regulating Nrf2 mRNA stability.

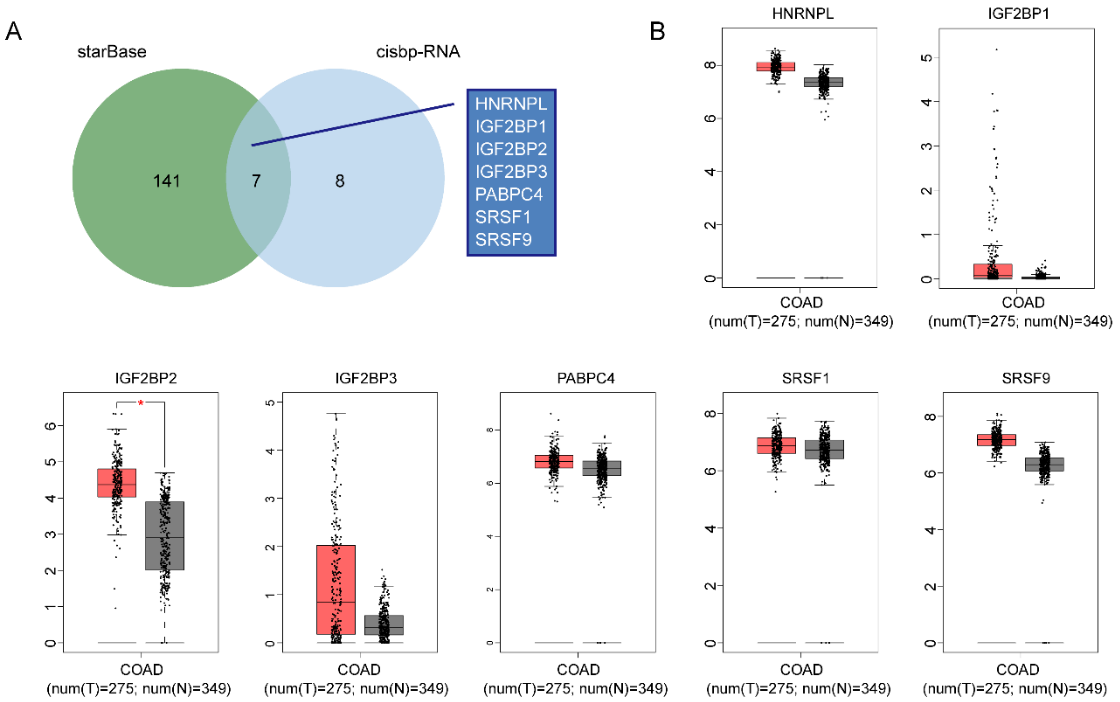

A. Potential Nrf2 mRNA-binding RBPs were predicted through intersection analysis of starBase and cisbp-RNA databases. **B.** Expression profiles of the 7 candidate RBPs (HNRNPL, IGF2BP1, IGF2BP2, IGF2BP3, PABPC4, SRSF1, and SRSF9) in COAD tumor tissues (T, n = 275) versus adjacent normal tissues (N, n = 349). Data were analyzed and plotted using the GEPIA2 database.

Supplementary Figure 8. IGF2BP2 promoted CRC cell ferroptosis resistance.

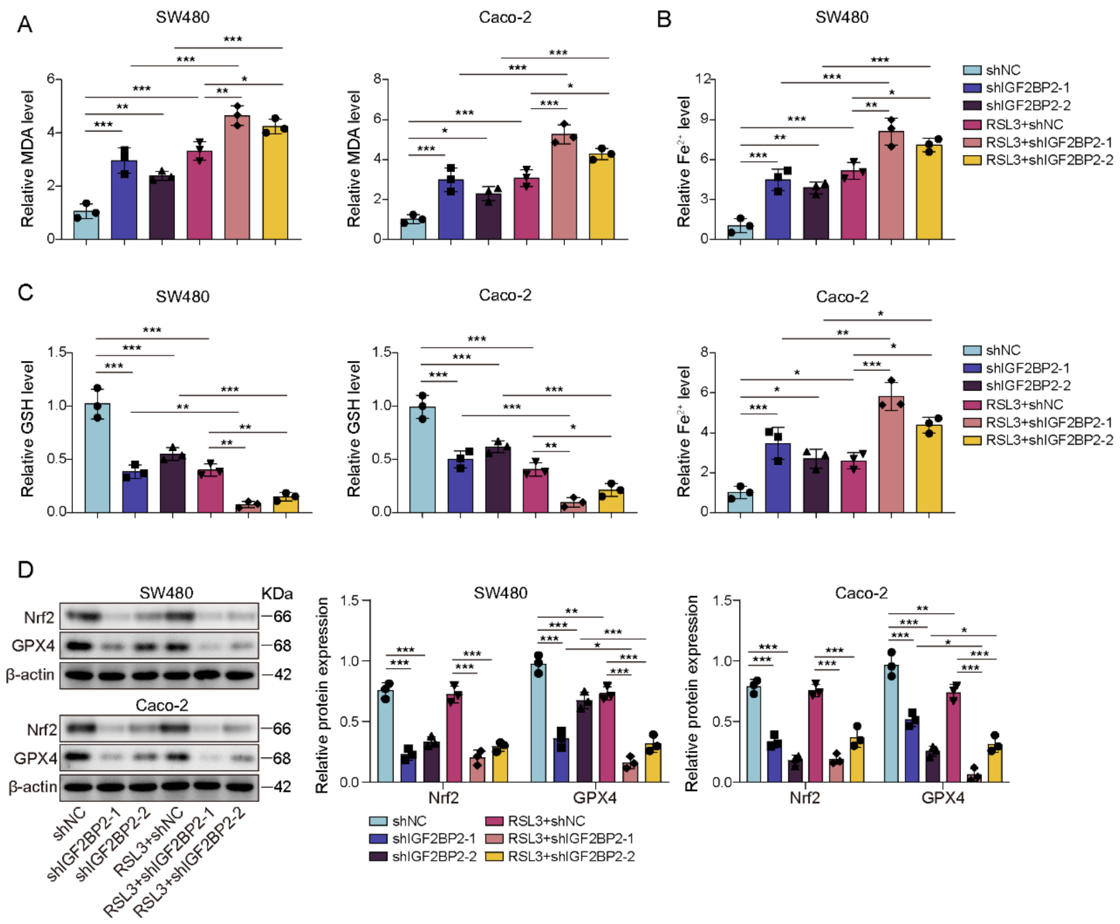

SW480 and Caco-2 cells transfected with shIGF2BP2 (IGF2BP2-KD). IGF2BP2-KD cells were treated with RSL3 and group as: shNC, shIGF2BP2-1, shIGF2BP2-2, RSL3+shNC, RSL3+ shIGF2BP2-1, and RSL3+shIGF2BP2-2. **A-C.** MDA, Fe^{2+}, and glutathione (GSH) levels measured using commercial kits. **D.** Western blot analysis of Nrf2 and GPX4 expression. Measurement data were presented as mean ± SD. $n = 3$. *$p < 0.05$, **$p < 0.01$, ***$p < 0.001$.

Supplementary Figure 9. IGF2BP2 promoted M2 macrophage polarization.

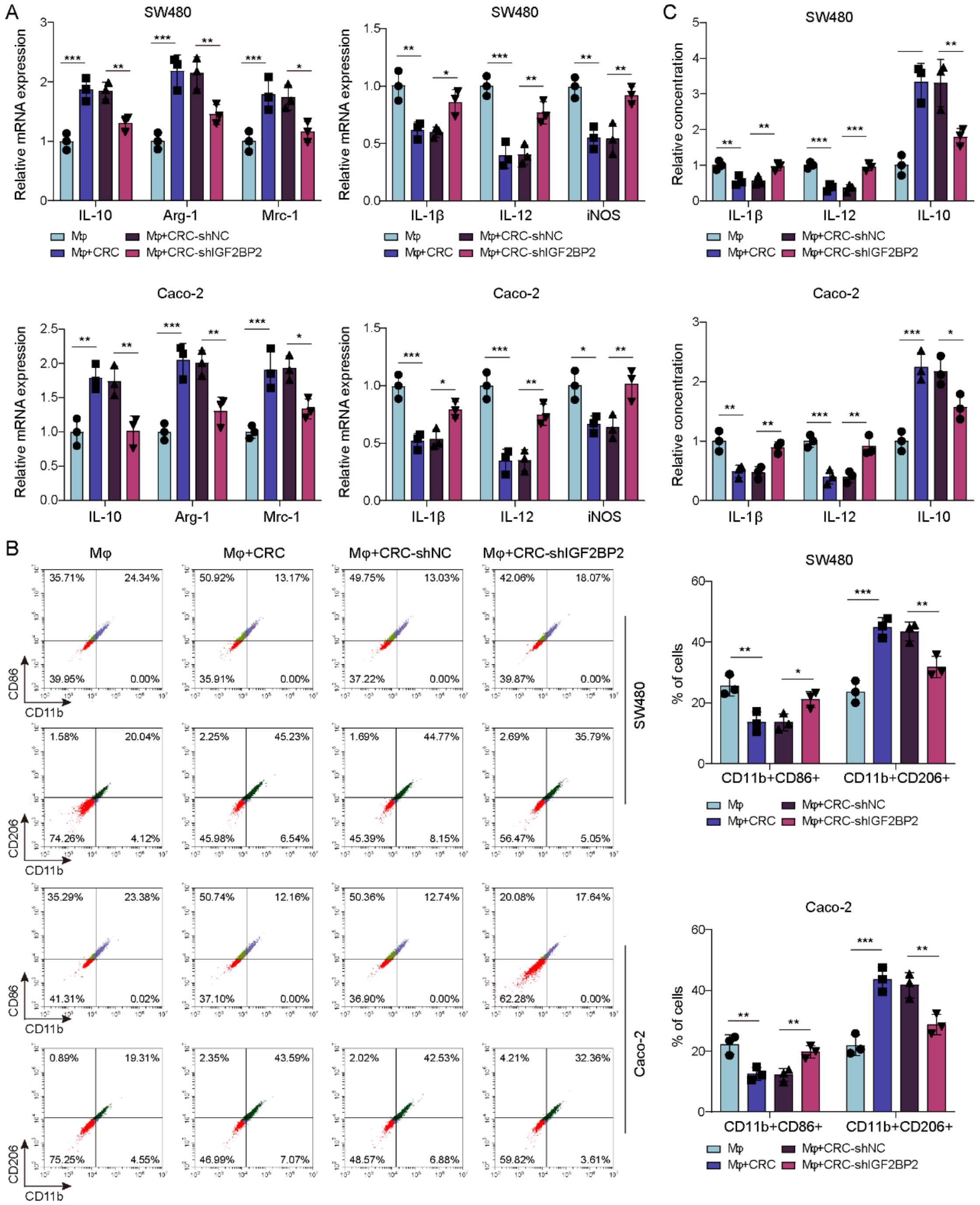

THP-1 cells were treated with 200 nM PMA for 24h to induce macrophages differentiation. macrophages were co-cultured with IGF2BP2-KD CRC cells and group as: Mφ, Mφ+CRC, Mφ+CRC-shNC, Mφ+CRC-shIGF2BP2. **A**. RT-qPCR analysis of IL-1β, IL-12, iNOS, IL-10, Arg-1, and Mrc-1. **B**. Flow cytometry quantification of M1 macrophage (CD11b+CD86+) and M2 macrophage (CD11b+CD206+) populations. **C**. ELISA analysis of IL-1β, IL-12 and IL-10

secretion. The measurement data were presented as mean ± SD. $n = 3$. *$p < 0.05$, **$p < 0.01$, ***$p < 0.001$.

Supplementary Figure 10. Pharmacological inhibition of IGF2BP2 reverses its protective effect against ferroptosis.

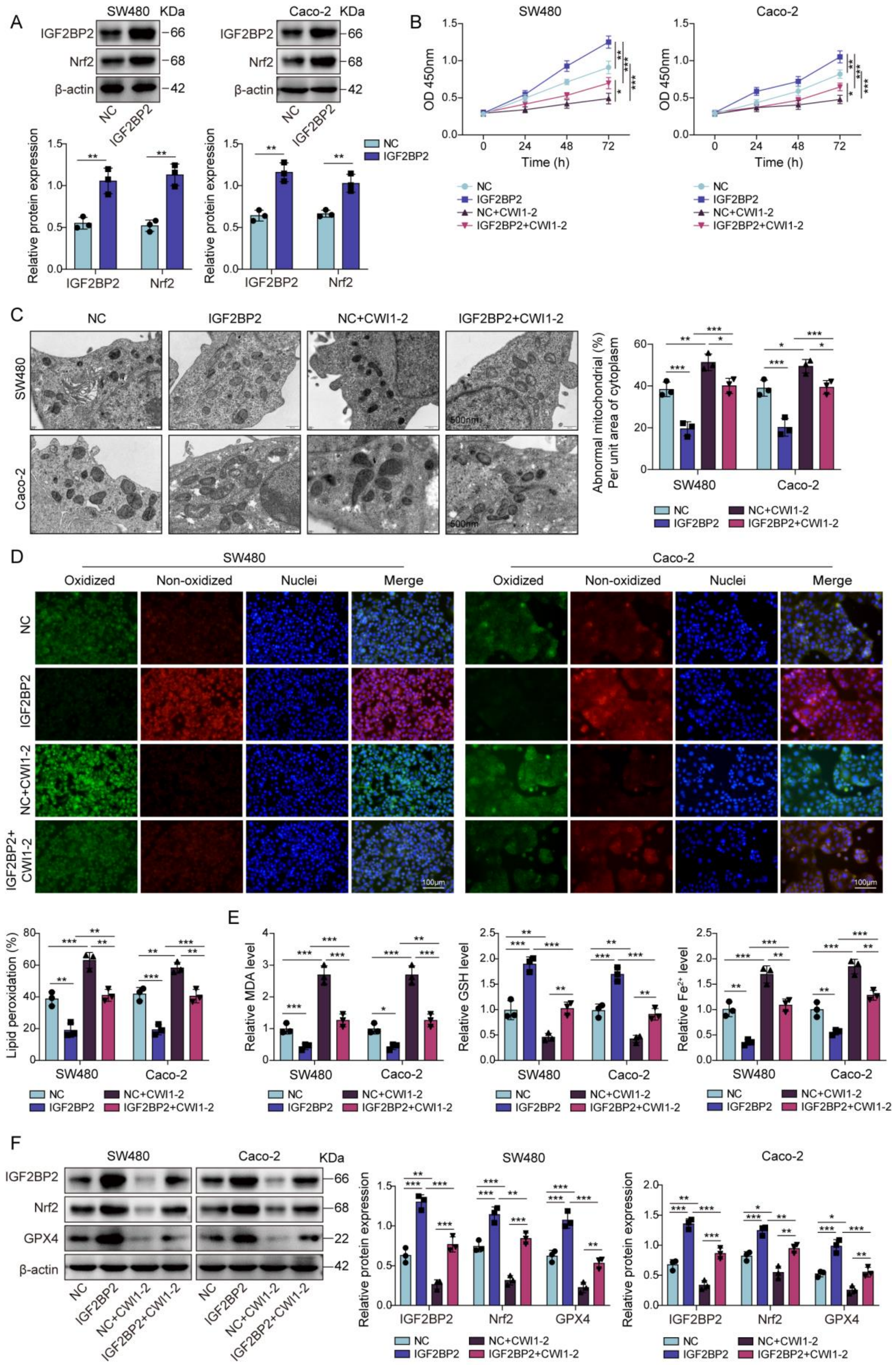

A. Western blot analysis validating the overexpression of IGF2BP2 and its effect on Nrf2 protein levels in SW480 and Caco-2 cells transfected with either a control vector (NC) or an IGF2BP2 overexpression plasmid (IGF2BP2). IGF2BP2-overexpressing

cells were treated with the IGF2BP2 inhibitor CWI1-2 in the presence of RSL3 to induce ferroptotic stress. **B.** Cell viability was measured by CCK-8 assay. **C.** Representative transmission electron microscopy (TEM) images showing mitochondrial morphology (Scale bar = 500 nm). **D.** Lipid peroxidation assessed by C11-BODIPY immunofluorescence (Scale bar = 100 μm). **E.** Quantification of intracellular MDA, Fe^{2+}, and glutathione (GSH) levels using commercial kits. **F.** Western blot analysis of IGF2BP2, Nrf2, and GPX4 protein expression. Measurement data are presented as mean ± SD. $n = 3$. *$p < 0.05$, **$p < 0.01$, ***$p < 0.001$.

Supplementary Figure 11. EP300 and H3K18 lactylation were required for IGF2BP2 transcriptional activation.

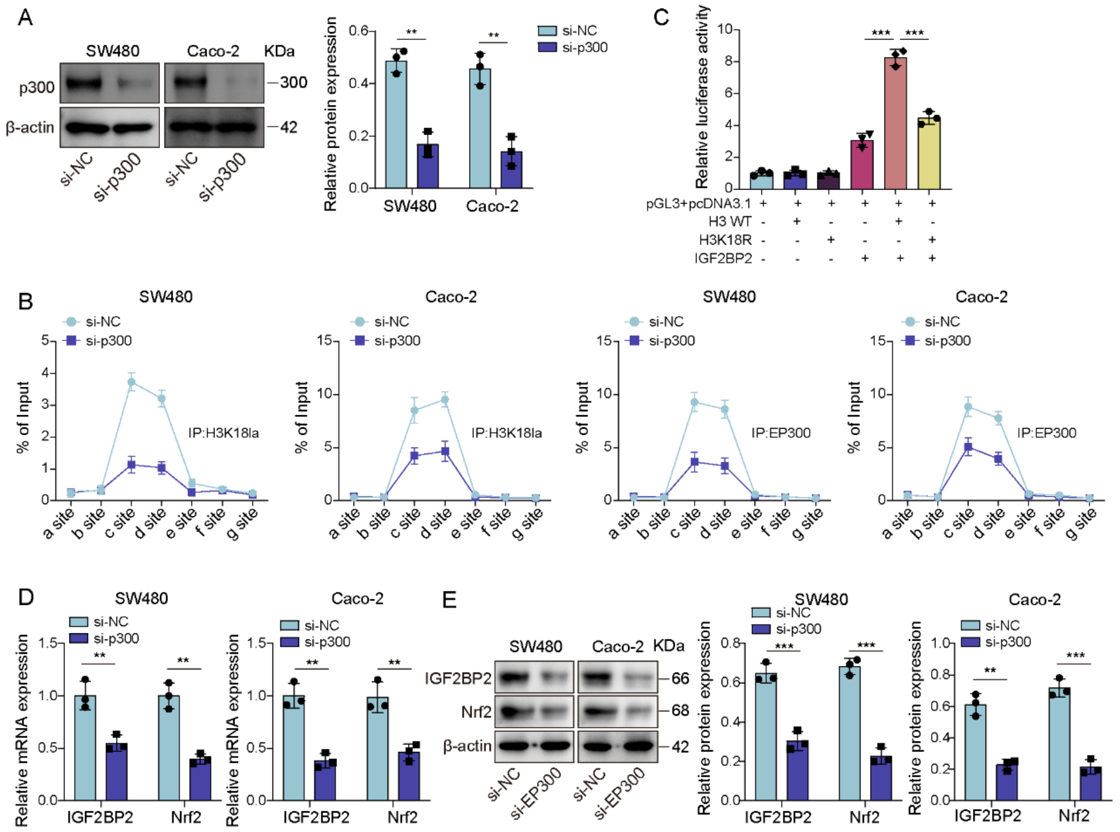

A. Western blot analysis of EP300 levels in SW480 and Caco-2 cells transfected with si-p300. **B.** ChIP-qPCR analysis of the enrichment of H3K18la and EP300 at the IGF2BP2 promoter in SW480 and Caco-2 cells transfected with si-p300. **C.**

Dual-luciferase reporter assay in HEK293T cells co-transfected with IGF2BP2 promoter-luciferase reporter and either H3-WT or H3K18R-Mut. **D and E.** RT-qPCR and western blot analysis of IGF2BP2 and Nrf2 expression in SW480 and Caco-2 cells transfected with si-EP300. The measurement data were presented as mean ± SD. *n = 3*. **$p < 0.01$, ***$p < 0.001$.

Supplementary Figure 12. Glycolysis inhibition decreased protein lactylation in CRC cells.

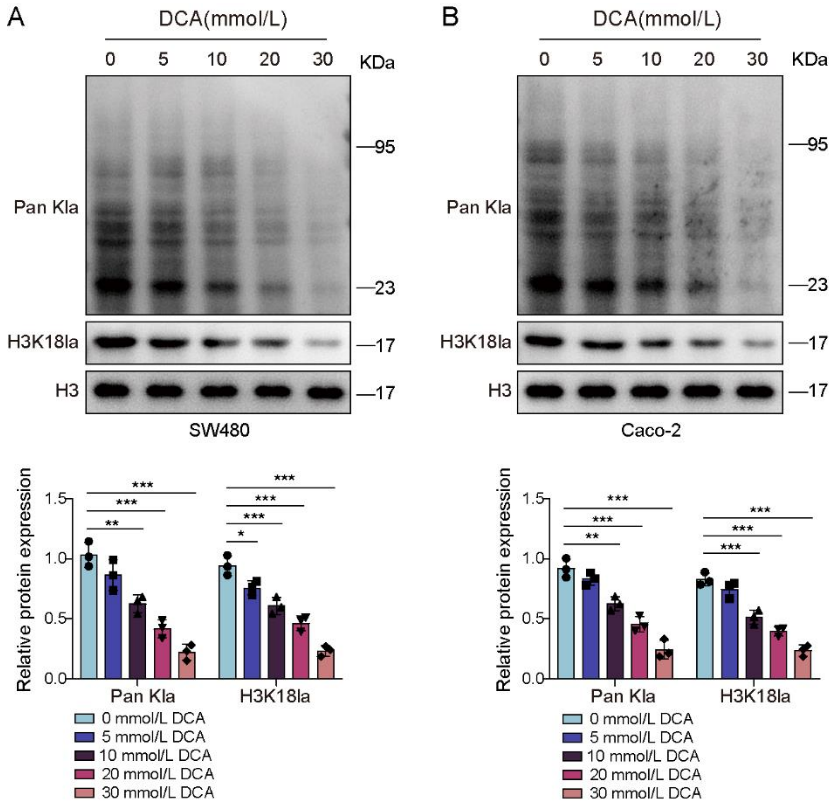

A and B. SW480 and Caco-2 cells were treated with glycolysis inhibitors DCA (0-30 mmol/L) for 24h, then pan Kla and H3K18la levels were determined by western blotting. The measurement data were presented as mean ± SD. *n = 3*. *$p < 0.05$, **$p < 0.01$, ***$p < 0.001$.

Supplementary Figure 13. AZ-33 inhibited CRC cell proliferation and metastasis

by inhibiting ferroptosis resistance and lactate promotes H3K18la modification in a manner dependent on GPR81 signaling.

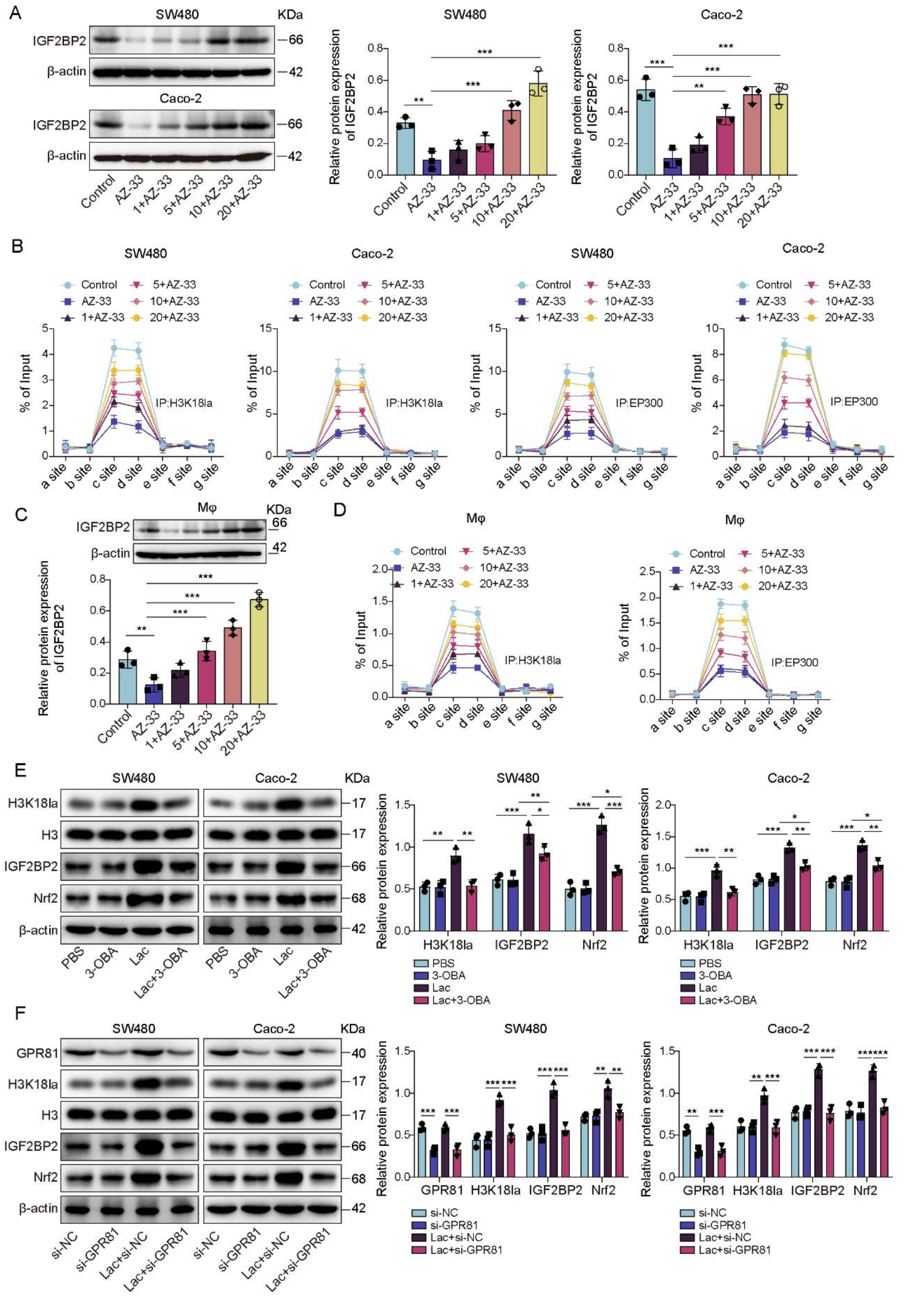

SW480 and Caco-2 cells were pretreated with the glycolysis inhibitor AZ-33 (0.5 μM)

for 12h, then stimulated with lactate (0, 1, 5, 10, 20 mM) for 24 h, group as: control, AZ-33, Lac (1 mM) + AZ-33, Lac (5 mM) + AZ-33, Lac (10 mM) + AZ-33, Lac (20 mM) + AZ-33. **A.** Western blot analysis of IGF2BP2 expression. **B.** ChIP-qPCR of H3K18la and EP300 in the IGF2BP2 promoter. THP-1 cells were differentiated into macrophages with PMA (200 nM) for 24 h, pretreated with AZ-33, and stimulated with lactate (0, 1, 5, 10, and 20 mM) for 24 h. **C.** Western blot analysis of IGF2BP2 expression in macrophages. **D**. ChIP-qPCR of H3K18la and EP300 at the IGF2BP2 promoter in the macrophages. **E.** Western blot analysis of H3K18la, IGF2BP2, and Nrf2 expression in SW480 and Caco-2 cells pre-treated with the GPR81 inhibitor 3-OBA (5 mM) for 1h, followed by stimulation with lactate for 24h. **F.** Western blot analysis of GPR81, H3K18la, IGF2BP2, and Nrf2 expression in SW480 and Caco-2 cells transfected with si-GPR81 for 48h, followed by treatment with lactate for an additional 24h. Measurement data are presented as mean ± SD. n = 3. *p < 0.05, **p < 0.01, ***p < 0.001.

Supplementary Figure 14. IGF2BP2 specifically bound to the m6A modification site of Nrf2 mRNA.

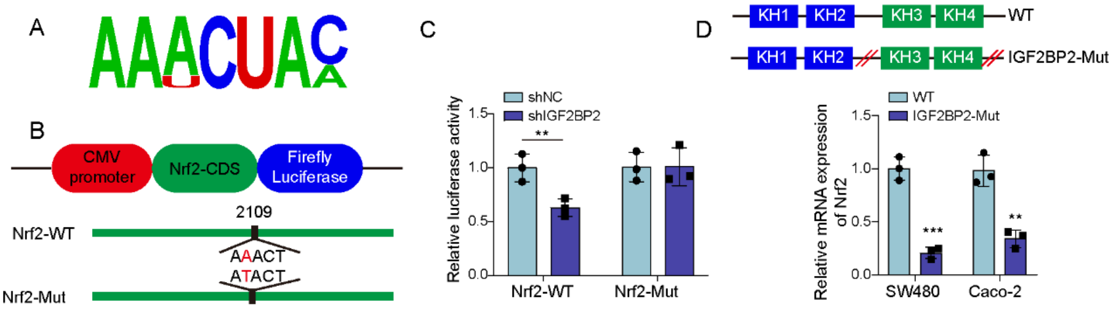

A. Schematic representation of IGF2BP2 binding motifs obtained from RM2Target database. **B.** Prediction of m^6A methylation sites in Nrf2 mRNA using deepSRAMP database (http://www.cuilab.cn/sramp/). **C.** Dual-luciferase reporter assay detected the relative luciferase activity in HEK293T cells transfected with shNC or shIGF2BP2, followed by transfection with Nrf2-WT or Nrf2-Mut reporter constructs. **D.** RT-qPCR analysis of Nrf2 expression after treated with IGF2BP2-WT and IGF2BP2-Mut (KH3-4-deleted) vectors in SW480 and Caco-2 cells. The measurement data were presented as mean ± SD. $n = 3$. $^{**}p < 0.01$, $^{***}p < 0.001$.

Supplementary Figure 15. Lactate promoted CRC cell ferroptosis resistance by regulating IGF2BP2–Nrf2 axis.

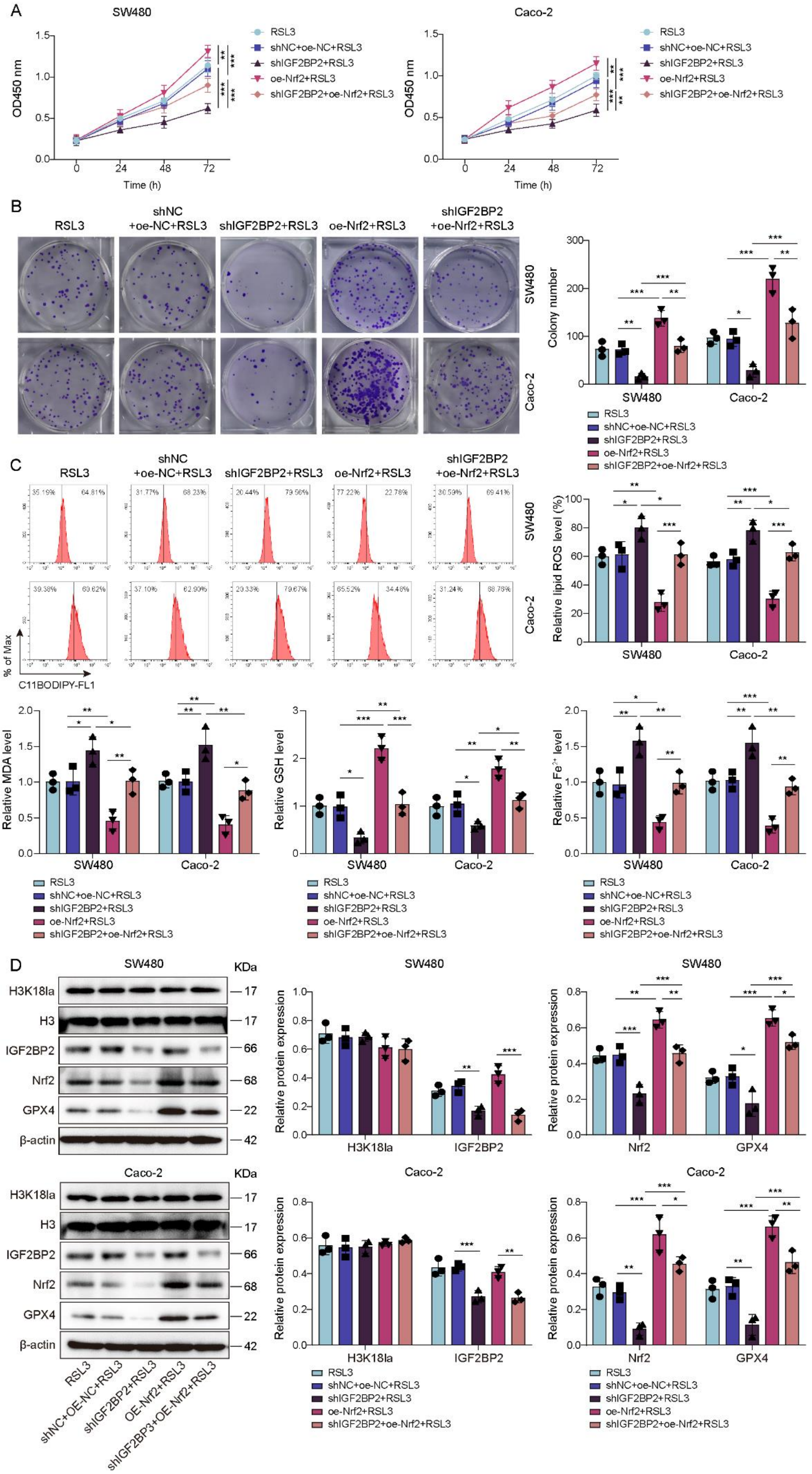

A
SW480
Caco-2
OD450 nm
Time (h)
RSL3
shNC+oe-NC+RSL3
shIGF2BP2+RSL3
oe-Nrf2+RSL3
shIGF2BP2+oe-Nrf2+RSL3
B
shNC
+oe-NC+RSL3
shIGF2BP2
+oe-Nrf2+RSL3
Colony number
C
Relative lipid ROS level (%)
% of Max
C11BODIPY-FL1
Relative MDA level
Relative GSH level
Relative Fe2+ level
D
KDa
H3K18la
H3
IGF2BP2
Nrf2
GPX4
β-actin
Relative protein expression
RSL3
shNC+OE-NC+RSL3
shIGF2BP2+RSL3
OE-Nrf2+RSL3
shIGF2BP3+OE-Nrf2+RSL3

SW480 and Caco-2 cells pretreated with lactate (10 mM) and RSL3 were transfected with shIGF2BP2 and/or OE-Nrf2, and group as: RSL3, shNC+OE-NC+RSL3, shIG2FBP2+RSL3, OE-Nrf2+RSL3, and shIGF2BP2+OE-Nrf2+RSL3. **A.** Cell viability was assessed by CCK-8 assay. **B.** Colony formation measured proliferative capacity. **C.** Lipid reactive oxygen species (ROS), levels of MDA, Fe^{2+}, and glutathione (GSH) were measured by commercial kits. **D.** Western blot analysis of H3K18la, IGF2BP2, Nrf2, and GPX4 levels. Measurement data are presented as mean ± SD. $n = 3$. *$p < 0.05$, **$p < 0.01$, ***$p < 0.001$.

Supplementary Figure 16. Lactate-treated macrophages promote CRC cell proliferation, migration, and invasion through paracrine signaling.

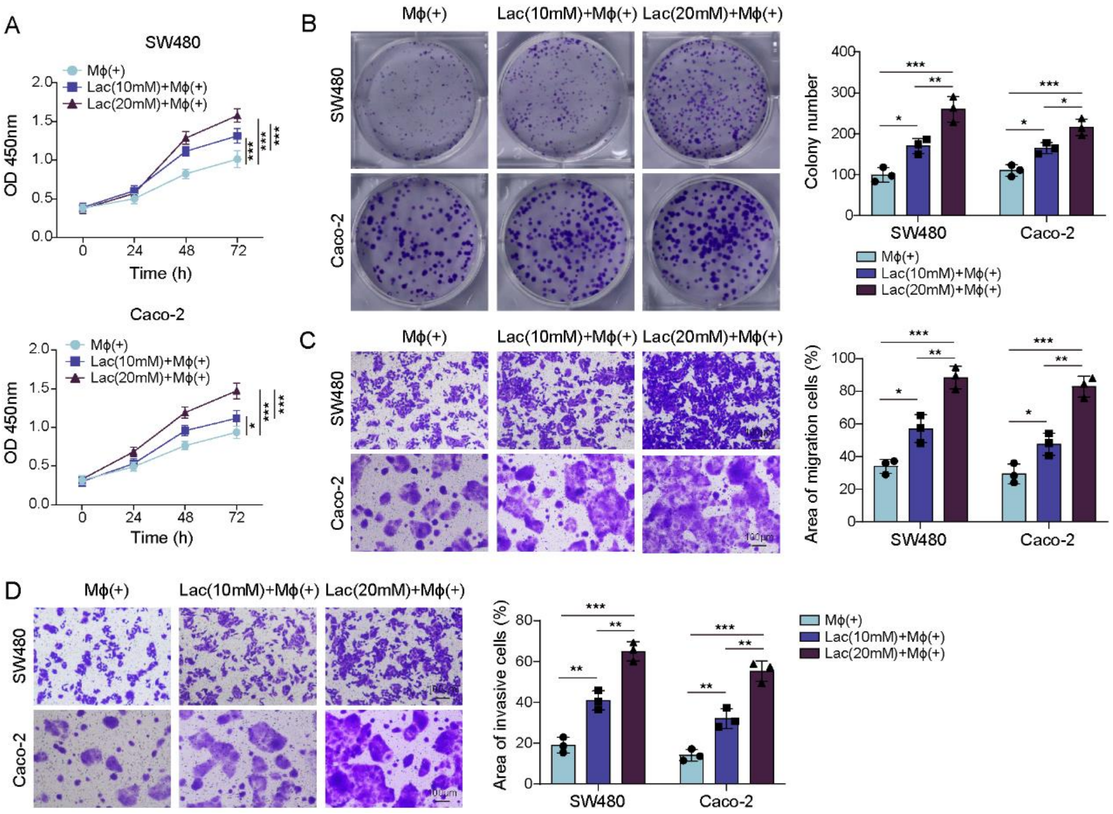

THP-1 cells were differentiated into macrophages using PMA (200 nM) for 24 h, then treated with different concentrations of lactate (0, 10, 20 mM) for 24 h. Treatment groups: Mφ(+) (control macrophages), Lac(10 mM)+Mφ(+), and Lac(20 mM)+Mφ(+). Lactate-treated macrophages were then co-cultured with SW480 or Caco-2 cells using Transwell systems for 24 h. **A.** Cell viability of SW480 and Caco-2 cells co-cultured with lactate-treated macrophages was assessed by CCK-8. **B.** Colony formation analysis of the number of colonies formed by CRC cells after co-culture with lactate-treated macrophages. **C-D.** Transwell migration and invaion analysis of CRC cells. Scale bar = 100 μm. The measurement data were presented as mean ± SD. $n = 3$. $^{*}p < 0.05$, $^{**}p < 0.01$, $^{***}p < 0.001$.

Supplementary Figure 17. Lactate promoted CRC cell M2 macrophage polarization by regulating IGF2BP2–Nrf2 axis.

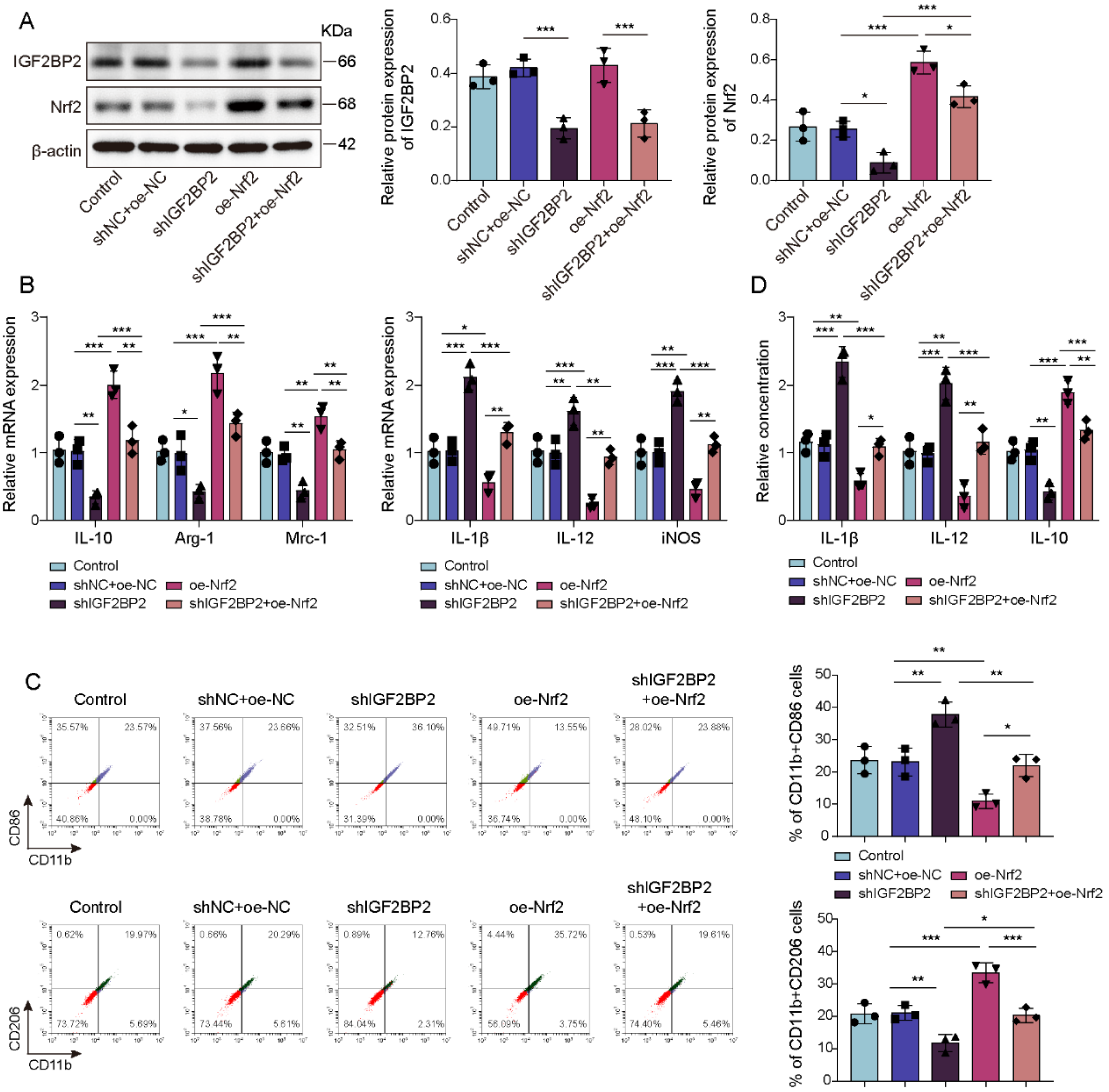

THP-1 cells induced by PMA (200 nM) were transfected with shIGF2BP2 and/or OE-Nrf2 and group as: control, shNC + OE-NC, shIGF2BP2, OE-Nrf2, or shIGF2BP2+OE-Nrf2. **A.** Western blot analysis of IGF2BP2 and Nrf2 levels. **B.** RT-qPCR analysis of IL-1β, IL-12, iNOS, IL-10, Arg-1, and Mrc-1. **C.** Flow cytometric quantification of M1 and M2 macrophage populations. **D.** ELISA analysis of IL-1β, IL-12 and IL-10 secretion. Measurement data are presented as mean ± SD. n = 3. *$p < 0.05$, **$p < 0.01$, ***$p < 0.001$.

Supplementary Figure 18. LDHA overexpression promotes M2 macrophage polarization in mice.

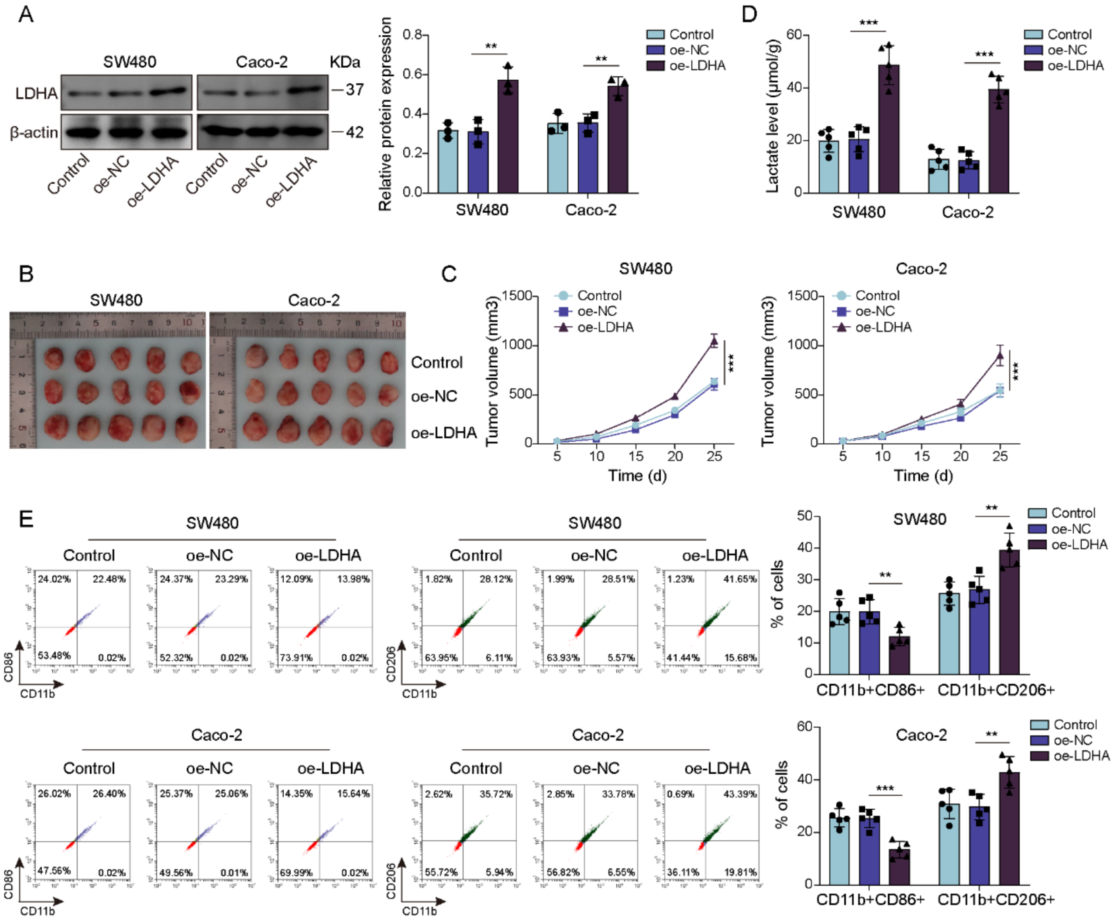

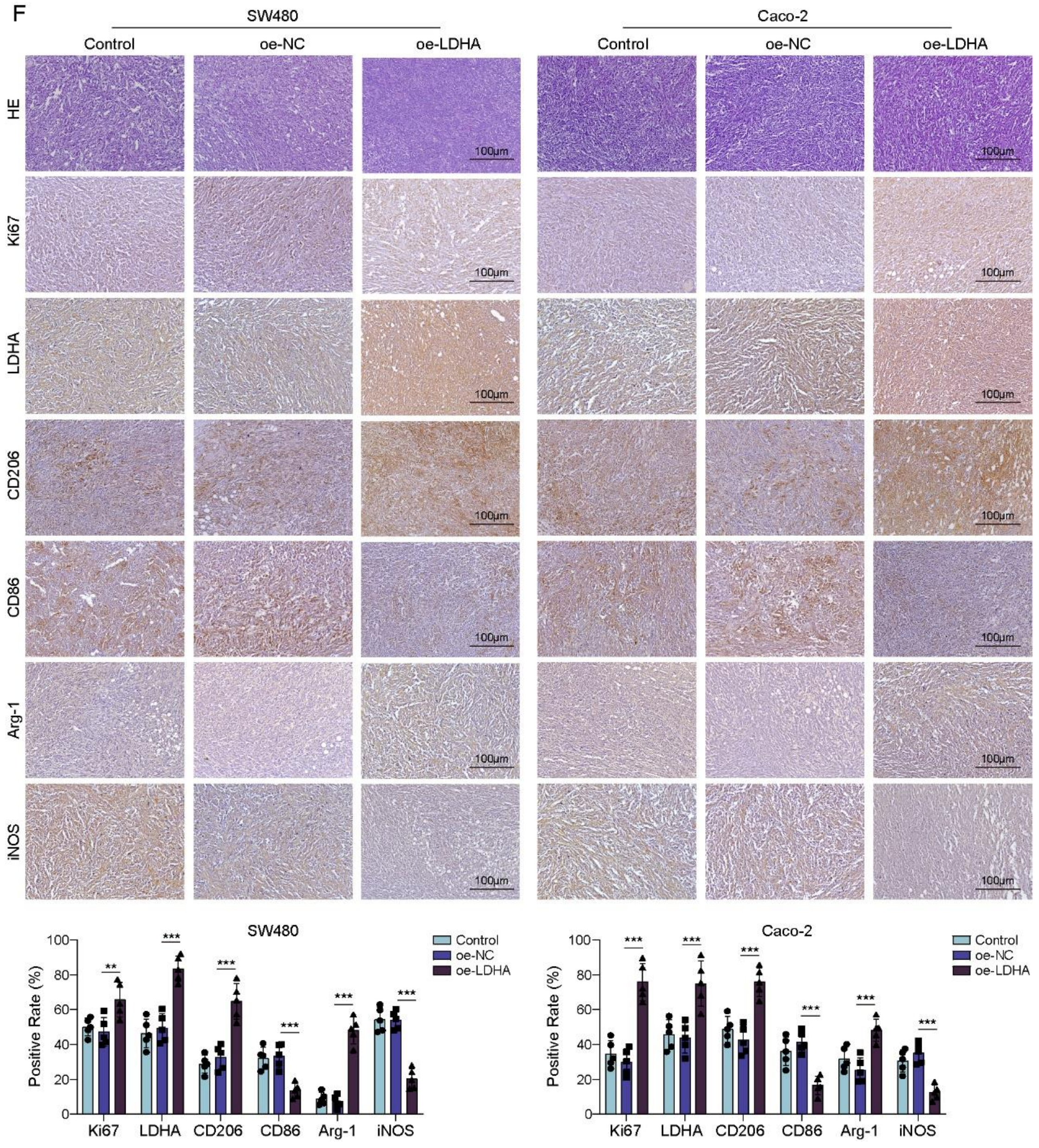

SW480 and Caco-2 cells were stably transfected with LDHA overexpression vector and injected subcutaneously into nude mice. Treatment groups: Control (parental cells), OE-NC (empty vector), and OE-LDHA. **A.** Western blot validation of LDHA expression in transfected cell lines. **B.** Representative images of excised tumors from different groups. **C.** Tumor growth curves monitored over 25 days. **D.** Lactate concentration in tumor tissues measured by lactate assay kit. **E.** Flow cytometry

analysis of tumor-associated macrophages showing percentage of CD11b+CD86+ (M1) and CD11b+CD206+ (M2) cells. **F.** IHC analysis of H&E, Ki67, LDHA, CD206, CD86, Arg-1, and iNOS expression in tumor sections. Scale bar = 100 µm. The measurement data were presented as mean ± SD. *n* = 5. ***p* < 0.01, ****p* < 0.001.

Supplementary Figure 19. DCA inhibited CRC cell proliferation and metastasis in mice by repressing ferroptosis resistance.

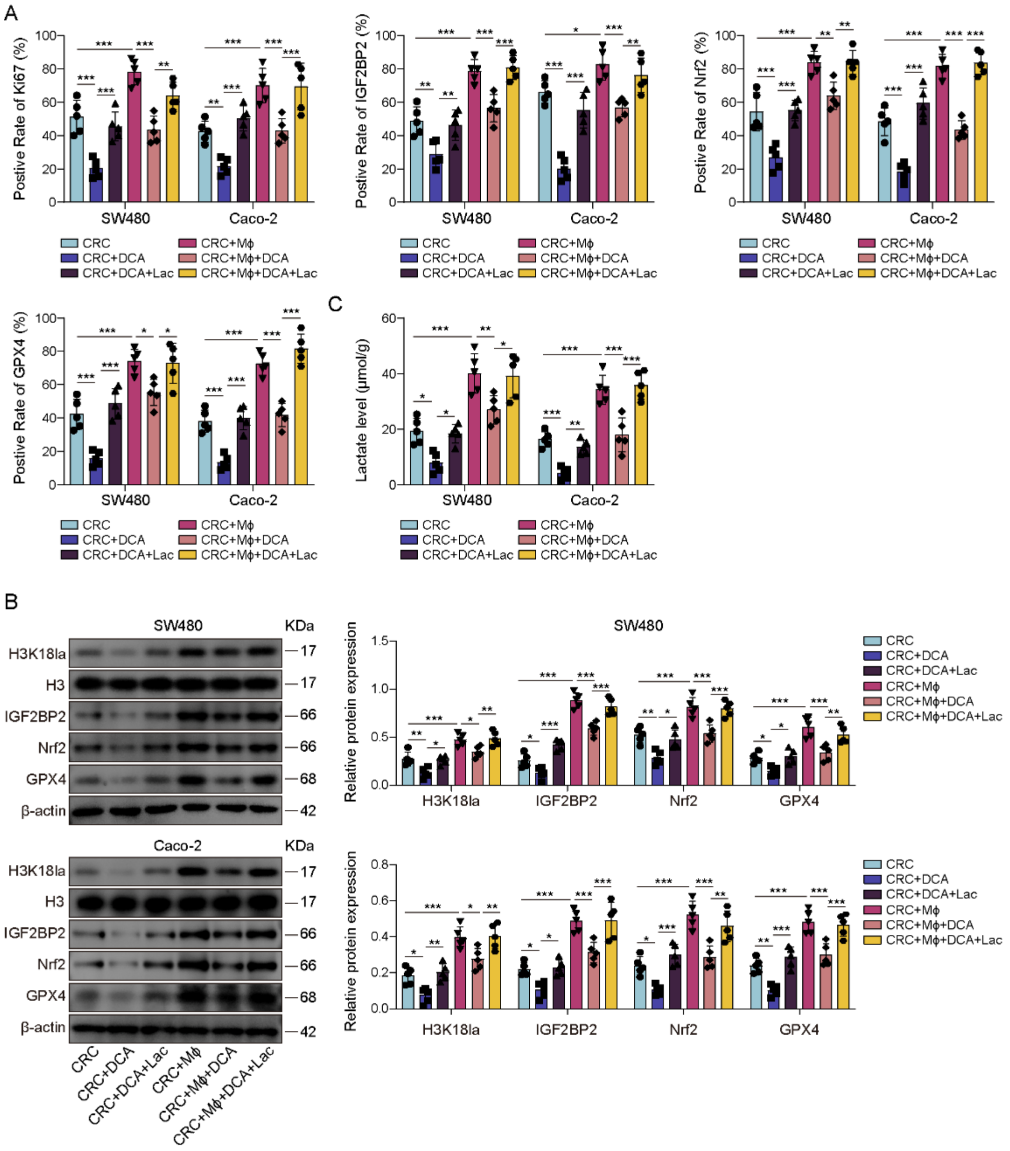

SW480 and Caco-2 cells were co-injected with or without THP-1-derived

macrophages (MΦ) subcutaneously into nude mice pre-treated with clodronate liposomes to deplete endogenous macrophages. Treatment groups: CRC (control), CRC+DCA, CRC+DCA+Lactate, CRC+MΦ, CRC+MΦ+DCA, and CRC+MΦ+DCA+Lactate. DCA (dichloroacetate, 50 mg/kg) was administered intraperitoneally every other day. **A.** Quantification of Ki67, IGF2BP2, Nrf2, and GPX4 expression through by IHC analysis in tumor tissues. **B.** Western blot analysis of tumor lysates examining H3K18la, IGF2BP2, Nrf2, and GPX4 expression levels. **C.** Lactate concentration in tumor tissues measured by lactate assay kit. Measurement data are presented as mean ± SD. $n = 5$. *$p < 0.05$, **$p < 0.01$, ***$p < 0.001$.

Supplementary Figure 20. AZ-33 inhibited CRC cell proliferation and metastasis in mice by repressing ferroptosis resistance.

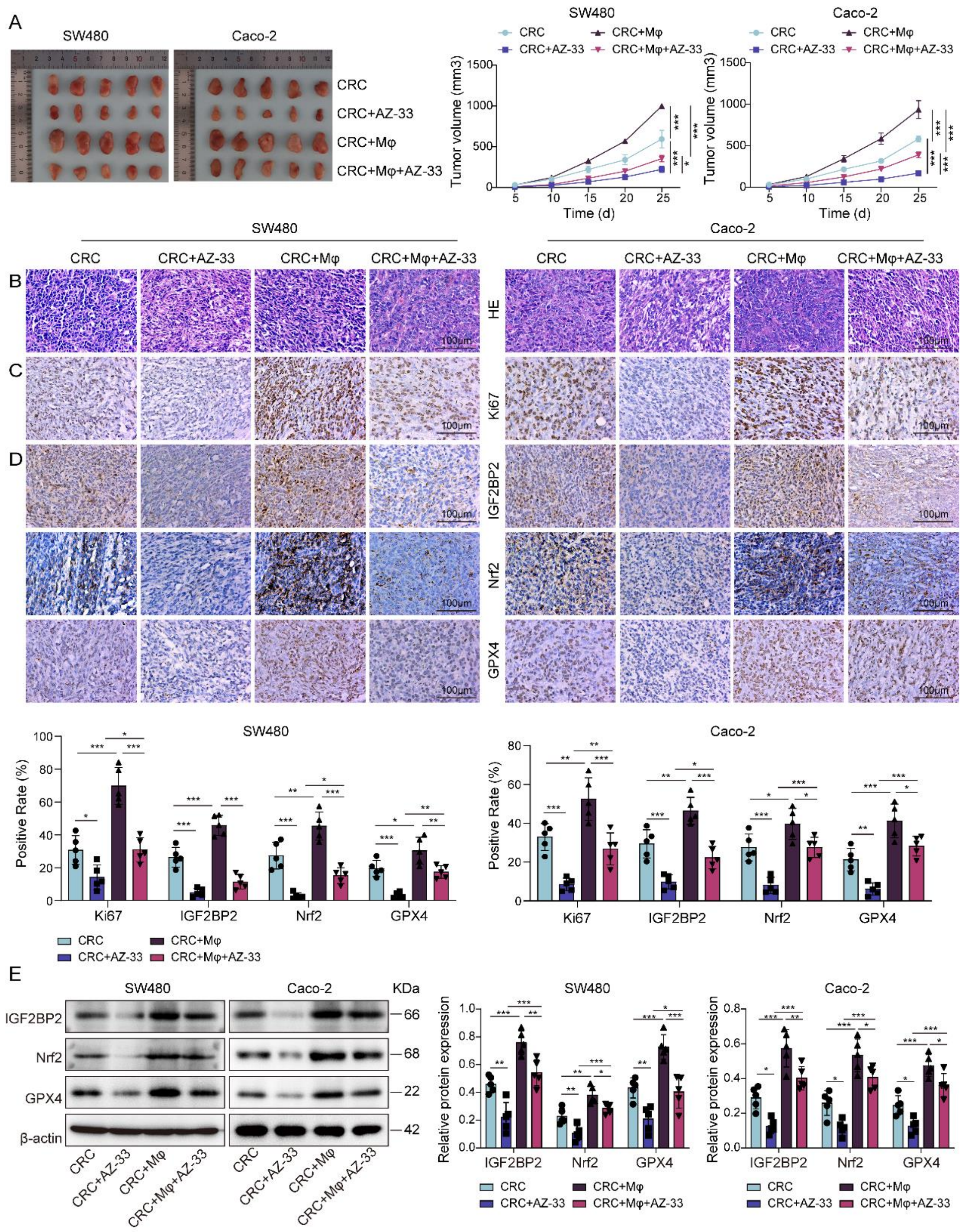

SW480 and Caco-2 cells were pretreated with AZ-33 for 12h. THP-1 cells were differentiated into macrophages by PMA treatment and then treated with AZ-33 for 12h. macrophages were subcutaneously co-injected with CRC cells into nude mice pretreated with clodronate liposomes to deplete endogenous macrophages. Once tumors were established, mice received intraperitoneal RSL3 injections and group as:

CRC, CRC+AZ-33, CRC+MΦ, CRC+MΦ+AZ-33. **A.** Tumor size and volume were monitored every five days. **B.** H&E staining of tumor sections (scale bar = 100 μm). **C.** IHC analysis of Ki67 (scale bar = 100 μm). **D-E.** IHC and western blot analysis of IGF2BP2, Nrf2 and GPX4 levels in tumor tissues (scale bar = 100 μm). Measurement data are presented as mean ± SD. $n = 5$. *$p < 0.05$, **$p < 0.01$, ***$p < 0.001$.

Supplementary Figure 21. IGF2BP2 knockdown inhibited CRC cell metastasis in mice.

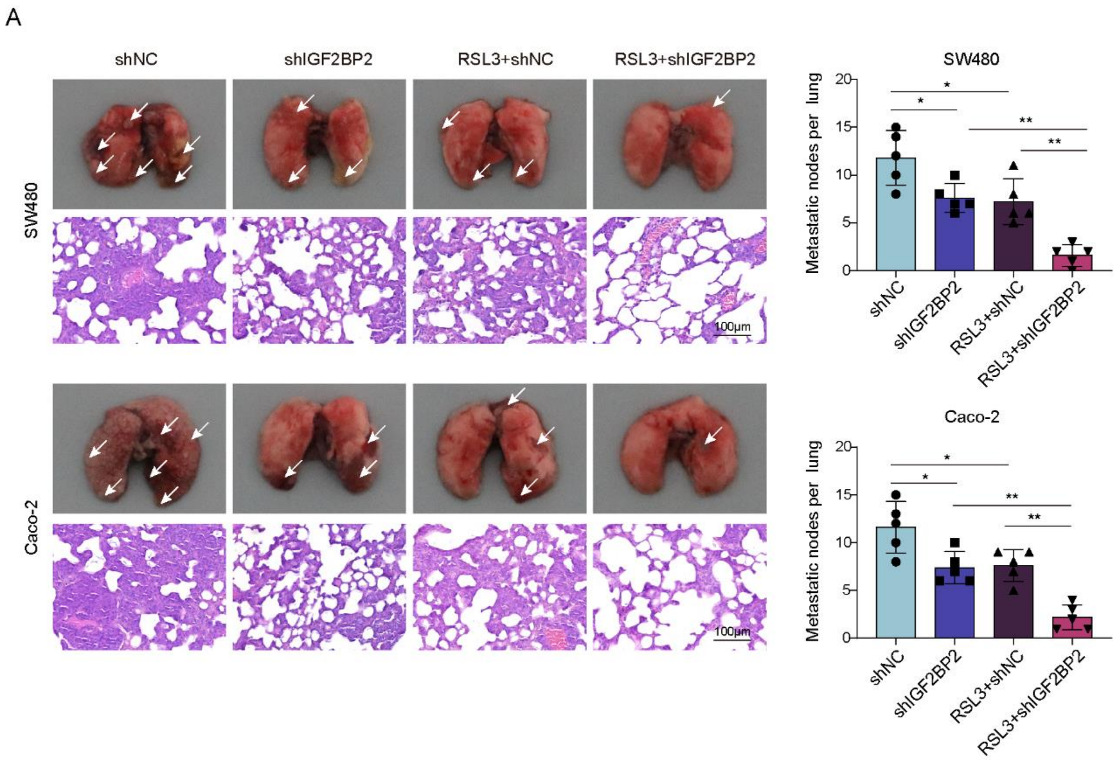

A. SW480 and Caco-2 cells stably transfected with shNC, shIGF2BP2, RSL3+shNC, or RSL3+shIGF2BP2 were injected into the tail veins of nude mice. Representative macroscopic images of lungs with metastatic nodules (white arrows) are shown in the top rows. H&E staining of lung sections (low rows) showing metastatic lesions (scale

bar: 100 μm). Measurement data are presented as mean ± SD. $n = 5$. *$p < 0.05$, **$p < 0.01$.

2. Supplementary Table and Table legends

Supplementary Table 1. Clinicopathological characteristics of 40 cases of CRC patients.

Parameters	Number of cases (%)
**Gender**	
Male	22 (55.0%)
Female	18 (45.0%)
**Age**	
≤60	17 (42.5%)
>60	23 (57.5%)
**Tumor size (cm)**	
≤5	21 (52.5%)
>5	19 (47.5%)
**Lymph node metastasis**	
N0	26 (65.0%)
N+	14 (35.0%)
**Distant metastasis**	
M0	19 (47.5%)
M1	21 (52.5%)
**TNM**	
I/II	23 (57.5%)
III/IV	17 (42.5%)

Supplementary Table 2. The primers listed in the RT-qPCR.

Name	Sequence (5'-3')
IL-10-F	GAGAACAGCTGCACCCACTT

IL-10-F	TCACATGCGCCTTGATGTCT
Arg1-F	ACTTAAAGAACAAGAGTGTGATGTG
Arg1-R	CATGGCCAGAGATGCTTCCA
Mrc1-F	ACCTGCGACAGTAAACGAGG
Mrc1-R	TGTCTCCGCTTCATGCCATT
IL-1β-F	AGCTCGCCAGTGAAATGATG
IL-1β-R	TGGTCGGAGATTCGTAGCTG
IL-12-F	TTGAGGTCATGGTGGATGCC
IL-12-R	CCTGGACCTGAACGCAGAAT
iNOS-142-F	CGCATGACCTTGGTGTTTGG
iNOS-142-R	CATAGACCTTGGGCTTGCCA
IGF2BP2-F	TGGAAGCGCATATCAGAGTG
IGF2BP2-R	AGTGCCCGATAATTCTGACG
Nrf2-F	GAATTTCTCCCAATTCAGCCAGCC
Nrf2-R	TCTGCGCCAAAAGCTGCATG
GAPDH-F	CCAGGTGGTCTCCTCTGA
GAPDH-R	GCTGTAGCCAAATCGTTGT

Supplementary Table 3. The primers listed in the ChIP-qPCR.

Name	Sequence（5'-3'）
ChIP-Nrf2-g-F	TTGGCTAAACCAAATGCCAAACAG
ChIP-Nrf2-g-R	ACTTTTCCAATGCTGCAATTTGCT
ChIP-Nrf2-f-F	TGTCTCTGCATCTTTCATGTGAG
ChIP-Nrf2-f-R	ATTGAAGCAAGCTTCTTTAACTCC
ChIP-Nrf2-e-F	CTAGAGAAAGTAAGCTCTGCAGC
ChIP-Nrf2-e-R	GGTTTTCAGTTTCTAGCTTCCTGG

ChIP-Nrf2-d-F	AGGCACGTTTTTATAAAATGAGCC
ChIP-Nrf2-d-R	GTGTGTAATACCACAACATGCTG
ChIP-Nrf2-c-F	AGGTTCTCTTGGGGTTCCCG
ChIP-Nrf2-c-R	GTTCGCAGTCACCCTGAACG
ChIP-Nrf2-b-F	GCCATTCTCGGGCGGTAAAG
ChIP-Nrf2-b-R	CTCGCCCGCGAGATAAAGAG
ChIP-Nrf2-a-F	CCGCGAGCTTCTTGCGTCAG
ChIP-Nrf2-a-R	CCGGCACTCGGTAATCGGCT
